# Lipid raft‐disrupting miltefosine preferentially induces the death of colorectal cancer stem‐like cells

**DOI:** 10.1002/ctm2.552

**Published:** 2021-11-04

**Authors:** So‐Yeon Park, Jee‐Heun Kim, Jang‐Hyun Choi, Choong‐Jae Lee, Won‐Jae Lee, Sehoon Park, Zee‐Yong Park, Jeong‐Heum Baek, Jeong‐Seok Nam

**Affiliations:** ^1^ School of Life Sciences Gwangju Institute of Science and Technology Gwangju Republic of Korea; ^2^ Cell Logistics Research Center Gwangju Institute of Science and Technology Gwangju Republic of Korea; ^3^ Division of Colon and Rectal Surgery Department of Surgery Gil Medical Center Gachon University College of Medicine Incheon Republic of Korea

**Keywords:** cancer stem‐like cells, checkpoint kinase 1, colorectal cancer, lipid rafts

## Abstract

**Background:**

Lipid rafts (LRs), cholesterol‐enriched microdomains on cell membranes, are increasingly viewed as signalling platforms governing critical facets of cancer progression. The phenotype of cancer stem‐like cells (CSCs) presents significant hurdles for successful cancer treatment, and the expression of several CSC markers is associated with LR integrity. However, LR implications in CSCs remain unclear.

**Methods:**

This study evaluated the biological and molecular functions of LRs in colorectal cancer (CRC) by using an LR‐disrupting alkylphospholipid (APL) drug, miltefosine. The mechanistic role of miltefosine in CSC inhibition was examined through normal or tumour intestinal mouse organoid, human CRC cell, CRC xenograft and miltefosine treatment gene expression profile analyses.

**Results:**

Miltefosine suppresses CSC populations and their self‐renewal activities in CRC cells, a CSC‐targeting effect leading to irreversible disruption of tumour‐initiating potential in vivo. Mechanistically, miltefosine reduced the expression of a set of genes, leading to stem cell death. Among them, miltefosine transcriptionally inhibited checkpoint kinase 1 (CHEK1), indicating that LR integrity is essential for CHEK1 expression regulation. In isolated CD44^high^ CSCs, we found that CSCs exhibited stronger therapy resistance than non‐CSC counterparts by preventing cell death through CHEK1‐mediated cell cycle checkpoints. However, inhibition of the LR/CHEK1 axis by miltefosine released cell cycle checkpoints, forcing CSCs to enter inappropriate mitosis with accumulated DNA damage and resulting in catastrophic cell death.

**Conclusion:**

Our findings underscore the therapeutic potential of LR‐targeting APLs for CRC treatment that overcomes the therapy‐resistant phenotype of CSCs, highlighting the importance of the LR/CHEK1 axis as a novel mechanism of APLs.

## Graphical abstract

Miltefosine exhibits preferential cytotoxicity against colorectal cancer stem‐like cells (CSCs). Mechanistically, inhibition of lipid raft/checkpoint kinase 1 (LR/CHEK1) axis by miltefosine releases cell cycle checkpoints and drives the inappropriate mitotic entry in the presence of unresolved DNA damage accumulation, thereby inducing catastrophic mitotic cell death in CSCs. Our findings underscore the therapeutic potential of lipid raft–targeting alkylphospholipids for colorectal cancer treatment that overcomes the therapy‐resistant phenotype of CSCs.

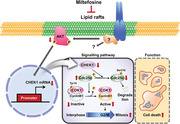

## 1 INTRODUCTION

Colorectal cancer (CRC) is the second leading cause of death worldwide. There are limited therapeutic options for advanced CRC, particularly for tumours resistant to conventional radiation and chemotherapy. Another challenge for CRC treatment arises from latent metastasis and tumour recurrence despite a favorable response to initial therapy. It is widely believed that cancer stem‐like cells (CSCs), a small population of cancer cells with self‐renewal and tumour ‐propagating capacity, survive conventional therapy, retaining the ability of tumour regrowth and metastatic spread.^1^, ^2^ Therefore, therapeutic strategies targeting CSCs will ultimately improve current CRC therapy and complete tumour eradication.

Conventional radiation and chemotherapy induce cell death by introducing excessive replication stress within cancer cells. However, some subpopulations of cancer cells can tolerate higher levels of such stress by exploiting safeguard mechanisms to correctly complete cell cycle phases and repair DNA damage. Accordingly, several promising rationally designed combination therapies that improve such limitations are currently undergoing clinical trials.^3^, ^4^ Many of these therapies target cell cycle checkpoints to ultimately promote the premature entry of tumour cells into mitosis, where they undergo cell death from abnormal mitosis, termed mitotic catastrophe.^5^ Checkpoint kinase 1 (CHEK1) has been a leading target of these therapies currently under intensive investigation. Since cancer cells often have dysregulated G1 checkpoints, particularly due to P53 mutation or deletion, they are considered to be more dependent on the CHEK1‐mediated intra‐S and G2/M checkpoints, providing an exploitable therapeutic vulnerability.^6^, ^7^, ^8^ A number of CHEK1 inhibitors have been developed and are in clinical trials either as single agents or paired with conventional therapy.^9^, ^10^ Notably, recent studies have shown that CSCs display signs of DNA replication stress and are highly dependent on CHEK1 activity, thereby targeting CHEK1 inhibitors.^11^ Thus, further investigation of the regulatory mechanism of CHEK1 in CSCs may broaden and specify the clinical application of CHEK1 inhibitors.

Lipid rafts (LRs) are microdomains of the plasma membrane enriched in cholesterol and sphingolipids and play important roles in various pathophysiological processes by serving as signalling hubs.^12^ LRs have been determined to be major regulators of signal transduction in cancer initiation, progression and metastasis.^13^, ^14^ The structure of LRs is dynamic, resulting in ever‐changing contents of both lipids and proteins. Cholesterol, as a major component of LRs, is critical for the formation and configuration of LR microdomains.^15^ A change in cholesterol levels can result in LR disruption and alter the raft‐associated proteins, such as death receptor proteins,^16^ G‐protein‐coupled receptors,^17^ protein kinases^16^, ^18^, ^19^, ^20^ and calcium channels.^21^ Recently, growing evidence suggests that several CSC markers, such as CD44 and CD133, are enriched in membrane LRs and that their functions are closely associated with LR integrity.^22^, ^23^ The widespread utility of CD44 and CD133 as CSC membrane biomarkers and the great interest in LRs in the stem cell and cancer fields shed light on the concept of “stem cell‐associated LRs” that might hold determinants necessary for maintaining CSC properties; however, the implications of LRs in CSCs remain to be elucidated.

Several anticancer drugs have been reported to suppress the growth and induce apoptosis of tumour cells by disrupting LR integrity. Alkylphospholipids (APLs), synthetic lipase‐resistant analogs of lysophosphatidylcholine, incorporate into LRs on the cellular membrane, thereby inducing the dissociation of essential proteins within LRs, resulting in the disruption of LR‐dependent signalling pathways.^24^ APLs represent a new class of anticancer drugs that do not interact directly with DNA but act on the cell membrane, where they accumulate and induce cytotoxic effects against a wide variety of cancer cells.^25^, ^26^, ^27^ APLs inhibit the proliferation and induce apoptosis of malignant cells while leaving normal cells unaffected and can act as potent sensitizers to conventional chemoradiotherapy.^25^, ^26^, ^28^, ^29^ In parallel, the more recently modified APL derivatives, perifosine or erufosine, have provided significant benefits in combination with conventional therapies in several preclinical and clinical studies.^25^, ^30^, ^31^, ^32^, ^33^, ^34^ Collectively, the anticancer activities of APL drugs may be associated with their LR‐disrupting effects, yet their exact molecular mechanisms still need to be elucidated.

Miltefosine is the prototype APL drug that has been most intensively investigated as an anticancer drug in a wide range of cancer types.^25^ To date, miltefosine is the only APL drug that has been registered for clinical purposes. Miltefosine is used to control the growth of metastatic breast cancer through topical administration to the skin. And incited by high bioavailability, oral treatment of miltefosine has been approved for the eradication of leishmaniasis.^35^, ^36^ Although previous clinical studies of oral miltefosine have shown limited efficacy in CRC patients because of its dose‐limiting gastrointestinal toxicity,^37^ revealing the exact molecular mechanism of miltefosine may help convince the rationale for the further development of APL drugs for cancer treatment.

Here, we investigated the potential role of LRs in CRC by mainly using miltefosine. By quantifying LRs in CRC patient tissues, CRC mouse models and CRC cells, we found preferential enrichment of LRs on CRC cell membranes compared with normal cells. Of note, CSCs contained a higher level of LRs than bulk tumour cells, and LR disruption by miltefosine resulted in CSC death. Through mechanistic investigations, we revealed that disruption of LRs interrupts cell cycle checkpoint regulation. LR disruption by miltefosine transcriptionally inhibited CHEK1 and drove the inappropriate mitotic entry of CSCs in the presence of unresolved DNA damage accumulation, thereby inducing catastrophic mitotic cell death in CSCs. LR disruption exhibits therapeutic effects on CRC metastasis, regrowth and therapy resistance. These findings shed light on CHEK1 reduction as a novel mechanism of LR‐disrupting miltefosine and provide a preclinical rationale to broaden the evaluation of LR‐disrupting drugs that preferentially target colorectal CSCs as well as bulk tumour cells.

## 2 MATERIALS AND METHODS

### 2.1 Ethics, cell line, cell culture and chemicals

All works related to human tissues obtained from CRC patients were preapproved by the Institutional Review Board at Gwangju Institute of Science and Technology (20181106‐BR‐40‐04‐04). All work related to human tissues was conducted in accordance with the Helsinki Declaration and informed consent forms were signed and obtained from all subjects prior to participation. All animal experiments were carried out in accordance with the Institutional Animal Care and Use Committee of the Gwangju Institute of Science and Technology (GIST‐2017‐038). Clinical information for the patient samples used in this study is described in Supplementary Table S1.

Human CRC cell lines including SW48, DLD1, HCT15, HT29, HCT116, Lovo, SW480 and the normal colon cell lines, CCD‐18Co and FHC, were purchased from the American Type Culture Collection (ATCC, Manassas, VA, USA) and Korean Cell Line Bank (KCB, Seoul, Republic of Korea). P53 knockout HCT116 cells were kindly gifted by Prof. Han‐Woong Lee (Yonsei University, Seoul, Republic of Korea). Human CRC cells were cultured in RPMI 1640 (RPMI) with 5% foetal bovine serum (FBS), 100 U/mL penicillin/streptomycin (Welgene Inc, Daegu, Republic of Korea) at 37°C in a 5% CO_2_ humidified incubator. Patient‐derived primary CRC cells and CCD‐18Co cells were cultured in Dulbecco's modified Eagle's minimal essential Medium (DMEM) (Welgene) with 10% FBS. FHC were cultured in DMEM:F12 (Welgene) with 10% FBS. All experiments were performed with cells between passages 2 and 8. All cell lines were routinely tested for mycoplasma contamination every 6 months using an e‐Myco™ Mycoplasma PCR detection kit (iNtron Biotechnology, Seongnam, Republic of Korea).

Miltefosine, 5‐fluorouracil, oxaliplatin, AZD7762, methyl‐β‐cyclodextrin (MβCD), nystatin, simvastatin and perifosine were obtained from Sigma‐Aldrich (St. Louis, MO, USA). MK‐2206 (hydrochloride) was obtained from Cayman Chemical (Ann Arbor, MI, USA). Cells were exposed to radiation using a soft X‐ray irradiator (Model M‐100, SOFTEX, Tokyo, Japan). Human EGF and murine FGF were purchased from Peprotech, Inc. (London, UK).

### 2.2 Isolation of LRs from cancer and normal cells

LR isolation was performed by using discontinuous sucrose gradient centrifugation as previously described.^38^ Briefly, CRC cells (HT29) or normal colon cells (FHC) were grown in 75T flasks and scraped into 1.4 mL of membrane raft isolation buffer (1% Triton X‐100 and 1 mM phenyl‐methylsulfonyl fluoride in TNEV buffer; 10 mM Tris–HCl, pH 7.5, 150 mM NaCl, 5 mM ethylenediaminetetraacetic acid (EDTA), 1 mM Na_3_VO_4_). Homogenization was carried out using a homogenizer (10–15 strokes). Then, 1 mL of homogenate was mixed with 1 mL 85% (w/v) sucrose prepared in TNEV buffer and was transferred to the bottom of a Beckman 14 × 89 mm centrifuge tube. A 5–35% discontinuous sucrose gradient was generated by loading 6 mL of 35% (w/v) sucrose and 4 mL of 5% (w/v) sucrose. Once the centrifuge tube is loaded using a proper rotor (SW 41 Ti), the sample was centrifuged at 38,800 rpm (257,000*g*, at *r*_max_) for 18 h, 4°C. After ultracentrifugation, 1 mL fractions were collected from the top (fraction 1) of the gradient to the bottom (fraction 12). The LR‐enriched fractions were confirmed by Western blot analysis based on the quantity of Flotillin‐1, Caveolin‐1 and GM1 (positive controls for LRs).

### 2.3 Ganglioside extraction from LRs

To enrich LRs from cell lysates, fractions 4–6 collected from sucrose gradient centrifugation were pooled and ultracentrifuged for 2 h at 100,000 *g*. LR was pelleted and washed with 0.2 M sodium carbonate (pH 11). For ganglioside extraction from LR, the pellets were solubilized in 1 mL water/chloroform/methanol (3:4:8; v/v/v). Incubation of the mixtures was conducted in a bath sonicator for 30 min at 10°C, and they were centrifuged at 8800 *g* for 2 min at 4°C. After transferring the supernatant to a new tube, pellets were re‐extracted with 1 mL chloroform/methanol (1:1; v/v) and 1 mL chloroform/methanol (2:1; v/v). Supernatants of each extraction solution were dried in Speed‐Vac, and they were integrated by dissolution with 1 mL chloroform/methanol (2:1; v/v). For Folch partitioning, 200 μL of 0.1 M potassium chloride was added, followed by centrifugation at 8800 *g* for 2 min at 4°C. The upper aqueous layer was collected, and the lower layer was reextracted with 300 μL of methanol/0.1 M potassium chloride (1:1, v/v). Gangliosides were further purified using a 1‐cc Sep‐Pak C18 cartridge (Waters, Milford, MA, USA). Briefly, 3 mL of methanol, 3 mL of chloroform/methanol (1:1; v/v) and 2 mL of water were used for washing cartridges. Samples were loaded into the cartridge and washed with 3 mL of water. Gangliosides were eluted with 1 mL of chloroform/methanol (1:1; v/v) and 1 mL of water/chloroform/methanol (3:4:8; v/v/v). Eluates were vacuum‐dried in Speed Vac and stored at −80°C until liquid chromatography with tandem mass spectrometry (LC‐MS/MS) analysis.

### 2.4 LC‐MS/MS data acquisition for ganglioside

Samples were suspended in isopropanol/water/acetonitrile (45:28:27, v/v/v) for LC‐MS analysis. A Q Exactive Focus mass spectrometer (Thermo Scientific, Bremen, Germany) connected in‐line with a Dionex Ultimate 3000 UPLC was used to identify ganglioside species. Gangliosides were separated with a Hypersil GOLD column (100 mm × 2.1 mm, 1.9 μm) using mobile phase buffer A, 0.1% formic acid, 10 mM ammonium acetate, 60% acetonitrile and buffer B, 0.1% formic acid, 10 mM ammonium acetate in 90% isopropanol and 10% acetonitrile. At a flow rate of 0.25 mL/min, gangliosides were eluted with a linear gradient from 30 to 50% buffer B in 1 min and 50 to 70% buffer B in 6 min. Then, buffer B reached 99% in 6 min, followed by equilibration of the system at 30% buffer B for 2 min. Data‐dependent analysis was conducted in negative mode with MS1 spectra at a resolution of 70,000 over the scan range of 500–2500 *m/z*. The automatic gain control target was 1 × e6, and the top 10 most abundant ions were selected for MS/MS spectra. MS/MS spectra were obtained using high‐energy collision dissociation (HCD) with normalized collision energies of 35% and a resolution of 17,500. The maximum ion injection times of the survey scan and the MS/MS scan were 100 and 50 ms, respectively. Furthermore, analysis was performed with a 3.5‐kV spray voltage, 20 au sheath gas flow rate, 5 au AUX gas flow rate, 1 au sweep gas flow rate, 400°C heater temperature, 350°C capillary temperature, 40°C column chamber temperature and 6°C sample tray temperature.

### 2.5 Data analysis for ganglioside

Lipid identification was performed with LipidSearch 4.2 software (Thermo Scientific), and parent search mode was used for gangliosides (GM1, GM2, GM3, GD1a, GD1b, GD2, GD3, GT1a, GT1b, GT1c, GT2, GT3, GQ1c and GQ1b). The following parameters for the search were set: database, HCD; experiment type, LC‐MS; parent *m*/*z* tolerance, 5 ppm; merge range, 0.1 min; minimal peak width, 0.0 min; threshold type, absolute; intensity threshold, 100,000.0; recalculate isotope, ON; Retention time (R.T.) interval, 0.0 min; execute quantification, ON; *m*/*z* tolerance, −5.0/+5.0; tolerance type, ppm; R.T. range (min), −1.0/+1.0; top‐rank filter, ON; main node filter, main isomer peak; and fatty acid priority, ON. Adducts used in the search were −H, +HCOO, +CH_3_COO and −2H.

Quantitation of gangliosides and glycosphingolipids was performed by extracted ion chromatograms (XIC) based on identification by LipidSearch 4.2 software. The area under the curve (AUC) of each peak from XIC was used for quantitation, and each lipid class was compared by summing the AUC of individual ions in the same class. The AUC of identified gangliosides was normalized by the total AUC of gangliosides in each sample and accounted for as the relative abundance.

### 2.6 Immunofluorescence staining and quantification

Samples were fixed with 4% paraformaldehyde for fluorescent staining. Samples were permeabilized with 0.3 mol/L glycine and 0.3% Triton X‐100, and nonspecific binding was blocked with 2% normal swine serum (DAKO, Glostrup, Denmark). The staining and quantification were performed as described previously,^39^ using Alexa Fluor 488‐conjugated cholera toxin‐B (CTxB, Thermo Fisher, Wilmington, DE, USA), DeadEnd™ Fluorometric TUNEL system (Promega, Madison, WI, USA), anti‐CD44 (1:500, Novus, Littleton, CO, USA), anti‐CHEK1 (1:500; Abcam, Cambridge, MA, USA) antibodies. Secondary antibodies, Alexa Fluor 488‐ or 555‐conjugated rabbit IgG (1:1000; Thermo Fisher), were used to visualize target proteins. Samples were examined by fluorescence microscopy (Axio Imager 2, Zeiss, Oberkochen, Germany). For quantitation, an arbitrary threshold was set to distinguish specific from background staining, and this same threshold setting was applied to all the samples analysed. The calculation was based on red or green fluorescence intensity divided by the intensity of DAPI‐stained nuclei, in three randomly selected fields for each specimen from a total of three independent experiments. Quantifications were performed by using Image Pro Premier 9 (Media Cybermetics, Rockville, MD, USA).

### 2.7 Animal study

Animal experiments were performed to evaluate the antitumour effect of miltefosine on primary tumour growth, secondary tumour regrowth and tumour metastasis as previously described in our studies with slight modifications.^39^, ^40^ Briefly, to evaluate the antitumour effects of miltefosine on tumour growth, HT29 (1 × 10^6^ cells/mouse mixed with Matrigel) were injected into the inguinal folds of NSG mice (NOD.Cg‐Prkdc scid IL2rg tm1 Wjl/Szl, Jackson Laboratory, Bar Harbor, ME, USA). Tumours were allowed to reach in size approximately 100 mm^3^, and then the mice were randomly divided into two groups for phosphate‐buffered saline (PBS) or miltefosine treatment (*n* = 8 or 11/group, 10 mg/kg, intraperitoneal, daily). The tumour volumes were measured twice a week and estimated by using the formula: tumour volume = length × width^2^/2, where length represents the largest tumour diameter and width represents the perpendicular tumour diameter. On the 21st day after inoculation, primary tumours were removed and dissociated into single cells to compare the tumour regrowth potential of PBS or miltefosine‐treated tumour cells. After depleting stromal cells with a mouse cell depletion kit (Miltenyl Biotec, Bergisch Gladhac, Germany), the isolated tumour cells were subcutaneously injected into the inguinal folds of NSG mice with Matrigel at various dilutions (12,500, 25,000, 50,000 or 10,0000 cells per mouse; *n* = 6/group). Tumours formed by this injection were monitored twice a week, and tumour volumes were measured by a caliper. On the 28th day after inoculation, the definitive ratio of tumour‐bearing mice was determined by necropsy. The frequency of stem cells was calculated by the extreme limiting dilution assay webtool (http://bioinf.wehi.edu.au/software/elda).

For the liver metastasis mouse model, luciferase‐tagged HT29 cells (HT29‐luc, 1 × 10^6^ cells/mouse) were inoculated into the spleen followed by splenectomy, and the surviving cells that grew in distant organs then contributed to the formation of liver metastases. Since the day after the cancer cell injection, miltefosine or PBS was administered daily (10 mg/kg, intraperitoneal, *n* = 9/group) and the extent of liver metastasis was routinely monitored weekly by visualizing luciferase activity for 35 days with an IVIS lumina imaging system (Xenogen Co., Alameda, CA, USA). After sacrifice, the livers were removed, formalin‐fixed and paraffin‐embedded to definitely determine liver metastasis. The same experiment was repeatedly performed to compare the survival rate of mice between the PBS‐ and miltefosine‐treated groups (*n* = 7/group). The daily treatment with miltefosine and PBS was continued until death.

### 2.8 Intestinal organoid culture

Single cells were isolated from the intestinal crypts of 20‐week‐old wild‐type mice or from the intestinal polyps of 20‐week‐old APC^Min/+^ mice and cultured as described in a previous report with slight modifications.^41^ Briefly, intestinal crypts were isolated from normal intestines by ethylenediaminetetraacetic acid chelation buffer. Then, by using collagenase, isolate crypts were dissociated into single cells. Then, 100 cells were then mixed with 10 μL of Matrigel (Corning® growth factor reduced basement membrane matrix, #356231, Corning, USA) and plated in 96‐well plates. After polymerization of Matrigel, 100 μL of IntestiCult™ organoid growth medium (mouse, #06005, STEMCELL Technologies, Vancouver, Canada) was added. From the day of seeding, the growth and morphology of organoids were observed every day. To compare the selectivity of anticancer treatment, drugs were applied to the culture media after cysts were generated on the third day of organoid seeding. The viability of organoids was measured and compared by resazurin‐based Cell Titer Blue assay (Promega, Leiden, the Netherlands) on the ninth day of organoid seeding.

### 2.9 Sphere forming assay

Cells (1 × 10^4^ cells/well) were suspended in sphere culture medium; serum‐free medium 1640 (Welgene Inc, Daegu, Republic of Korea) containing B27 (Invitrogen), 20 ng/mL human epidermal growth factor (hEGF, PeproTech EC, London, UK), 20 ng/mL human fibroblast growth factor (hFGF, PeproTech EC) and 4 μg/mL heparin (Sigma‐Aldrich). Suspended cells were then seeded in poly‐2‐hydroxyethyl methacrylate (poly‐HEMA)‐coated six‐well plates. To evaluate the stemness reducing the effect of miltefosine, treatment was applied to cells every other day. Spheres reaching size > 100 μm from each replicate well were counted under an inverted microscope at 40× magnification using Image‐Pro Premier 9.0 (Media Cybernetics, MD, USA). The frequency of sphere‐forming efficiency was calculated as follows: the number of spheres formed/number of plated cells. The viability of spheres was measured and compared by resazurin‐based cell titer blue assay (Promega, Leiden, the Netherlands) on the seventh day of sphere seeding.

### 2.10 Isolation of primary CRC cells from patient tumours, fluorescence‐activated cell sorting analysis and validation

Patient‐derived primary CRC cells were isolated and collected from primary tumours of CRC patients using the tumor cell isolation kit (Milteny Biotec, Bergisch, Germany) as described previously.^40^ Briefly, primary tumours were minced completely until nearly liquid using scalpels and incubated with 0.1% collagenase type IV (Sigma‐Aldrich, St. Louis, MO, USA) for 30 min. Contaminating red blood cells were lysed with RBC lysis buffer (Sigma‐Aldrich), and live cells were gated with propidium iodide (Sigma‐Aldrich) staining. EpCAM+ epithelial cells were isolated using magnetic beads. They were considered CRC cells, and their epithelial characteristics were validated by analysing specific surface markers through immunofluorescence assays based on EpCAM (Cell Signaling Technology, Beverly, MA, USA), CK‐7 (DAKO, Carpinteria, CA, USA) and vimentin (BD Biosciences). EpCAM, an epithelial marker, was expressed in patient‐derived CRC cells, while CK‐7 (a breast epithelial marker) and vimentin (a fibroblast marker) were not expressed in these cells.

### 2.11 Anchorage‐independent growth assay

Agar (0.35%, 1 mL) in culture media was added to the bottom layer of each well in a 12‐well plate. For the top layer in each well, 1 × 10^3^ cells were resuspended in 1 mL of a mixture comprising 0.2% agar and RRMI 1640 media. The cells were incubated at 37°C with 5% CO_2_ for 2 weeks, after which the colonies were stained with crystal violet, photographed by inverted phase‐contrast microscopy (Leica, Wetzlar, Germany) and counted using ImagePro premier 9 (Image‐Pro Premier 9.0, Media Cybernetics, MD, USA).

### 2.12 Apoptosis assay

Quantitative analysis of the apoptotic population was performed using the Annexin V‐fluorescein isothiocyanate (FITC) apoptosis detection kit I (BD Biosciences). After the miltefosine treatment or siRNA transfection, cells were collected and washed twice with cold PBS. The cells were resuspended in binding buffer (1 × 10^6^ cells/mL), and 100 μL of suspension was transferred to a tube and mixed with 5 μL of FITC Annexin V and propidium iodide (PI). Then, the mixture was incubated at room temperature for 15 min in the dark. After incubation, 400 μL of 1× binding buffer was added and then analysed by flow cytometry (BD AccuriTM C6, BD Biosciences)

### 2.13 Microarrays and bioinformatics analysis

An Illumina microarray was performed as previously described.^42^ HT29 cells were treated with DMSO or miltefosine (1 μM) for 48 h, and RNA extraction from DMSO‐ or miltefosine‐treated cells was performed using an RNeasy minikit (Qiagen, Valencia, CA, USA). The differential expression of genes (DEGs) upon miltefosine treatment was analysed using an Illumina HT‐12 BeadChip. The quality of RNAs was checked with an Agilent Bioanalyser (RNA 6000 nanokit), and the only RNA with an RNA integrity number (RIN) > 8 was accepted for RNA amplification. Amplification of RNA, hybridization, image processing, and raw data extraction were performed using protocols suitable for each platform. After quantification of DEGs with quantile normalization, the significance of DEGs was determined by the *t*‐test. The obtained *p*‐values were corrected for multiple testing by calculating estimated false discovery rates (FDRs) using the Benjamino–Hochberg method.

To identify the related functions and diseases associated with DEGs and to identify the potential upstream regulators, ingenuity pathway analysis (IPA; Ingenuity® System, CA, USA) was performed. The genes with a significant difference were used to predict the potential signalling pathways using the web‐based bioinformatics tool, Enrichr (http://amp.pharm.mssm.edu/Enrichr/). The results from this analysis showed the potential protein kinases associated with miltefosine together with their *p*‐value, combined score and the target genes of the kinases that matched the input genes. A combined score multiplies the log of the *p*‐value computed with Fisher's exact test by the *z*‐score computed by assessing the deviation from the expected rank.^43^ Gene set enrichment analysis (GSEA) was performed as described in our previous report.^44^ Briefly, GSEA was performed by using the microarray data of DMSO‐ and miltefosine‐treated HT29 cells. The entire gene lists were subjected to GSEA. The analysis included gene sets from MSigDB pathways and C2: curated gene sets (c2.all.v6.2.symbols.gmt), and an FDR *q*‐value < 0.05 was set as the significance threshold. The DEG list is attached in Supplementary Table S3.

### 2.14 Cell cycle assay

Cell cycle analysis by quantification of DNA contents was performed by PI staining and flow cytometry analysis. After miltefosine treatment or siRNA transfection, cells were harvested and fixed in 70% cold ethanol for at least 30 min at 4°C. After fixation, cells were washed. To remove any nonspecific staining, 50 μL of 100 μg/mL RNase was added to the sample and then cells were stained with propidium iodide (50 μg/mL) for flow cytometry analysis.

### 2.15 Cell viability assay

CRC cells were seeded in 96‐well plates (1 × 10^4^ cells/well). After 24 h of incubation, the cells were treated with an increasing concentration of miltefosine for 48 h. Cell viability was assessed by thiazolyl blue tetrazolium bromide (MTT; Sigma‐Aldrich) according to the manufacturer's instructions. The relative cell viability was measured at a wavelength of 420 nm using an Epoch microplate reader (Biotek, Winooski, VT, USA).

### 2.16 Comparison of relative sensitivity to 5‐fluorouracil, oxaliplatin or radiation

Relative sensitivities to 5‐fluorouracil or oxaliplatin were compared by determining the half‐maximal inhibitory concentration (IC_50_) values based on reductions in cell viability. Briefly, cells were seeded in 96‐well plates (5000 cells/well) and incubated for 24 h for attachment. Then, the cells were treated with 5‐fluorouracil or oxaliplatin at various concentrations and incubated for 48 h. Cell viability was measured by staining with MTT (Sigma‐Aldrich), and the absorbance was measured using a microplate spectrophotometer (Bio‐Tek Instruments). In addition, the sensitivity of the cells to radiation was measured using traditional methods.^45^ Briefly, the survival fraction of irradiated cells was estimated with clonogenic assays, and the radiation‐related biological parameters and statistical significance were then analysed using the linear‐quadratic model GraphPad Prism v7.05 software (GraphPad, La Jolla, CA, USA).

### 2.17 Clonogenic assay

Cells were treated with miltefosine (1 μM) or DMSO control for 48 h and exposed to escalating doses of radiation. Immediately after RT exposure, cells were plated in 12‐well plates at 500 cells/well for 1 or 2 weeks at 37°C in a humidified incubator with 5% CO_2_. Then, the cells were stained with crystal violet and dried overnight and colonies containing >50 cells were counted using Image‐Pro Premier 9.0 (Media Cybermetics). The surviving fraction after irradiation was calculated as the number of colonies/(number of cells seeded × PE). The platting efficiency PE was calculated using the number of colonies/number of cells seeded in the nonirradiated cells.

### 2.18 Fluorescence‐activated cell sorting

Fluorescence‐activated cell sorting (FACS) analysis and cell sorting were performed using BD Accuri™ C6 (BD Biosciences) and FACS Aria instruments (BD Biosciences), respectively. FACS data were analysed using FlowJo software (TreeStar, Ashland, OR, USA). Antibodies against the following proteins were used: PE‐conjugated anti‐CD133 (dilution 3/200, Becton Dickinson), APC‐conjugated anti‐CD44 (dilution 1/200, Becton Dickinson), PE‐conjugated anti‐ALDH1 (dilution 3/200; Cell Signaling Technology) and anti‐CHEK1 (dilution 1/200; Abcam). LR levels on the cell membrane were visualized using an Alexa Fluor 488‐ or Alexa Fluor 555‐conjugated CTxB antibody (dilution 1/1000; Invitrogen). APC‐ or FITC‐conjugated secondary antibodies were applied to nonconjugated primary antibodies. FACS gates were established by staining with an isotype control antibody. Intracellular permeabilization was performed using a BD Pharmingen™ transcription factor buffer set (BD Biosciences) to detect intracellular markers, for example, CHEK1. Briefly, after cells were stained with antibodies against cell surface markers, cells were fixed and permeabilized using fixation/permeabilization buffers from the buffer set described above. The live/dead cell analysis was conducted using the MAX‐View™ live/dead cell staining kit (Biomax, Seoul, Republic of Korea).

### 2.19 In vitro limiting dilution assay

To examine the self‐renewal ability, cells were seeded in sphere culture conditions (poly‐HEMA‐coated plates; poly 2‐hydroxyethyl methacrylate, Sigma #P3932) at varying cell densities of 10, 100, 500, 1000, 5000 and 10,000. After 14 days of culture, the number of wells with spheres was counted (*n* = 12/group) and analysed by the extreme limiting dilution assay (ELDA) webtool (http://bioinf.wehi.edu.au/software/elda) as described in a previous report.^40^

### 2.20 Protein isolation and western blot analysis

Cells were lysed in RIPA buffer (20 mmol/L Tris–HCl, pH 7.5, 200 mmol/L NaCl, 1% Triton X‐100, 1 mmol/L dithiothreitol) containing protease inhibitor cocktail (Roche). Protein concentration was measured with a protein assay kit (Bio‐Rad) following the manufacturer's protocol. Total protein was subjected to SDS–PAGE and transferred to a polyvinylidene difluoride membrane. The blot was probed with primary antibodies and secondary antibodies. β‐Actin was used as a loading control. Subsequently, the blots were washed in TBST (10 mmol/L Tris–HCl, 50 mmol/L NaCl and 0.25% Tween‐20) and incubated with a horseradish peroxidase‐conjugated secondary antibody. The presence of target proteins was detected using enhanced chemiluminescence reagents (Thermo‐Scientific). The list of antibodies is given in Supplementary Table S4. The blots were then developed with FluorChem E (ProteinSimple, San Jose, CA, USA) using ECL reagent (Thermo Fisher Scientific, Rockford, IL, USA). Band intensities were quantified by MultiGauge ver. 3.0 (Fujifilm, Tokyo, Japan) and normalized to β‐actin intensity.

### 2.21 Promoter‐reporter assay

A promoter–reporter construct for CHEK1 (Genecopoeia, Rockville, MD, USA) was used to perform luciferase reporter experiments. To measure promoter activity, the reporter construct was transfected into cells by using Lipofectamine 2000 (Invitrogen, Carlsbad, CA, USA) according to the manufacturer's instructions. After 24 h of incubation, cells were treated with miltefosine or Akt inhibitor and then its luminescence was detected by a luminometer (Glomax, Promega, Sunnyvale, CA, USA) according to the manufacturer's instruction. To examine the CHEK1 promoter activity in CSC‐enriched spheres, the cells transfected with CHEK1 promoter–reporter constructs were detached from the culture plate and re‐suspended in sphere culture condition medium as described in the sphere‐forming assay. The total value of reporter activity in each well was normalized to β‐galactosidase activity as previously described.^40^

### 2.22 Small interfering RNA‐mediated knockdown

CHEK1 siRNA and a nonspecific negative control siRNA (Bioneer, Daejeon, Republic of Korea) were used to generation CHEK1 knockdown cells. Cells were transfected with CHEK1 siRNA and control siRNA in media (serum‐, phenol‐, antibiotic‐free) with Lipofectamine™ 2000 (Invitrogen) according to the manufacturer's instructions. Knockdown efficiency was confirmed based on the relative expression of mRNA, which was detected by reverse transcription polymerase chain reaction (PCR) and real‐time polymerase chain reaction (RT‐qPCR). The siRNA sequences are listed in Supplementary Table S5.

### 2.23 Establishment of CHEK1‐overexpressing cell

The CHEK1 overexpression vector (NM_001274.5, cat # EX‐Z6226‐M68) and control empty‐vector (cat # EX‐NEG‐M68) were purchased from GeneCopoeia™ (Rockville, MD, USA). According to the manufacturer's recommendations, vectors were transfected into HT29 cells by Lipofectamine 2000 (Invitrogen). Antibiotic selection was performed to obtain a stable CHEK1‐overexpressing clone. The efficiency of overexpression was examined at both mRNA and protein levels by RT‐qPCR and Western blot assay, respectively.

### 2.24 Relative mRNA expressional analysis by RT‐qPCR

Total RNA was extracted using RNAiso (Takara, Shiga, Japan), and RNA purity was verified by measuring the 260/280 absorbance ratio. First‐strand cDNA was synthesized using PrimeScriptTM first strand cDNA synthesis kit (Takara), and one‐tenth of the cDNA was used for each PCR mixture containing Power SYBR® Green PCR Master Mix (Applied Biosystems). Real‐time (RT)‐PCR was performed using a StepOnePlus real‐time PCR system (Applied Biosystems). Relative mRNA expression of selected genes was normalized to Cyclophilin A (PPIA) and quantified using the ddCt method. The sequences of the PCR primers are listed in Supplementary Table S6.

### 2.25 Single‐cell gel electrophoresis (neutral comet assay)

Neutral comet assays were performed as previously described.^46^ Briefly, cells were mixed with 0.5% low melting point agarose (Sigma‐Aldrich), spread on CometSlide microscope slides (Trevigen, Gaithersburg, MD, USA) and subjected to lysis. After electrophoresis, the slides were stained with ethidium bromide and comets were scored (50 cells per treatment) under a fluorescence microscope (Axio Imager 2, ZEISS, Oberkochen, Germany); the data were then analysed using Image‐Pro Premier 9.0 (Media Cybernetics). The comet parameter (olive tail moment) reflects the amount of unrepaired DNA released from the cells.

### 2.26 γH2AX and mitotic catastrophe analysis

Radiation or miltefosine treatment was applied to seeded cells to induce genotoxic stress. Upon treatment, the phosphorylated form of **γ**H2AX was measured by flow cytometry (BD AccuriTM C6) using the anti‐**γ**H2AX antibody (1:200, BD Biosciences). The FACS gates were established by staining with an isotype antibody. FACS data were analysed using FlowJo software (TreeStar).

Cells were seeded on poly‐l‐lysine and collagen I‐coated cover glasses and treated with radiation or miltefosine. Thereafter, cells were fixed in 4% formalin. Cells were permeabilized, blocked and stained with anti‐**γ**H2AX (1:200; Abcam) and anti‐p‐histone H3 S10 (p‐HisH3, 1:200, Cell Signaling Technology) for overnight followed by incubation with the corresponding secondary antibodies. All nuclei were stained with DAPI to count the total cell number. Before quantification, an arbitrary threshold was set to distinguish specific from background staining, and this same threshold setting was applied to all the samples analysed. A distinct mitotic cell population expressing the phosphorylated form of histone H3 (p‐HisH3^+^) with intense nuclear γH2AX staining is likely to represent cells in the initial stages of mitotic catastrophe (mitotic γH2AX). A separate population of mitotic cells characterized by the loss of membrane integrity and fragmented morphology with one or more micronuclei is likely to represent cells facing tragic cell death (mitotic catastrophe). Separate populations of mitotic **γ**H2AX^+^ and mitotic catastrophes were quantified as percentages of cells among the p‐HisH3^+^ mitotic cells. The quantifications were performed in three randomly selected fields for each specimen from a total of six independent experiments. The percentages of mitotic γH2AX^+^ cells and mitotic catastrophes are presented as the mean ± SEM (*n* = 6/group). Statistical significance was calculated by one‐way analysis of variance (ANOVA) with GraphPad Prism v7.05 software (GraphPad).

### 2.27 Statistical analyses

All Western blots and immunohistological images are representative results from at least three independent biological replicates. Statistical calculations were derived from at least three independent experiments and analysed as previously described,^47^ by Student's *t*‐test (unpaired, two‐tailed) for two groups or by one‐way ANOVA with Dunnett's multiple comparison test for groups of three or more using GraphPad Prism v7.05 software (GraphPad). For comparison of tumour and matched‐up normal tissues, statistical calculations were analysed by the paired *t*‐test. Statistical significance of tumour growth in an animal model was determined by two‐way repeated‐measures ANOVA followed by Bonferroni post‐tests. Survival curves were plotted using the Kaplan–Meier method and compared using the log‐rank test. The correlation between CHEK1 and CD44 expression was analysed by Spearman's correlation coefficient. Data are presented as the mean ± standard error of the mean (SEM). *, ** and *** indicate *p* < .05, *p* < .01 and *p* < .001, respectively.

## 3 RESULTS

### 3.1 Miltefosine exhibits preferential cytotoxicity toward CRC cells

LR elevation has been reported in various types of cancer, including melanoma,^48^ breasts,^49^ and prostate cancer,^49^ as assessed by LR‐staining probes. The higher presence of LRs in cancer cells is important because LRs can harbour receptors and regulatory molecules and thus act as signalling platforms responsible for the aggressive phenotype of cancer cells.^12^, ^15^ However, despite the abundance of literature available on the biology and pathobiology of LRs in cancer,^14^, ^50^ only a few dedicated papers currently address the importance of these membrane domains in the progression of CRC.^51^ Thus we decided to examine CRC patient tissues to compare LR levels between CRC patient tissues and matched normal tissues. Cholera toxin B subunit (CTxB) has been widely used for visualizing LR levels in tissue specimens or cells incited by its higher affinity to ganglioside GM1, a marker for LRs^52^; however, we needed to confirm whether GM1 is preferentially localized on LRs before analysing patient specimens with CTxB.

When we isolated the LR fraction from the human CRC cell line (HT29) through discontinuous sucrose gradient centrifugation,^38^ we confirmed that GM1 preferentially appeared in the LR fraction (indicated by fraction #4‐6) with concomitant enrichment of flotillin‐1 and caveolin‐1 (positive controls for LRs^38^), suggesting the preferential localization of GM‐1 in LRs (Supplementary Figure S1A**)**. Additionally, the further LC‐MS/MS analysis identified the presence of GM‐1 in LRs (Supplementary Figure S1B, Supplementary Table S2). In this procedure, we compared the ganglioside composition between normal LRs extracted from a normal colon cell line, FHC and cancer LRs extracted from HT29 cells. The results showed that normal LRs contained more diverse ganglioside compositions than cancer LRs (Supplementary Figure S1B). Interestingly, the relative abundance of GM1 was significantly increased in LRs from cancer cells (0.15 %) than LRs from normal cells (0.06 %). Collectively, our Western blot and LC‐MS/MS analyses confirmed the presence of GM1 in the LR fraction, corroborating GM1‐binding CTxB as a putative LR marker.

**FIGURE 1:**
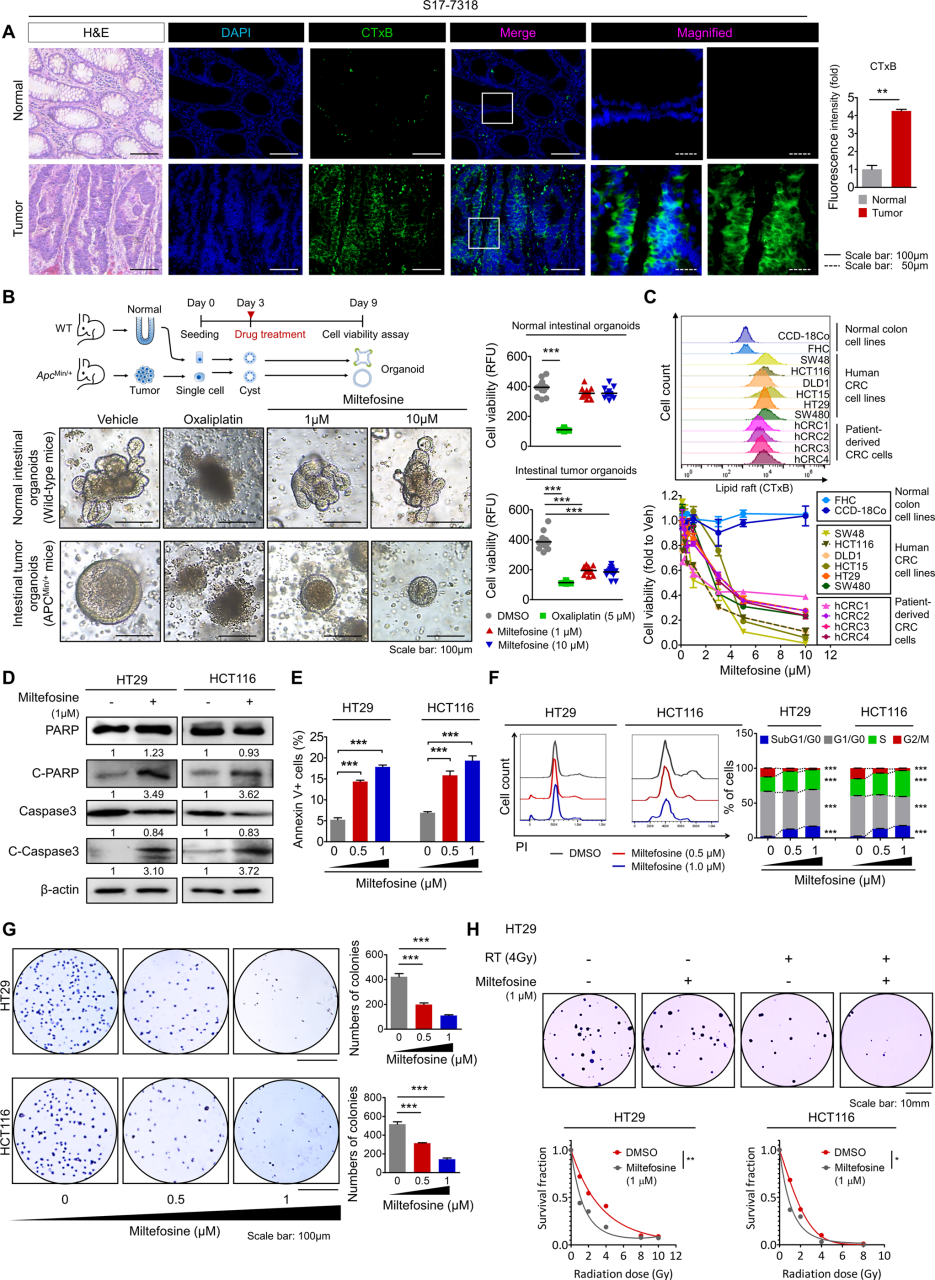
Miltefosine preferentially targets CRC cells. (A) The elevated levels of LRs in tumour tissues versus in matched normal tissues were obtained from three CRC patients (S17‐7318, S17‐12449 and S17‐6221). H&E staining and immunofluorescence staining for DAPI (blue) and CTxB (green) with respective merged and magnified images. LRs were visualized by using the Alexa 488‐conjugated CTxB, which binds to ganglioside GM1, an LR component. Relative fluorescent intensity of CTxB was quantified by using Image pro premier 9 (Media Cybermetics) with normalization to DAPI intensity. Quantification was performed in three randomly selected fields for each patient specimen. The bar graph shows the mean ± SEM (*n* = 3/group). Statistical significance was determined by the paired *t*‐test. (B) Cytotoxic effect of chemotherapy drug (oxaliplatin) and miltefosine in mouse intestinal normal or tumour organoids. Representative images (left) of normal intestinal organoids derived from WT mice and intestinal tumour organoids derived from *Apc* ^Min/+^ mice. To compare the selectivity of anti‐cancer treatment, drugs were applied into the culture media after cysts being generated on third day of organoid seeding; oxaliplatin (5 μM) and miltefosine (1 and 10 μM). The quantitative analysis (right) of organoid growth was performed on the ninth day of organoid seeding (*n* = 9/group) based on viability measurement using resazurin‐based cell titer blue (Promega, Leiden, the Netherlands) (C) Increased LR levels in CRC cells than in normal cells and preferential cytotoxicity of miltefosine against CRC cells after 48‐h treatment. A panel of histograms (top) show the relative LR levels (CTxB) in multiple human cells; normal colon cell lines (FHC, CCD‐18Co), human CRC cell lines (SW48, HCT116, DLD1, HCT15, HT29, SW480) and patient‐derived primary CRC cells (hCRC1, hCRC2, hCRC3, hCRC4). The cell viability (bottom) was determined by the MTT assay after 48‐ h treatment of miltefosine. (D) Protein expression of apoptotic signalling components after 48‐h miltefosine treatment. Values below each lane indicate the relative band intensity of target protein normalized to β‐actin as fold to control lane. (E) Flow cytometry analysis of apoptotic cells after 48 h of miltefosine treatment. Apoptotic cells were Annexin V^+^. (F) Cell cycle analysis after 48 h of miltefosine treatment. The subpopulations of cells represented are subG1/G0 phase (blue), G1/G0 phase (gray), S phase (green) and G2/M phase (red). (G) Reduction in the AIG potential of CRC cells by miltefosine treatment. Cells were treated with the indicated concentrations of miltefosine for 48 h. Then, the cells were resuspended and seeded on agar‐coated plates to determine the AIG potential. After 2 weeks of incubation, colonies were stained with crystal violet and counted using Image‐Pro Premier 9 software (Image Pro Premier 9.0). (E)–(G) Bar graphs showing the means ± SEM (*n* = 5/group). Statistical calculations were analysed using one‐way ANOVA with Dunnett's multiple comparison test. (H) Comparison of relative sensitivity to RT after miltefosine or DMSO treatment. Cells were treated with miltefosine (1 μM) or DMSO control for 48 h and exposed to an escalating dose of radiation. Right after RT exposure, clonogenic assays were performed (*n* = 3/group). The representative images (top) of surviving colonies. The surviving fraction was calculated as the number of colonies/(number of cells seeded × PE). The platting efficiency PE was calculated using the number of colonies/number of cells seeded in the non‐irradiated cells. The generation of cell survival curves (bottom) and statistical analysis were conducted based on a linear‐quadratic model using GraphPad Prism v7.05 software (GraphPad). *, ** and *** indicate *p *< .05, *p* < .01 and *p* < .001, respectively

Through staining tissue specimens with CTxB and subsequent observation with fluorescence microscopy, we discovered a significant elevation in LR levels in CRC patient tissues compared to matched normal counterpart tissues (Figures 1A and Supplementary Figure S1C). To obtain greater insight into this finding, we compared the LR levels in the normal intestines of wild‐type (WT) mice and the adenomatous intestines of adenomatous polyposis coli (*Apc*)^Min/+^ mice. We repeatedly observed apparently higher levels of LR in adenomatous polyps than in the normal region of mouse intestines (Supplementary Figure S1D). Since LRs function as signalling platforms responsible for the critical facet of cancer progression, including proliferation and survival,^12^, ^14^ we examined the therapeutic effect of LR disruption in CRC by using miltefosine, a prototype synthetic APL drug, which can target and interrupt the structure of LRs due to its similarity to endogenous phospholipids.^53^ We first isolated single cells from the normal intestines of WT mice and adenomatous intestines of *Apc* ^Min/+^ mice and cultured them to generate normal intestinal organoids and intestinal tumour organoids, respectively. On day 3, when cells formed a cyst‐like morphology in the organoid culture conditions, we began treatment with miltefosine or oxaliplatin, a platinum‐containing chemotherapy drug, for 6 days (Figure 1B). Miltefosine treatment efficiently reduced the growth of intestinal tumour organoids even at a lower concentration (1 μM), while it showed no cytotoxic effect against normal intestinal organoids even at a higher concentration (10 μM). On the other hand, oxaliplatin had a similar and potent inhibitory effect against the growth of both normal and tumour organoids (Figure 1B). Next, we compared the growth inhibitory effects of miltefosine in a panel of human CRC cells and normal counterparts in parallel with FACS analysis for LRs. As a result, all of the tested CRC cells (SW48, HCT116, HCT15, DLD1, HT29, SW480, hCRC1, hCRC2, hCRC4, hCRC4) contained higher LR levels than their normal counterparts (CCD‐18Co and FHC, Figure 1C). In summary, miltefosine showed a higher inhibitory effect on cellular growth against all tested CRC cells but only a minimal effect in normal colon cell lines (Figure 1C). Previously, several reports from other research groups found that cancer cells with a higher level of LRs showed greater sensitivity to the LR‐disrupting reagent methyl‐β‐cyclodextrin (MβCD) in epidermoid, prostate, breast and urinary bladder cancer.^49^, ^54^ Together with previous reports, our findings indicate that the elevated LRs in CRC cells are correlated with the preferential cytotoxic effect of the LR‐disrupting drug miltefosine.

Inhibition of cancer growth is potentially caused by various factors, including increased apoptosis, abnormal cell cycle regulation and decreased cell survival. Thus, we further estimated the effects of miltefosine on these processes. Treatment with miltefosine induced an increase in apoptotic signalling (Figure 1D) in a dose‐dependent manner (Figure 1E), as indicated by the levels of cleaved PARP, caspase‐3 and Annexin V. Furthermore, alterations in the cell cycle, such as an increase in the number of cells in subG1/G0 phase, accumulation of cells in S phase and a reduction in the number of cells in the G2/M phase, were observed after the miltefosine treatment (Figure 1F). Moreover, miltefosine treatment reduced anchorage‐independent growth (AIG) in CRC cells (Figure 1G). Importantly, these anticancer effects of miltefosine were accompanied by a reduction in LR abundance on cancer cell membranes in a dose‐dependent manner (Supplementary Figure S1E). In addition, LR‐disrupting drugs, which reduced the LR abundance on the cancer cell membrane (Supplementary Figure S1F), significantly retarded the growth of cancer cells (Supplementary Figure S1F, G). Collectively, these data suggest that miltefosine exerts a potent inhibitory effect on CRC cells through the comprehensive regulation of apoptosis induction, cell cycle interruption and cell survival attenuation. Next, we examined the potential of miltefosine in combination therapy for CRC treatment based on clonogenic survival after radiation (RT)^55^ and IC_50_ ^56^ against 5‐fluorouracil and oxaliplatin. The results showed that co‐treatment with miltefosine significantly reduced the survival fraction of CRC cells after RT exposure, conferring improved therapeutic efficacy against RT (Figure 1H). Similarly, co‐treatment with miltefosine significantly sensitized CRC cells to conventional chemotherapeutic agents, such as 5‐fluorouracil and oxaliplatin, which is indicated by lower IC_50_ values (Supplementary Figures S1H and S1I). Taken together, these results demonstrate the potent efficacy of miltefosine in CRC cells as both a single and combination agent.

### 3.2 Lipid raft disruption by miltefosine reduces the cancer stem‐like cell population

**FIGURE 2:**
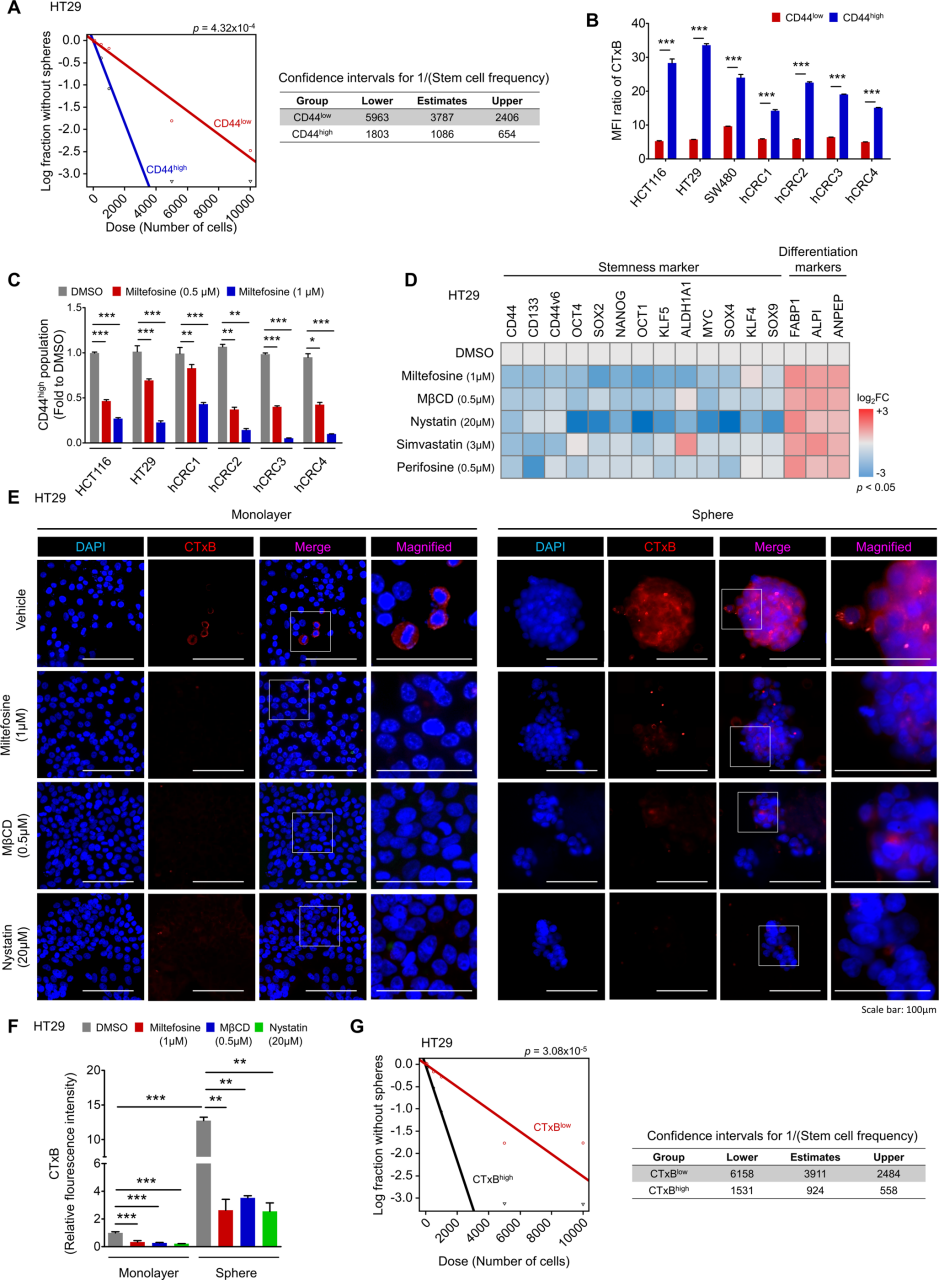
Disruption of LRs by miltefosine halted cancer stemness. (A) Comparison of sphere‐forming ability between the CD44^high^ and CD44^low^ subpopulations based on in vitro limiting dilution assay. Sorted CD44^high^ and CD44^low^ cells were plated at different cell number (10, 100, 500, 1000, 5000 and 10,000 cells/well, *n* = 12/group). After 14 days, the number of wells without spheres was counted. And the stem cell frequency and statistical value were analysed by a webtool (http://bioinf.wehi.edu.au/software/elda). (B) Comparison of LR levels in CD44^high^ and CD44^low^ populations. Cells were doubly stained with CTxB and CD44 and analysed by FACS. The bar graph shows the median fluorescence intensity (MFI) ratio of CTxB within CD44^low^ or CD44^high^ cells (mean ± SEM, *n* = 5/group). The MFI value of the CTxB‐stained sample was normalized with the MFI of IsoAb. Values were statistically analysed using Student's *t*‐test. (C) Reduction of CD44^high^ population after miltefosine treatment in various CRC cells. Cells were treated with DMSO, 0.5 or 1 μM miltefosine for 48 h. The bar graph shows the relative amount of CD44^high^ population as fold to DMSO‐treated group (mean ± SEM, *n* = 3/group). (D) Altered gene expressions of stem‐related markers in HT29 cells after 48‐h treatment of LR‐disrupting chemicals. Gene expression levels were determined by RT‐qPCR. The heatmap shows the relative mRNA levels of indicated genes. The colours of the heatmap represent the value of log_2_‐fold change (log_2_ FC) in the range of –3 to +3 with *p* < .05. (E), (F) Representative immunofluorescent images (E) and corresponding quantitative analysis (F) of altered LR levels after 48‐h treatment of miltefosine or other LR‐disrupting drugs such as MβCD and nystatin. The LR levels were compared between monolayer cultured cells and sphere cultured cells. Immunostaining for DAPI (blue) and CTxB (red) with merged and magnified images. Relative fluorescent intensity of CTxB was quantified by using Image pro premier 9 (Media Cybermetics) with normalization to DAPI intensity. Quantification was performed in three randomly selected fields for each specimen (*n* = 3/group). (C, D, F) Statistical values were analysed by one‐way ANOVA with Dunnett's multiple comparison test. (G) Comparison of sphere‐forming ability between the CTxB^high^ and CTxB^low^ subpopulations. In vitro limiting dilution assay and statistical calculation were performed as described in Figure 2A. *, ** and *** indicate *p* < .05, *p* < .01 and *p* < .001, respectively

Current therapeutic strategies against cancer have severe limitations that frequently lead to treatment failure. Resistance to conventional treatment, recurrence after treatment and metastasis are common causes of treatment failure in multiple malignancies.^57^ Of note, CSCs, a subpopulation of cells within tumours considered “the seed” of cancer, are thought to help impart resistance to conventional anticancer therapy and to increase the risk of metastasis and recurrence.^58^ Since we observed that miltefosine‐sensitized CRC cells to conventional therapy (Figures 1H and Supplementary Figure S1E and S1F), we investigated whether miltefosine could specifically target CSCs as a novel therapeutic agent for CRC treatment. CD44 has been widely used as a putative CSC marker in a wide range of cancer types including CRC because the CD44^high^ population exhibits distinct CSC phenotypes such as the ability to initiate tumourigenesis after low‐density translation into immune‐deficient mice, the ability to recapitulate the original tumour heterogeneity and the ability to grow as floating spheres.^59^ Consistent with previous reports, the sorted CD44^high^ cells showed a significantly higher ability to form spheres, as indicated by the increased CSC frequency (1/1086), than their CD44^low^ counterparts (1/3787, Figures 2A and Supplementary Figure S2A). Then, we compared the LR levels between CD44^high^ and CD44^low^ populations by using multiple CRC cells and observed that CD44^high^ cells harboured a higher fraction of CRC cells with higher LR levels (CTxB^high^) than the CD44^low^ population (Figure 2B and Supplementary Figure S2B), indicating the significant enrichment of LRs in CD44^high^ cells. To determine whether this finding would be applicable to other CSC markers, we examined the relevance of LR levels to other colorectal CSC markers such as CD133^60^ and ALDH1.^61^, ^62^ The data revealed that LR levels were significantly enriched in the CD133^high^ and ALDH1^high^ populations compared with their negative counterparts (Supplementary Figure S2C). In parallel, miltefosine treatment significantly reduced the proportion of the CD44^high^ population in multiple CRC cells in a dose‐dependent manner (Figures 2C and Supplementary Figure S2D). Interestingly, when we isolated CD44^high^ and CD44^low^ populations from HT29 cells and visualized live/or dead cells after miltefosine treatment, we observed a greater induction of cell death in the CD44^high^ population than in the CD44^low^ population (Supplementary Figure S2E). Consistently, the CD44^high^ population isolated from patient‐derived CRC cells (hCRC2) also showed significantly increased apoptotic cells upon miltefosine treatment compared with the CD44^low^ population (Supplementary Figure S2F and S2G). These results suggest that the enhanced miltefosine sensitivity of the CD44^high^ population may lead to a reduction in the CD44^high^ population. In parallel, among CD44^high^, CD44^low^ and bulk cells, CD44^high^ cells showed greater sensitivity to miltefosine, as indicated by the lowest IC_50_ values (Supplementary Figure S2H). Collectively, these results indicate that miltefosine preferentially targets CSCs as well as bulk tumour cells.

To obtain deeper insight into this phenomenon, we carried out a gene expression analysis against a set of stem‐ or differentiation‐related genes by using various LR‐disrupting reagents (Figure 2D). Both miltefosine and perifosine, APL analogs, significantly attenuated the expression of CSC markers (CD44, CD133, CD44v6, ALDH1) and stem transcription factors (OCT4, SOX2, NANOG, OCT1, KLF5, ALDH1, MYC, SOX4, KLF4, SOX9), while they augmented the expression of intestinal differentiation markers (FABP1, ALPI, ANPEP). Similarly, treatment with three other reagents, namely, methyl‐β‐cyclodextrin (MβCD) and nystatin, which disrupt LRs by chelating cholesterol and simvastatin, which lowers LRs by blocking cholesterol synthesis, altered the gene expression pattern as APL drugs did; these reagents decreased the expression of stem‐related genes and increased the expression of differentiation marker genes, suggesting that targeting LRs can attenuate the CSC phenotype in CRC cells.

Next, we applied immunofluorescence assays to visually confirm the LRs in CSCs and the LR‐disrupting effect of miltefosine. Under sphere culture conditions, CSCs can self‐renew and proliferate and thus can form sphere‐like morphologies, while non‐CSCs fail to grow and subsequently die.^63^ The gene expression panel of stemness‐ or differentiation‐related genes confirmed the enrichment of CSCs in sphere culture conditions (Supplementary Figure S2I). In spheres, the level of LRs was significantly higher than that in bulk tumour cells, and miltefosine treatment reduced the LR levels accompanied by a disruption of sphere formation (Figure 2E and F). Reductions in LR levels and destroyed spheres were repeatedly observed upon MβCD and nystatin treatment (Figure 2E and F). Consistently, FACS analysis revealed a significant reduction in LR abundance in spheres upon treatment with various LR‐disrupting reagents (Supplementary Figure S2I). This reduction in LR abundance induced by LR‐disrupting reagents led to a significant decrease in sphere growth (Supplementary Figure S2J), suggesting a correlation between LR abundance and a reduction in CSC properties. Furthermore, when we divided CRC cells into two groups according to LR levels through FACS (Supplementary Figure S2K), CTxB^high^ cells showed a greater sphere‐forming capacity, as indicated by the increased CSC frequency (1/924), than those with lower LR levels (CTxB^low^, 1/3911; Figure 2G and Supplementary Figure S2L). Collectively, these results suggest that LR disruption preferentially targets LR‐enriched CSCs.

### 3.3 Miltefosine treatment reduces the CSC phenotype and blocks cancer cell regrowth and metastasis

**FIGURE 3:**
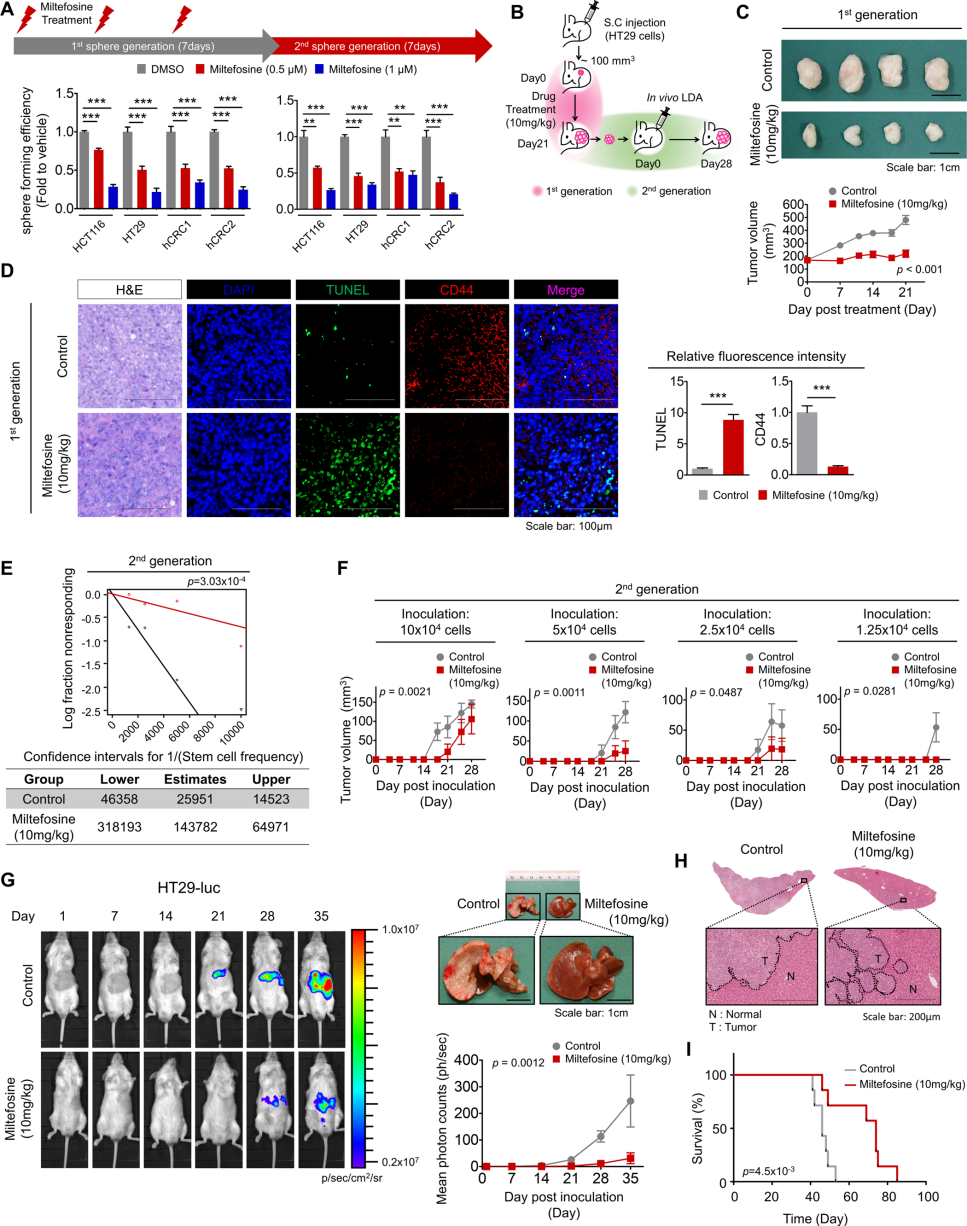
Miltefosine reduces cancer stemness in vivo. (A) Comparison of sphere‐forming efficiency and re‐growth potential of the DMSO‐ or miltefosine‐treated CRC cells. DMSO, 0.5 and 1 μM miltefosine were administered every other day throughout the first sphere generation for 7 days. And then, cells were collected, isolated into single cells and reseeded for the second sphere generation. No miltefosine treatment was applied during the second generation. The sphere‐forming efficiency was calculated as follows: the number of a sphere formed/number of plated cells. Statistical values were analysed by one‐way ANOVA with Dunnett's multiple comparison test. (B)–(F) Xenograft mouse model examining the therapeutic effect of miltefosine. (B) In vivo experimental scheme for investigating the antitumour effect of miltefosine on the first‐generation and second‐generation xenograft tumours. HT29 cells (1 × 10^6^ cells/mouse) were inoculated into NSG mice, and tumours were allowed to reach in size approximately 100 mm^3^. And then mice were treated with PBS (control) or miltefosine for 21 days (*n* = 8/group, 10 mg/kg, intraperitoneal, daily). The growth of tumours (first generation) was monitored twice a week. On the twenty‐first day, mice were sacrificed and the first generation tumours were removed for biological analyses. To examine the effect of miltefosine on tumour regrowth potential, tumour cells were isolated from the first generation tumours and reinjected into NSG mice (12,500, 25,000, 50,000 and 100,000 cells/mouse, *n* = 6/group) for in vivo LDA. No miltefosine treatment was applied during this in vivo LDA. The regrowth of tumours (second generation) was monitored for 28 days. On the 28^th^ day after reinjection, mice were sacrificed and the incidence of tumour‐bearing mice was determined. (C) Representative images (top) of first generation tumours. Graph (bottom) shows the tumour volume (mean ± SEM, *n* = 8/group). Tumour volume was monitored over time with the following formula: Tumour volume = length × width^2^/2. (D) Representative immunofluorescent images (left) and quantitative analysis (right) of xenografted tumours (first generation) comparing the control and miltefosine‐treated groups. Relative fluorescent intensity of TUNEL and CD44 was quantified by using Image pro premier 9 (Media Cybermetics) with normalization to DAPI intensity. Quantification was performed in three randomly selected fields for each specimen (*n* = 8/group). Statistical calculations were analysed by Student's *t*‐test (E) The stem cell frequency and statistical value were analysed based on the incidence of tumour‐bearing mice (second generation, *n* = 6/group) by using a webtool (http://bioinf.wehi.edu.au/ software/elda). (F) Regrowth of the second generation tumour was monitored twice a week (*n* = 6/group). (G)–(I) Effect of miltefosine on liver metastasis. (G) Representative in vivo images of liver metastasis (left), corresponding quantitative region‐of‐interest analysis (bottom right) and representative images of metastasized livers of both the control and miltefosine‐treated groups (top right). Mice were tracked for 35 days since the splenic injection of HT29‐luc. The graph shows the mean ± SEM (*n* = 9/group). (H) Representative H&E‐stained liver metastasis and magnified images of both the control and miltefosine‐treated groups. N: normal, T: Tumour. (I) Survival analysis of the control and miltefosine‐treated groups (*n* = 7/group). Survival curves were plotted using the Kaplan–Meier method, and the statistical significance was determined by the log‐rank test. (C, F, G) Statistical significance of tumour growth was determined by the two‐way repeated‐measures ANOVA followed by Bonferroni post‐tests. *, ** and *** indicate *p *< .05, *p* < .01 and *p* < .001, respectively

To investigate the effect of miltefosine on CSC properties, we conducted serial sphere‐forming assays in vitro. During primary sphere formation, treatment with miltefosine resulted in a dose‐dependent reduction in spheres (Figure 3A). Then, we isolated single cells from spheres, replated them and cultured them under sphere culture conditions to generate secondary spheres without additional miltefosine treatment and to determine whether the effect of miltefosine was reversible. Miltefosine treatment significantly and irreversibly reduced the reformation of spheres (Figure 3A). Additionally, we confirmed that the miltefosine‐induced reduction in LR abundance persisted, even when miltefosine was removed (Supplementary Figure Figure S3A). This result supports a hypothetical mechanism by which this irreversible reduction in sphere reformation might be derived from LR disruption, which was not restored even when miltefosine was removed. Next, we examined the in vivo efficacy of miltefosine as an anticancer agent in mouse xenograft models. Similar to the in vitro assay, we examined primary tumour growth upon miltefosine treatment and then isolated single tumour cells from the primary tumour burden for inoculation and regeneration of secondary tumour growth (Figure 3B). The data showed that miltefosine treatment (10 mg/kg, administered i.p.) resulted in a significant reduction in primary tumour growth (Figure 3C). Immunostaining assays revealed a reduced proportion of CD44^high^ cells along with increased apoptotic cells (TUNEL^+^) and reduced proliferative cells (Ki67^+^) (Figures 3D and Supplementary Figure S3B), while no significant changes in body weight were observed (Supplementary Figure S3C). No obvious clinical signs, including anorexia, salivation, diarrhea, vomiting, polyuria, anuria and faecal changes, were observed during miltefosine treatment. Further, the histological assessment did not show any abnormalities in either the livers or kidneys of miltefosine‐treated mice (Supplementary Figure S3D). To examine the effect of miltefosine on tumour regrowth potential, we performed an in vivo LDA during the second generation of tumour growth. From freshly digested tumour tissues, we transplanted limiting dilutions (from 100,000 to 12,500 cells) of the single‐cell preparation into mice without additional miltefosine treatment. Treatment with miltefosine significantly reduced the incidence of secondary tumour‐bearing mice (Figures 3E and Supplementary Figure S3E), resulting in a decrease in the estimated CSC frequency (1/143,782) compared to that of control mice (1/25,951). The growth curve of the secondary tumour burden showed a significant difference between miltefosine‐treated and control mice (Figure 3F), suggesting an irreversible inhibitory effect of miltefosine on tumour regrowth in a CRC xenograft model. Furthermore, we used a splenic injection model to investigate the effect of miltefosine on CRC metastasis to the liver. We used cells stably expressing firefly luciferase and monitored whole‐body bioluminescence for noninvasive detection (Figure 3G). Liver metastasis was significantly attenuated in miltefosine‐treated mice compared with control counterparts (Figure 3G and 3H). Miltefosine treatment significantly extended the survival of tumour‐bearing mice (Figure 3I). These results indicate that miltefosine treatment can effectively target CRC cells and irreversibly attenuate the CSC phenotype, thus inhibiting tumour growth, metastasis and regrowth in vivo.

### 3.4 LR disruption by miltefosine transcriptionally reduces CHEK1 expression

**FIGURE 4:**
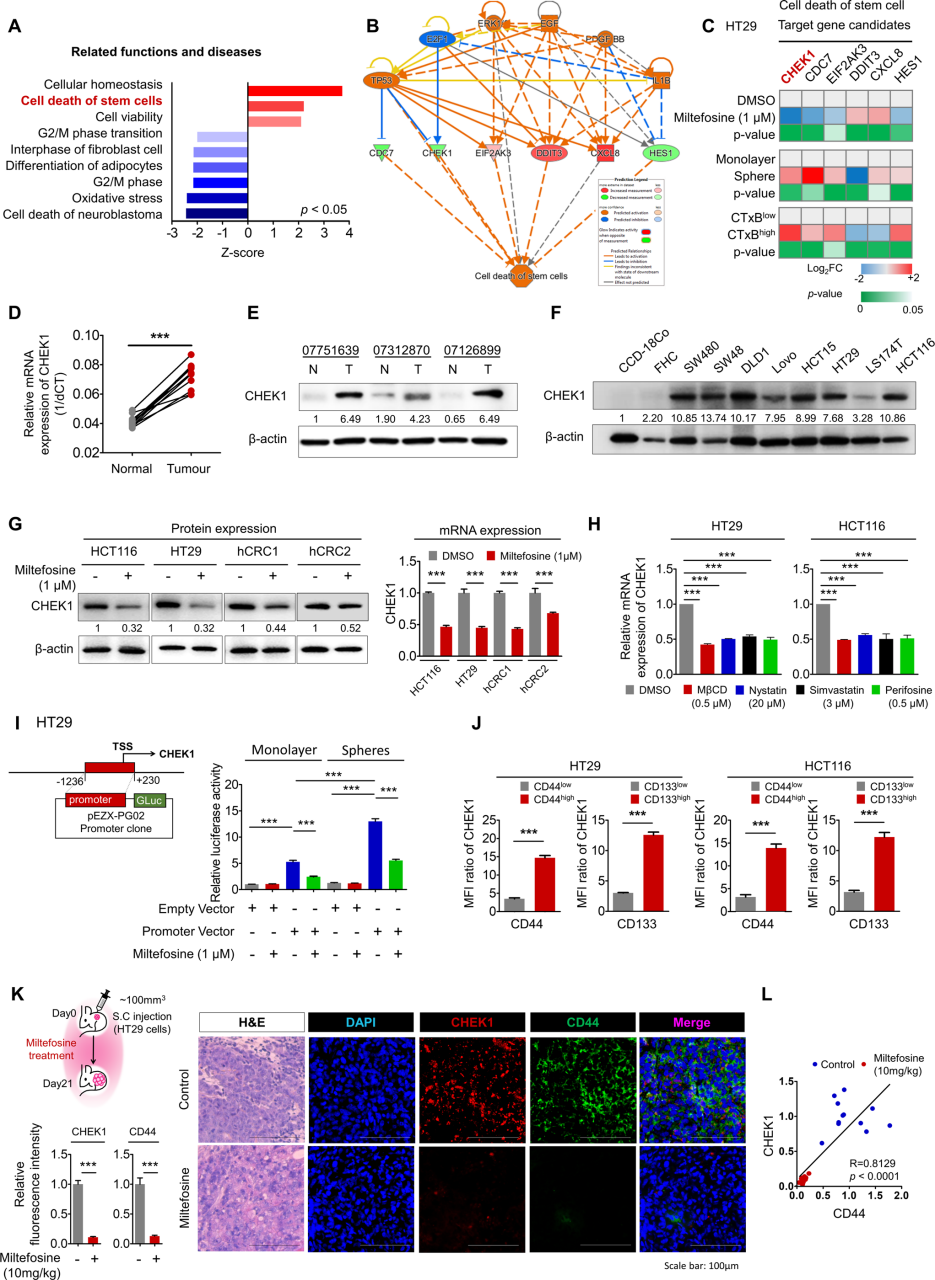
CHEK1 is a key factor of miltefosine in controlling cancer stem cell death. (A) IPA functional analysis of DEGs correlated with miltefosine. Biological functions and diseases with statistical significance (*Z*‐score > 2, *p* < .05) are presented. (B) Predicted network showing six target gene candidates and upstream regulators contributing to the increase in cell death of stem cells upon miltefosine treatment. Dotted lines are indirect interactions, and solid lines are direct interactions. Genes in red are upregulated DEGs, genes in green are downregulated DEGs, and genes in orange (activated) and blue (inhibited) are predicted by IPA. (C) RT‐qPCR validation for six target gene candidates dedicated to the cell death of stem cells. mRNA expression levels were determined by RT‐qPCR and compared under various experimental conditions: DMSO vs. miltefosine treatment (1 μM, 48 h), monolayer culture vs sphere culture and CTxB^low^ vs CTxB^high^ cells. The heatmap represents the value of log_2_ FC in the range of –2 to +2 with *p *< .05 (*n* = 3/group). (D) Relative mRNA expression (E) and protein expression of CHEK1 in patient‐derived tumour tissues and matched normal tissues (*n* = 15). Statistical significance was determined by the paired *t*‐test. (F) Protein expression of CHEK1 in normal colon epithelial cell lines (CCD‐18Co and FHC) and CRC cell lines. β‐Actin was used as a loading control. (G) Protein expression (left) and relative mRNA expression (right) of CHEK1 in CRC cells upon 48‐ h treatment of miltefosine (1 μM). β‐Actin was used as a loading control. (E)–(G) Values below each lane indicate the relative band intensity of target protein normalized to β‐actin as fold to control lane. (H) Relative mRNA expression of CHEK1 upon 48‐ h treatment of indicated chemicals. (I) The promoter activity of CHEK1 upon miltefosine treatment was tested by a reporter assay. The CHEK1 promoter region (–‐1236 to +230)‐containing vector was transfected into cells and cells were cultured in monolayer or sphere culture condition for 3 days with or without miltefosine treatment (1 μM). (J) Comparison of CHEK1 expression levels in CD44^high^ and CD44^low^ populations or CD133^high^ and CD133^low^ populations. Cells were doubly stained with CTxB and CD44/or CD133 and analysed by FACS. The bar graph shows the MFI ratio of CHEK1 within the indicated populations (MFI normalized with Iso‐Ab, mean ± SEM, *n* = 3/group). (K) Experimental scheme (top left) of HT29 xenograft mouse model. HT29 cells were inoculated into NSG mice and allowed to form tumours reaching around 100 mm^3^. And then mice were treated with PBS (control) or miltefosine (*n* = 11/group). Representative immunofluorescent images (right) and quantitative analysis (left bottom) of the xenografted tumours comparing the control and miltefosine‐treated groups. The relative fluorescent intensity of CHEK1 (red) and CD44 (green) was quantified by using Image pro premier 9 (Media Cybermetics) with normalization to DAPI (blue) intensity. Quantification was performed in three randomly selected fields for each specimen (*n* = 11/group). (L) Correlation analysis of CHEK1 and CD44 expression in the xenografted tumours of Figure 4K. Correlation between CHEK1 and CD44 expression was analysed by Spearman's correlation coefficient. (G)–(K) Bar graphs indicate mean ± SEM (*n* = 3/group). Statistical values were analysed by Student's *t*‐test (C, G, J, K) or one‐way ANOVA with Dunnett's multiple comparisons (H and I). *, ** and *** indicate *p *< .05, *p* < .01 and *p* < .001, respectively

As mentioned above, our data indicate that miltefosine efficiently targets CSCs. However, our understanding of the molecular pathways involved in this biological function has not yet been elucidated. To investigate the molecular mechanism of miltefosine, we performed a microarray and identified gene expression alterations upon miltefosine treatment (|log 2FC|≥1, *p* < .05, Supplementary Table S3). The significantly altered genes were analysed by the web‐based bioinformatics tool, Enrichr (http://amp.pharm.mssm.edu/Enrichr/), to determine the signalling pathways that could control the expression of these genes.^64^, ^65^ The screening threshold was set as adjusted *p* < .01. Enrichment analysis of 62 downregulated genes was carried out using the ARCH4 kinase Coexp,^66^ and the results showed the potential top 10 signalling kinases arranged by a combined score together with the *p*‐value and odds ratio (Supplementary Figure S4A and Supplementary Table S7). Reduction of genes upon miltefosine treatment was significantly associated with inhibition of various kinases such PLK4, MELK, CDK1, MASTL, PDK, AURKB, BUB1B, TTK, CHEK1 and BUB1. Additionally, enrichment analysis of 192 upregulated genes revealed that TRIB3 and RNASEL kinases were significantly associated with increased gene expression. These data suggest that LR disruption by miltefosine results in chaotic dynamics of multiple signalling pathways, leading to differential gene regulation. Next, we explored the biological functions associated with differentially expressed genes upon miltefosine treatment by using GSEA. The results revealed that gene clusters associated with cancer proliferation, cell cycle regulation and DNA repair were significantly downregulated upon miltefosine treatment (Supplementary Figure S4B). In parallel, we performed IPA to identify the potential biological functions that were significantly affected by miltefosine. IPA indicated that the cell death of stem cells was significantly activated upon miltefosine treatment, while G2/M regulation was significantly inhibited (Figure 4A). By visualizing the upstream regulators and downstream molecules contributing to the increase in cell death of stem cells, we identified six potential candidate genes (CDC7, CHEK1, EIF2AK3, DDIT3, CXCL8 and HES1; Figure 4B). To verify the informatics results, we conducted a series of RT‐qPCR validations on the changes in the expression of six candidate genes under various experimental conditions (miltefosine treatment, CSC‐enriched spheres, CSC‐enriched CTxB^high^ cells). The results showed that CHEK1 was potently reduced by miltefosine treatment, and, concomitantly, it was strongly expressed in the CSC‐enriched spheres and CTxB^high^ cells (Figure 4C). Consistently, when we performed dual staining for CHEK1 and CTxB, we observed that CHEK1 expression was increased in CTxB^high^ cells compared with CTxB^low^ cells (Supplementary Figure S4C). Moreover, when we compared the LR levels and CHEK1 expression levels in monolayer and CSC‐enriched spheres, both the first and second generation spheres expressed significantly elevated CHEK1 protein levels compared with monolayer cells, and miltefosine treatment significantly reduced both LR levels and CHEK1 expression under monolayer and sphere culture conditions (Supplementary Figure S4D). Of note, miltefosine‐induced reductions in LR levels and CHEK1 proteins appeared in a dose‐dependent manner (Supplementary Figure S4E). Collectively, according to these sets of multiple validations together with bioinformatics predictions, we selected CHEK1 and further investigated it as a candidate gene that may mediate CSC death upon LR disruption by miltefosine treatment.

To validate the widely appreciated notion that CHEK1 is a potential therapeutic target for CRC treatment,^11^ we carried out a series of analyses by using CRC patient tissues and a panel of CRC cells. Using patient‐derived tissues, we confirmed that both the mRNA and protein levels of CHEK1 were significantly enhanced in CRC tissues compared to matched normal tissues (Figures 4D,E and Supplementary Figure S4F). Gene expression analysis achieved from the Oncomine clinical database (www.oncomine.org) further supported the increase in CHEK1 expression in CRC tissues compared with normal counterparts (GSE35834; Supplementary Figure S4C). Moreover, higher grades of CRC showed greater increases in CHEK1 expression (Supplementary Figure S4G and S4H), and CRC patients with higher CHEK1 expression showed significantly poorer recurrence‐free survival than patients with lower CHEK1 expression (*p*‐value = .012; Supplementary Figure S4I). In accordance, the protein level of CHEK1 was also significantly elevated in a panel of CRC cells compared with normal colon cell lines (Figure 4F).

Next, we examined the effect of miltefosine on CHEK1 expression in various CRC cells. Miltefosine significantly reduced the expression of CHEK1 at both the mRNA and protein levels (Figure 4G). A similar reduction in CHEK1 expression was shown upon treatment with LR‐disrupting agents (MβCD, nystatin, simvastatin and perifosine; Figure 4H). As the mRNA level of CHEK1 was altered by miltefosine, we then investigated whether miltefosine treatment regulates the transcription of CHEK1 by suppressing CHEK1 promoter activity. A reporter assay showed a reduction in CHEK1 promoter activity upon miltefosine treatment, thus confirming the inhibitory role of miltefosine in CHEK1 transcription (Figure 4I). Interestingly, CSC‐enriched spheres exhibited significantly upregulated CHEK1 promoter activity compared with monolayer bulk cells (Figure 4I). Of note, the extent of reduction was significantly greater in spheres than in bulk cells, suggesting that LR disruption may affect CHEK1 promoter activity more preferentially in LR‐enriched CSCs (Figure 4I).

Then, we examined the CHEK1 expression levels in the CSC‐enriched fraction defined by CSC markers, such as CD44, CD133 and ALDH1. FACS analysis showed that CHEK1 expression was significantly elevated in the CSC populations in both HT29 and HCT116 cell lines (Figure 4J and Supplementary Figure S4J‐L). Furthermore, histological analyses of primary tumours obtained from CRC xenograft mice revealed that miltefosine treatment reduced the expression of both CHEK1 and CD44 (Figure 4K). Further correlation analysis confirmed a statistically significant positive correlation between CHEK1 and CD44 expression (Figure 4K, L). Collectively, these results suggest that miltefosine can potently target CHEK1 expression, which is elevated in CRC and further enriched in the CSC population.

### 3.5 Targeting CHEK1 reduces CSC properties independent of P53 status

**FIGURE 5:**
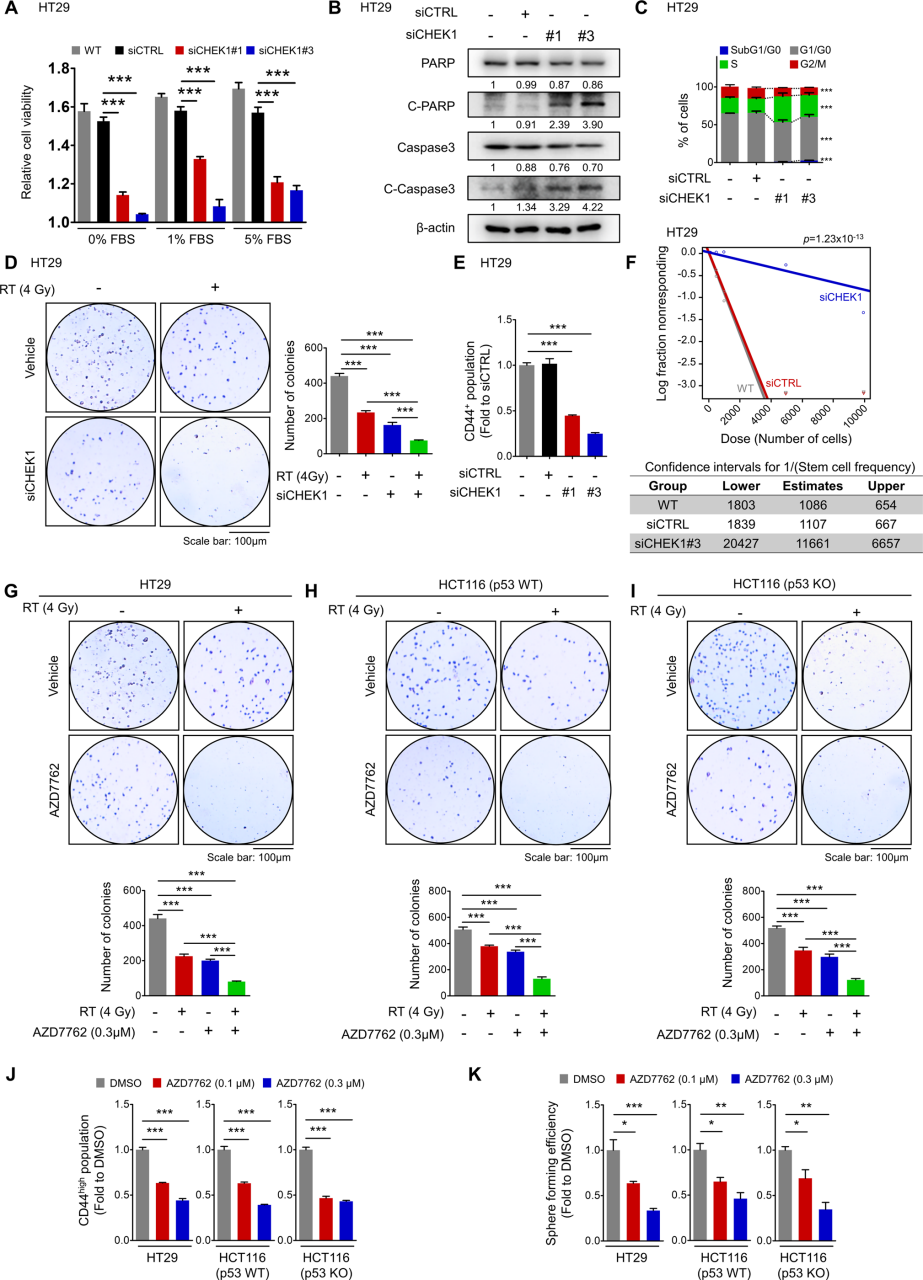
Inhibiting CHEK1 efficiently reduces cancer stemness independent of P53 status. (A) Cell growth upon siRNA transfection was compared in various serum conditions (0, 1, 5% FBS). After siRNA transfection, cells were seeded on 96‐well plates and the cell viability was measured by MTT assays right after cell attachment (after 6 h) or after 48‐ h incubation. The bar graph indicates the fold increase in cell viability during this 48‐ h period (*n* = 3/group). (B) Protein expressions of apoptotic signalling components were determined at 48 h after CHEK1 knockdown. Values below each lane indicate the relative band intensity of target protein normalized to β‐actin as fold to control lane. (C) Cell cycle analysis at 48 h after siRNA transfection. The cell subpopulations represented are subG1/G0 (blue), G1/G phase (gray), S phase (green) and G2/M phase (red). (D) Reduction of AIG potential of CRC cells upon CHEK1 knockdown. At 48 h after siRNA transfection, cells were irradiated and seeded on 12‐well plates mixed with agar for the AIG assay. After 2 weeks, the number of colonies growing in agar was counted. (E) Reduction of CD44^high^ population upon CHEK1 knockdown. The amount of CD44^high^ population was determined by FACS at 48 h of siRNA transfection and presented as fold to control group. (F) Comparison of sphere‐forming ability between the siCTRL and siCHEK1‐transfected cells based on in vitro limiting dilution assay. After siRNA transfection, cells were plated at different cell number (10, 100, 500, 1000, 5000 and 10,000 cells/well, *n* = 12/group). After 14 days, the number of wells without spheres was counted. And the stem cell frequency and statistical value were analysed by a webtool (http://bioinf.wehi.edu.au/ software/elda). (G)–(I), of AIG potential by treatment of a CHEK1 inhibitor, AZD7762, in HT29 (G), p53 WT HCT116 (H) and p53 KO HCT116 (I) cells, which have different p53 status. Cells were treated with AZD7762 for 48 h and applied to AIG assays as described in Figure D. (J) Reduction of CD44^high^ cells upon CHEK1 inhibition. The amount of CD44^high^ population was determined by FACS at 48 h after drug treatment and presented as fold to the DMSO group. (K) Reduction of sphere‐forming efficiency by CHEK1 inhibition. Cells were treated with AZD7762 for 48 h and applied to sphere‐forming assay. The sphere‐forming efficiency was calculated as follows: the number of spheres formed/number of plated cells. (A, C–E, G–K) Graphs indicate mean ± SEM (*n* = 3/group). Statistical analyses were performed by one‐way ANOVA, with Dunnett's multiple comparison. *, ** and *** indicate *p *< .05, *p* < .01 and *p* < .001, respectively

To examine the CHEK1‐targeting effect in CRC cells, we examined the effect of CHEK1 knockdown on various cell functions, including cell proliferation, apoptosis, cell cycle, survival and CSC properties. In advance, we tested the knockdown efficacy of three sequences of siRNAs against CHEK1 (siCHEK; Supplementary Figure S5A) and chose the two most efficient sequences (sequences #1 and #3) for further examination. Protein analyses confirmed the reduction in CHEK1 protein levels after transfection with siRNAs (Supplementary Figure S5B), and the maximum efficacy of siCHEK persisted at least until 96 h after transfection (Supplementary Figure S5C). In proliferation assays, a reduction in CHEK1 expression retarded CRC cell growth (Figure 5A) and concomitantly activated apoptotic signalling, as indicated by increased cleavage of PARP and Caspase 3 (Figure 5B). Additionally, as miltefosine did (Figure 1F), CHEK1 knockdown induced cell cycle interruption, such as subG1/G0 increase, S phase accumulation and G2/M phase reduction (Figure 5C and Supplementary Figure S5D). Furthermore, CHEK1 reduction attenuated the anchorage‐independent growth potential of CRC cells and enhanced the RT‐induced reduction in AIG potential (Figure 5D). As miltefosine efficiently reduced CSC properties (Figures 2 and 3), CHEK1 reduction decreased the CD44^high^ cells (Figures 5E). Consistent with CD44^high^ cells harbouring elevated LR levels (Figure 2B), CHEK1 knockdown resulted in a reduction in the CTxB^high^ population (Supplementary Figure S5E). Consistently, the transcriptional comparison suggested a global trend of CSC reduction by CHEK1 knockdown by showing reductions in the expression of CSC markers and stem cell‐related transcription factors ( Supplementary Figure S5F). Moreover, the sphere‐forming potential of CRC cells was significantly attenuated by CHEK1 knockdown, as indicated by reduced CSC frequencies; 1/11661 in CHEK1 knockdown cells and 1/1068 and 1/1107 in WT and siCTRL cells, respectively (Figure 5F). These data suggest that targeting CHEK1 exhibits a potent anticancer effect in CRC cells by attenuating cell growth, survival and stemness and promoting apoptosis and cell cycle alteration, conferring potential as a therapeutic target.

Next, we generated a CHEK1‐overexpressing cell line (Supplementary Figure S5G) to investigate whether CHEK1 reduction was essential for the therapeutic effect of miltefosine. Interestingly, the inhibitory effects of miltefosine on cancer cell viability, sphere formation and stem‐related TF expression were significantly blocked by CHEK1 overexpression (Supplementary Figure S5H–J). Collectively, these results suggest that the CHEK1 reduction mediates, at least in part, the therapeutic effect of miltefosine.

Several studies from other research groups have identified P53 mutation as a crucial determinant of the effectiveness of CHEK1 inhibition alone or in combination with conventional therapy, which is logical, as the dual loss of CHEK1 and P53 would result in the abrogation of all checkpoints and further potentiate sensitivity to chemotherapy.^7^, ^67^, ^68^ However, in marked contrast, the CHEK1 inhibitors SCH900776 and MU380 effectively sensitized both P53 knockout (KO) and WT prostate cancer cells to gemcitabine.^69^ No significant correlation was observed between CHEK1 inhibitor efficacy and the status of P53 in a panel of multiple types of cancer cell lines.^70^ Similarly, the impairment of CHEK1 by UCN‐01 or siRNA caused similar sensitization of paired U2OS cells with WT P53 or P53 knockdown to irinotecan and cisplatin.^67^ AZD7762 is a newer generation, more selective CHEK1 inhibitor.^71^ AZD7762 inhibits CHEK1 by reversibly binding to the ATP‐binding site of CHEK1.^71^ Therefore, to examine the CHEK1‐targeting effects in CRC cells in terms of P53 status, we carried out an in‐depth analysis using AZD7762 in a panel of P53 cell lines composed of the P53 mutant HT29 cell line, P53 WT HCT116 cell line and P53 KO HCT116 cell line (Supplementary Figure S5K). First, we compared the sensitivity to AZD7762 by estimating the IC_50_ values based on cell viability. There was no significant difference among the IC_50_ values of all tested cells; HT29: 0.54 μM, P53 WT HCT116: 0.68 μM, P53 KO HCT116: 0.78 μM; Supplementary Figure S5L). The reduction in cell viability by CHEK1 inhibition was accompanied by an increase in apoptosis (Supplementary Figure S5M). Next, we compared the effect of AZD7762 treatment on AIG potential and CSC properties. As a result, AZD7762 showed a similar and potent effect on reductions in AIG potentials in both HT29 and HCT116 cells (Figure 5G, H). Concomitantly, treatment with AZD7762 enhanced the reductions in AIG potential after RT exposure in both P53 WT HCT116 and mutant HT29 cells at similar potency (Figure 5G, H). Furthermore, we compared the effect of AZD7762 in P53 WT and KO HCT116 cells (Figure 5H, I). Then, we repeatedly confirmed that a reduction in AIG potential by CHEK1 inhibitor treatment, and the radiosensitizing effect of a CHEK1 inhibitor was evident in both P53 WT and KO cell lines. Next, AZD7762 showed a potent effect on the reduction in the CD44^high^ population in all three cell lines, P53 mutant, WT and KO (Figure 5J and Supplementary Figure S5N). Similarly, AZD7762 treatment repeatedly decreased the sphere‐forming potential in all three cell lines with similar potency (Figure 5K). These data indicate that targeting CHEK1 with AZD7762 exhibits a CSC‐targeting effect and may not be solely dependent on P53 status. Therefore, our data together with previous reports from other groups suggest that it is likely that the differences in the sensitivity of individual cell lines against CHEK1 inhibition could be explained by a loss of G1 control in general, rather than by p53 status alone; thus, CHEK1 inhibition by miltefosine can efficiently target both P53 mutant and WT CRC cells.

### 3.6 CHEK1 inhibition by miltefosine induces CSC death through mitotic catastrophe

**FIGURE 6:**
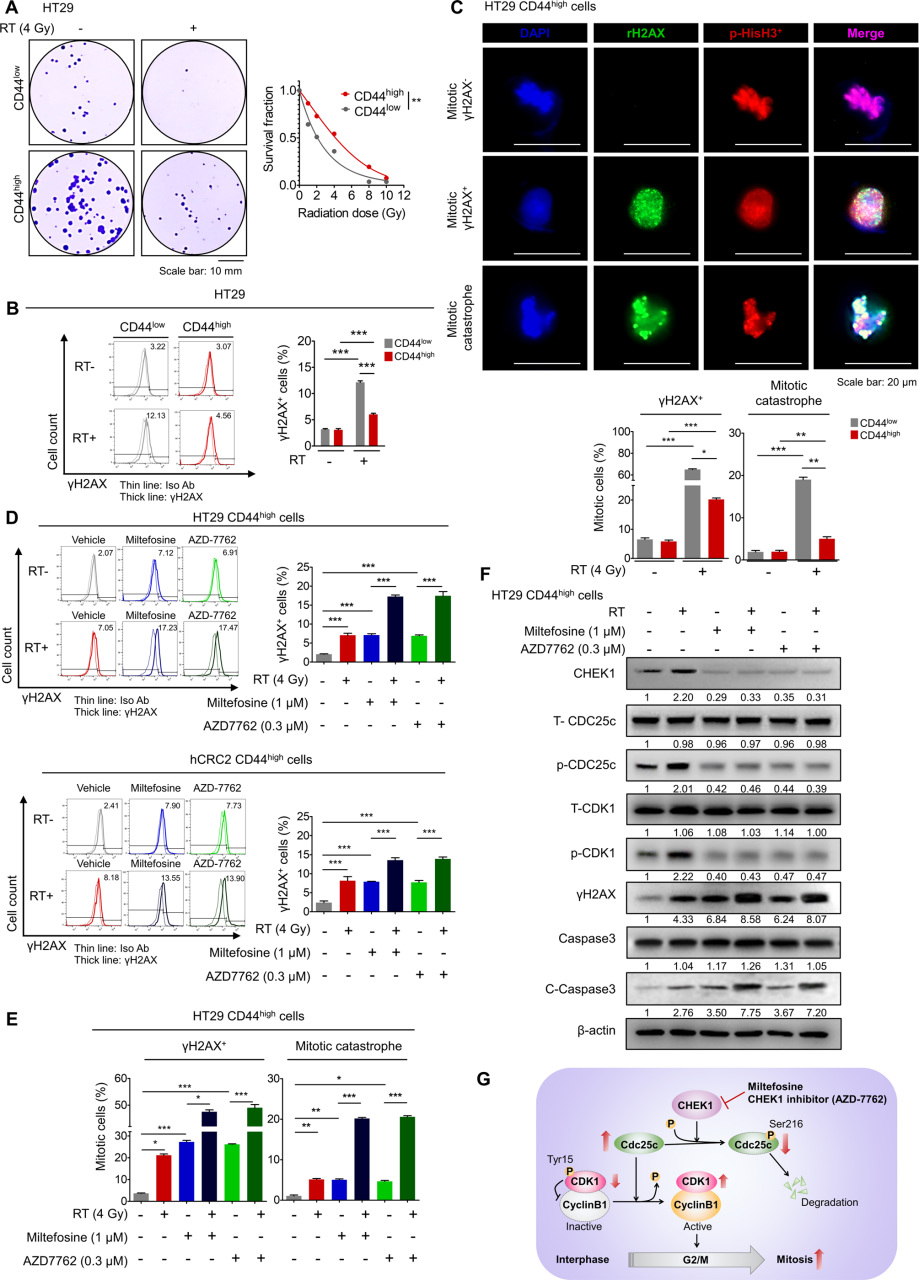
CHEK1 inhibition causes a mitotic catastrophe, consequently inducing CSC death. (A) Comparison of relative sensitivity to RT between CD44^low^ and CD44^high^ populations. Cells were exposed to an escalating dose of radiation. Right after RT exposure, clonogenic assays were performed (*n* = 3/group). The representative images (left) of surviving colonies. The surviving fraction was calculated as the number of colonies/(number of cells seeded × PE). The platting efficiency PE was calculated using the number of colonies/number of cells seeded in the non‐irradiated cells. The generation of cell survival curves (bottom) and statistical analysis were conducted based on a linear‐quadratic model using GraphPad Prism v7.05 software (GraphPad). (B) Comparison of γH2AX levels between CD44^low^ and CD44^high^ populations after RT exposure. Representative histograms (left) show the relative γH2AX levels, and the bar graph (right) shows the percentage of the γH2AX^+^ population. The FACS gates were established by staining with an isotype antibody (Iso Ab). Thin lines indicate Iso Ab‐stained samples and thick lines indicate γH2AX‐stained samples. The bar graph indicates mean ± SEM (*n* = 3/group). (C) Representative immunofluorescence images and quantitative analysis of *p*‐HisH3^+^ mitotic cells (red) with no γH2AX (top) or high γH2AX (mitotic γH2AX^+^, middle, bottom) after RT exposure. Images of cells undergoing mitotic catastrophe as marked by nuclear blebbing and high γH2AX (mitotic catastrophe, bottom). DAPI nuclear staining is shown in blue (left) and the respective merged images (right). Separate populations of mitotic γH2AX^+^ and mitotic catastrophe were quantified as a percentage of cells among the *p*‐HisH3^+^ mitotic cells. The quantifications were performed in three randomly selected fields for each specimen from a total of six independent experiments. The bar graph indicates mean ± SEM (*n* = 6/group). (D) The γH2AX levels were analysed by FACS in DMSO‐, miltefosine‐, or AZD7762‐treated (48 h) CD44^high^ isolated from HT29 (top) or patient‐derived CRC cells (hCRC2, bottom) after RT exposure. Representative histograms (left) show the relative γH2AX levels and the bar graph shows the percentage of the γH2AX^+^ population. The FACS gates were established by staining with an isotype antibody (Iso Ab). Thin lines indicate Iso Ab‐stained samples and thick lines indicate γH2AX‐stained samples. The bar graph indicates mean ± SEM (*n* = 3/group). (E) Quantitative analysis of mitotic catastrophe in miltefosine‐ or AZD7762‐treated (48 h) HT29 CD44^high^ cells. The bar graph shows the percentage of mitotic γH2AX^+^ cells and mitotic catastrophe among the *p*‐HisH3^+^ mitotic cells. The quantifications were performed in three randomly selected fields for each specimen from a total of six independent experiments. The bar graph indicates mean ± SEM (*n* = 6/group). (F) Representative Western blots of cell cycle‐regulating factors and apoptotic signalling factors after a combination of radiation exposure and 48‐ h treatment of miltefosine/AZD7762. Values below each lane indicate the relative band intensity of target protein normalized to β‐actin as fold to control lane. (G) Proposed model of CHEK1‐dependent CSC death via mitotic catastrophe. Statistical analyses were performed by the one‐way ANOVA with Dunnett's multiple comparison. *, ** and *** indicate *p* < .05, *p* < 0.01 and *p* < 0.001, respectively

CHEK1 is an evolutionarily conserved serine/threonine kinase that regulates the cell cycle^9^. CHEK1 inhibition is known to increase replication stress in cancer cells.^11^ Thus, we examined whether miltefosine treatment induces replication stress by analysing the extent of the phosphorylated form of H2AX (γH2AX), a sensitive indicator of DNA damage and DNA replication stress.^72^ Consequently, we observed an increased extent of γH2AX in bulk cells by miltefosine treatment (Supplementary Figure S6A). Of note, the CD44^high^ population showed a more enhanced increase in γH2AX than bulk cells upon miltefosine treatment (Supplementary Figure S6A), corroborating the greater induction of cell death by miltefosine in CD44^high^ CSCs than in the CD44^low^ population (Figure 2D). CHEK1 is known to particularly regulate the initiation of a G_2_/M checkpoint in response to genotoxic stress, such as conventional radiation and chemotherapy.^9^ Therefore, the depletion or inhibition of CHEK1 has been reported to induce chromosome misalignment, lagging chromosomes and cytokinesis failure, which consequently leads to DNA damage, premature mitosis promotion and mitotic catastrophe.^11^ Mitotic catastrophe is a form of cell death associated with aberrant mitosis. It is caused by uncoordinated or improper mitotic phase progression.^5^ It is considered the primary cell death mechanism following exposure to genotoxic stresses. Therefore, we examined the effect of CHEK1 inhibition on mitotic catastrophe induction specifically in CSCs, which are known to be the source of the genotoxic stress‐resistant subpopulation. To confirm the resistance of CSCs against genotoxic stress, we isolated CD44^high^ cells and analysed the effect of their resistance phenotype against RT in various aspects. Upon RT exposure, the CD44^high^ population showed a higher survival rate than the CD44^low^ population (Figure 6A) with less accumulation of DNA damage, as indicated by the decrease in γH2AX (Figure 6B). Further neutral comet assays revealed that CD44^high^ cells harboured a lower extent of DNA damage accumulation than CD44^low^ cells after RT exposure (Supplementary Figure S6B). To endure DNA damage, cells can transiently stop cell cycle progression for DNA damage repair and then begin to undergo mitosis. However, when the cell cycle regulation mechanism is insufficient to address DNA damage, cells undergo mitosis bearing intolerable DNA damage and then finally face tragic cell death.^6^ Thus, we carried out immunofluorescence analyses to visualize the cells during mitotic catastrophe as described in a previous report from another research group.^73^ A distinct mitotic cell population expressing the phosphorylated form of histone H3 (*p*‐HisH3^+^) with intense nuclear γH2AX staining (mitotic γH2AX, middle row, Figure 6C) is likely to represent cells in the initial stages of mitotic catastrophe. A separate population of mitotic cells characterized by the loss of membrane integrity and fragmented morphology with one or more micronuclei is likely to represent cells facing tragic cell death (mitotic catastrophe, bottom row, Figure 6C). After RT exposure, we observed a relatively higher proportion of mitotic γH2AX^+^ and mitotic catastrophe in the CD44^low^ population than in the CD44^high^ population, indicating the resistance phenotype of CD44^high^ cells against genotoxic stress (Figure 6C). Collectively, our data indicate that CD44^high^ cells are more resistant to genotoxic stress, preventing mitotic catastrophe; thus, releasing the cell cycle checkpoint of CSCs is an important strategy to sensitize and eliminate CSCs under genotoxic stress.

Since we provided evidence of miltefosine‐sensitizing CRC cells against genotoxic stress, such as RT and chemotherapy (Figures 1H and Supplementary Figures S1E and S1F), we examined whether miltefosine and CHEK1 inhibitors can sensitize CSCs against genotoxic stress and stimulate CSCs to enter mitotic catastrophe. For this purpose, we isolated CD44^high^ cells and measured cellular DNA damage levels by measuring the γH2AX levels of cells upon miltefosine, AZD7762, RT and combination treatment. Individual treatment alone caused similar DNA damage levels; moreover, combination treatment with RT and miltefosine or RT and CHEK1 inhibitor enhanced DNA damage levels (Figure 6D). Immunofluorescence assays analysing the percentage of mitotic cells further confirmed the increased proportion of mitotic γH2AX^+^ and mitotic catastrophe upon individual treatment and the further increase upon combination treatment (Figure 6E). Similarly, miltefosine and AZD7762 treatment also sensitized CSCs to oxaliplatin, which was indicated by increases in mitotic γH2AX^+^ and mitotic catastrophe cellular proportions (Supplementary Figure S6C). CHEK1 is a serine/threonine kinase that is capable of guarding the improper entrance of mitosis by phosphorylating cell division cycle 25C (CDC25c), which consequently causes the degradation of CDC25c.^9^ In the presence of active CDC25c, the inactive cyclin‐dependent kinase 1 (CDK1)‐cyclin B1 complex is dephosphorylated and subsequently enables cells to enter the G2/M phase, allowing mitosis to occur. Our Western blot analyses (Figure 6F and Supplementary Figure S6D) revealed that upon RT exposure, CD44^high^ CSCs showed significant increases in phosphorylated CDC25c and CDK1 (*p*‐CDC25c and *p*‐CDK1), indicating the activation of the G2/M checkpoint, which prevents DNA damage‐bearing cells from entering mitosis (Figure 6F). However, CHEK1 inhibition by treatment with miltefosine or AZD7762 attenuated the phosphorylation of CDC25c and CDK1 even after RT, indicating the release of the CHEK1 checkpoint and continuous entry of cells into the mitotic phase even in the presence of accumulated DNA damage. Therefore, miltefosine and AZD7762 treatment resulted in a further increase in DNA damage and cell death, as confirmed by increases in γH2AX and caspase‐3 cleavage, respectively (Figure 6F and Supplementary Figure S6D). Taken together, our results suggest that CHEK1 inhibition by miltefosine treatment has strong potential to overcome the resistant phenotype of CSCs by interrupting the CHEK1‐mediated cell cycle checkpoint and inducing substantial mitotic defects (Figure 6G).

## 4 DISCUSSION

Miltefosine, a prototype APL drug, is the first drug approved for treating metastatic breast cancer on the skin.^25^ Extensive studies have reported that various APL analogs, including miltefosine, perifosine and erufosine, exhibit potent anticancer activity against multiple types of cancer, including glioblastoma and pancreatic, hepatocellular, renal and breast cancer.^25^ However, despite the profound cytotoxic effects of APLs against cancer cells, their precise mechanism of action remains to be elucidated. Here, we newly demonstrated that LR‐disrupting miltefosine preferentially targets CRC cells containing higher LRs than normal cells (Figure 1A–C). Of note, CSCs have large amounts of LRs on their membranes (Figure 2B, E), and LR disruption by miltefosine greatly reduced the proportion of the CSC population (Figure 2C–F), accompanied by a decrease in CSC properties, such as self‐renewal, tumour regrowth and metastatic potential (Figure 3). Our bioinformatics analysis predicted the possibility that multiple kinases, including CHEK1, might be affected by miltefosine treatment, resulting in chaotic dynamics of multiple signalling pathways (Supplementary Figure S4A). Among these kinases, our data support a mechanism by which reduced CHEK1 levels mediate the therapeutic effect of miltefosine by altering the G2/M cell cycle regulation, DNA damage and death of CSCs (Figures 4 and 5), as predicted by GSEA and IPA (Supplementary Figure S4B and Figure 4A,B). Although the clinical application of miltefosine is limited because of dose‐limiting side effects,^37^ more promising analogs related to miltefosine have been developed with improved efficacy and toxicological profile. In this context, our discovery of the LR/CHEK1 axis as a novel mechanism of miltefosine underlies its preferential cytotoxicity on cancer cells and will help convince the rationale for the further development of APL drugs for cancer treatment.

This study demonstrated that CHEK1 inhibition can be achieved by miltefosine. CHEK1 expression and its transcriptional activity were significantly reduced by treatment with LR‐targeting drugs including miltefosine, and the CHEK1 reduction mediated, at least in part, the therapeutic effect of miltefosine (Figure 4). Thus, the therapeutic effect of miltefosine may vary in patients depending on LR levels and CHEK1 status such as phosphorylation, kinase activation and mutation. In this context, further clinical studies that evaluate the relevance of LR levels or CHEK1 status to the therapeutic response to miltefosine may help identify the patients who may obtain superior benefits from miltefosine treatment.

Although P53 status is critical for selecting the therapeutic option for cancer patients, the role of P53 in the response to CHEK1‐targeting drugs or LR‐targeting drugs is not altogether clear. Here, we examined the therapeutic effect of LR disruption by using miltefosine in a panel of P53 WT and P53 mutant CRC cells (P53 wild type: SW48, HCT116 and hCRC1; P53 mutant: DLD1, HCT15, HT29, SW480, hCRC2, hCRC3 and hCRC4). First, we observed the elevation of LRs in all tested CRC cells versus normal colon cell lines. LR disruption by miltefosine efficiently retarded cell growth in all of the tested CRC cells regardless of P53 mutation (Figure 1C). Moreover, miltefosine potently reduced the CD44^high^ CSC population in both P53 WT and mutant CRC cells (Figure 2C). In accordance, the miltefosine‐induced reduction in CHEK1 mRNA and protein expression was also observed in both P53 WT and mutant CRC cells (Figure 4G). To obtain deeper insight into this phenomenon, we carried out a comprehensive analysis by using the CHEK1 inhibitor AZD7762. Treatment with AZD7762 showed a similar and potent inhibitory effect on the reduction in the CSC population and sphere‐forming efficiency in both P53‐mutant HT29 and P53 WT HCT116 cells (Figure 5J, K). These CSC‐targeting effects of CHEK1 inhibition were also effective at a similar level in P53 KO HCT116 cells (Figure 5J, K), suggesting that CHEK1 inhibition efficiently targeted CSCs regardless of P53 status. Recent studies by other groups showed that several CHEK1 inhibitors, such as SCH900776, MU380 and UCN‐01, efficiently sensitized cancer cells to conventional chemo‐radiotherapy irrespective of P53 status,^40^, ^42^, ^43^ supporting our data that AZD7762 significantly augmented the therapeutic effect of RT in all P53 WT, mutated, and deficient cell lines (Figure 5G–I). Although a careful clinical investigation is necessary to compare the therapeutic efficacy of LR‐targeting drugs between P53 WT and mutant CRC patients, our data suggest that miltefosine can target CSCs and bulk tumour cells independently of P53 status and thus has promising potential for treating a broad range of CRC patients.

LR integrity is critical for the activation of several key regulatory kinases, including focal adhesion kinase,^19^ Src,^19^, ^21^ mammalian target of rapamycin (mTOR),^74^ phosphatidylinositol‐dependent protein kinase 1 (PDK1)^18^, ^20^ and Akt.^18^, ^19^, ^20^, ^21^, ^54^ However, the precise mechanisms by which proteins sort into LRs are not yet fully understood.^12^, ^14^, ^75^ Perifosine, a potent APL drug, is a potent Akt inhibitor and has shown promising results in clinical trials for multiple types of cancer.^20^, ^30^, ^31^, ^32^ Consistently, miltefosine also exhibited an inhibitory effect on Akt phosphorylation (*p*‐Akt; Supplementary Figure S6E). Moreover, Akt phosphorylation was elevated in CD44^high^ cells compared with CD44^low^ cells (Supplementary Figure S6F). Therefore, we carried out further analysis to determine the possible link between Akt signalling and CHEK1. Interestingly, treatment with a well‐known Akt inhibitor, MK2206, significantly reduced CHEK1 expression in CRC cells (Supplementary Figure S6G) by reducing the transcriptional activity of CHEK1 (Supplementary Figure S6H). Collectively, our data suggest that Akt inhibition by miltefosine may be involved in decreasing CHEK1 expression in CRC cells. We searched for downstream molecules of Akt signalling and upstream molecules of CHEK1 using IPA to elucidate a more detailed mechanism that possibly mediated Akt‐induced CHEK1 regulation. We identified several molecules that might be involved in Akt‐mediated CHEK1 transcriptional regulation, including E2F1, E2F4, BCL6 and MYC (Supplementary Figure S6I). Thus, further investigations of this possible pathway may be highly informative for obtaining a better understanding of the mechanism of miltefosine. Together, although further analysis is still required to determine the possibility that other LR‐independent signalling or CHEK1‐independent mechanisms might be involved in the therapeutic effect of miltefosine, our findings highlight the importance of LR/CHEK1 as a key molecular mechanism that at least partially mediates the therapeutic effects of miltefosine (Supplementary Figure S7).

CSCs resist conventional therapy, retaining metastasis and recurrence potential. Mechanistically, we found that CSCs were more resistant to conventional therapy than non‐CSCs (Figure 6A–C and Supplementary Figure S6B) by exploiting the CHEK1‐mediated G2/M checkpoint and preventing cell death even under extensive DNA‐damaging stress (Figure 6D–F). However, miltefosine transcriptionally attenuated CHEK1 expression (Figure 4I), thereby promoting the entry of CSCs into the M phase, where unresolved replication stress caused detrimental mitotic catastrophe (Figures 6D–F and Supplementary Figure S6D). These data suggest miltefosine as a strong candidate for CRC treatment to improve the therapeutic resistance phenotype of CSCs. In parallel, we demonstrated that in CRC xenograft models, miltefosine dramatically reduced the burden of primary tumours, and when miltefosine‐treated tumour cells were re‐inoculated into new mice, we confirmed the significant impairment of cancer regrowth potential by miltefosine treatment (Figure 3B–F). In addition, the metastatic potential of CRC cells was dramatically reduced by miltefosine treatment (Figure 3G, H). Together, our findings indicate that LR‐disrupting miltefosine, which efficiently targets both CSCs and bulk tumour cells, exhibits strong therapeutic efficacy in preclinical models and is thus a strong candidate for CRC treatment.

Collectively, our discovery of the LR/CHK axis will broaden the potential application of LR‐disrupting drugs and may provide a preclinical rationale for the evaluation of LR‐disrupting drugs as attractive candidates for CRC treatment.

## CONFLICT OF INTEREST

The authors declare that they have no competing interests.

## Supporting information

Supporting information

Table S1

Table S3

Table S7
