# Supplementary material for: Lipid raft‐disrupting miltefosine preferentially induces the death of colorectal cancer stem‐like cells
Source: Clin Transl Med. 2021 Nov 4;11(11):e552. doi: 10.1002/ctm2.552 (PMC8567043; doi:10.1002/ctm2.552)
Supplement: Supplementary file 1 — Supporting information [file CTM2-11-e552-s004.pdf]

Supplementary Materials for

Lipid Raft-disrupting Miltefosine Preferentially Induces the Death of Colorectal Cancer Stem-like Cells

So-Yeon Park[1,2,*], Jee-Heun Kim[1,*], Jang-Hyun Choi[1], Choong-Jae Lee[1], Won-Jae Lee[1], Sehoon Park[1], Zee-Yong Park[1], Jeong-Heum Baek[3], Jeong-Seok Nam[1,2,$]

*Equal contribution

[1]School of Life Sciences, Gwangju Institute of Science and Technology, Gwangju, 61005, Republic of Korea.

[2]Cell Logistics Research Center, Gwangju Institute of Science and Technology, Gwangju, 61005, Republic of Korea.

[3]Division of Colon and Rectal Surgery, Department of Surgery, Gil Medical Center, Gachon University College of Medicine, Incheon 21565, Republic of Korea.

$Corresponding author

Jeong-Seok Nam, DVM, Ph.D.

School of Life Sciences, Gwangju Institute of Science and Technology, Gwangju, 61005, Republic of Korea. Phone: +82)62-715-2893, Fax: +82)62-715-2893, E-mail: namje@gist.ac.kr

This PDF file includes:

Figures S1-S6

Table S1

Table S4 to S6

Other Supplementary Materials for this manuscript include the following:

Table S2 (attached as a separate excel file)

Table S3 (attached as a separate excel file)

Table S7 (attached as a separate excel file)

Figure S1.

A) Fractions obtained from a sucrose gradient centrifugation were analyzed by SDS-PAGE and immunoblotting to identify Flotillin-1 and Caveolin-1 using corresponding antibodies. GM1, a ganglioside of LR were detected using cholera toxin B subunit conjugated to HRP. **B)** Representative extracted ion chromatograms (XIC) of LR fractions extracted from normal colon cell (FHC) and colorectal cancer cell (HT29). Gangliosides were extracted by Xcalibur software (Thermo Scientific, Bremen, Germany) with a mass tolerance of 5 ppm based on the identification by LipidSearch 4.2 (Thermo Scientific, Bremen, Germany). Quantification of each peak was analyzed by area under curve (AUC) and compared between normal and tumor (n=3/group). AUC of identified gangliosides was normalized by total AUC of gangliosides that were commonly detected in each group, and accounted as relative abundance. Statistical significance was determined by Student's t-test. **C)** The elevated levels of LRs in tumor tissues compared to matched normal tissues obtained from CRC patients. H&E staining and immunofluorescence staining for DAPI nuclear staining (blue) and CTxB (green) with respective merged and magnified images. LRs were visualized by using Alexa 488-conjugated CTxB, which binds to ganglioside GM1, an LR component. Samples were examined by fluorescence microscopy (Axio Imager 2, ZEISS, Oberkochen, Germany). **D)** Increased LR levels in adenomatous polyps in intestine of adenomatous polyposis coli (*Apc*$^{\mathrm{Min/+}}$) mice. Normal intestine of wild-type (WT) mice and the adenomatous intestines of *Apc*$^{Min/+}$ were compared. H&E staining and immunofluorescence staining for DAPI nuclear staining (blue) and CTxB (green) with respective merged and magnified images. LRs were visualized by using Alexa 488-conjugated CTxB, which binds to ganglioside GM1, an LR component. Samples were examined by fluorescence microscopy (Axio Imager 2, ZEISS, Oberkochen, Germany). **E)** LR disruption upon miltefosine treatment (48 hours) in dose dependent manner. Quantification of LR level were analyzed by Median Fluoresce Intensity (MFI) ratio of CTxB (Fold to Vehicle) which was normalized by Iso-Ab. (n=3/group) **F)** LR disruption upon 48-hour treatment of LR-disrupting drugs, e.g. miltefosine, MβCD, simvastatin and perifosine was measured using flow cytometry (n=3/group). Bar graph (left) shows the MFI ratio of CTxB (Fold to Vehicle) which was

normalized by Iso-Ab. Representative combined flow cytometry histogram (right) showing the effect of LR-disrupting treatment. **G)** Cell viability was determined by MTT assay after 48-hour treatment of LR-disrupting drugs, miltefosine, MβCD, simvastatin and perifosine. (n=3/group) **E, F, G)** Statistical values were analyzed by one-way ANOVA with Dunnett's multiple comparison test. **H and I)** Comparison of relative sensitivity to 5-Fluorouracil (E) and oxaliplatin (F) in colorectal cancer cell lines (HT29 and HCT116). Cells were treated with miltefosine (1µM) or DMSO (1%) and various concentrations of 5-Fluorouracil or Oxaliplatin. After 48 hours of incubations, cell viability was measured by MTT assay and the absorbance was measured using a microplate spectrophotometer (Bio-Tek Instruments Inc, Winooski, VT, USA). IC_{50} values based on reductions in cell viability were calculated by nonlinear regression model and statistical difference between two dose-response curves were determined based on extra sum-of-squares F test using GraphPad Prism v7.05 software (GraphPad, La Jolla, CA, USA). *, **, *** indicates $p < 0.05$, $p < 0.01$ and $p < 0.001$, respectively.

Figure S1

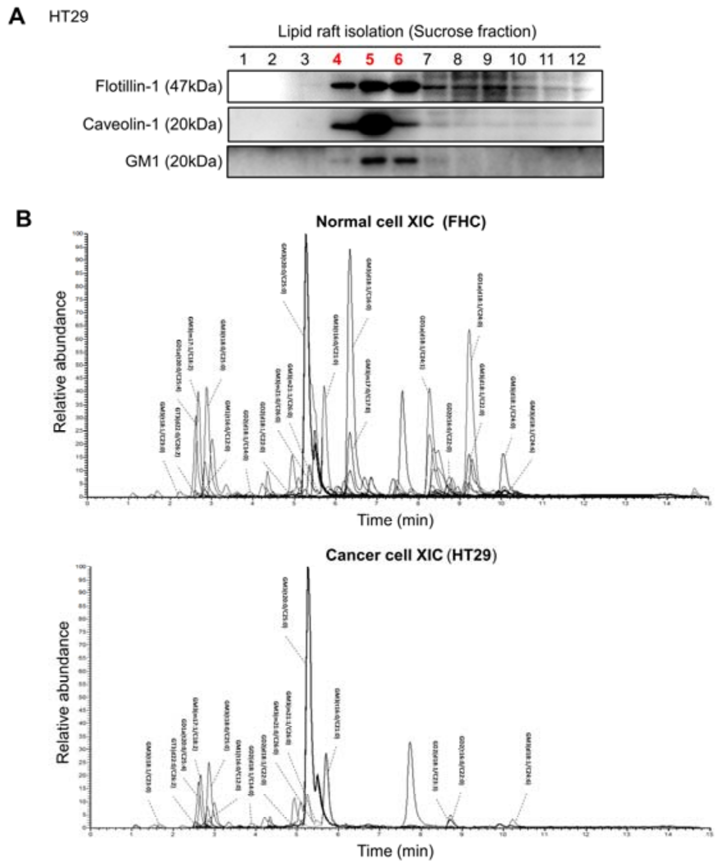

Class	Normal cell (%)	Cancer cell (%)	Fold change (Cancer/Normal)	*p*-value
GM1	0.06	0.15	2.31	0.019
GM3	25.83	46.84	1.81	0.011
GD1a	3.25	6.34	1.95	0.022
GD2	1.65	2.79	1.69	0.029
GT3	0.24	0.53	2.24	0.022

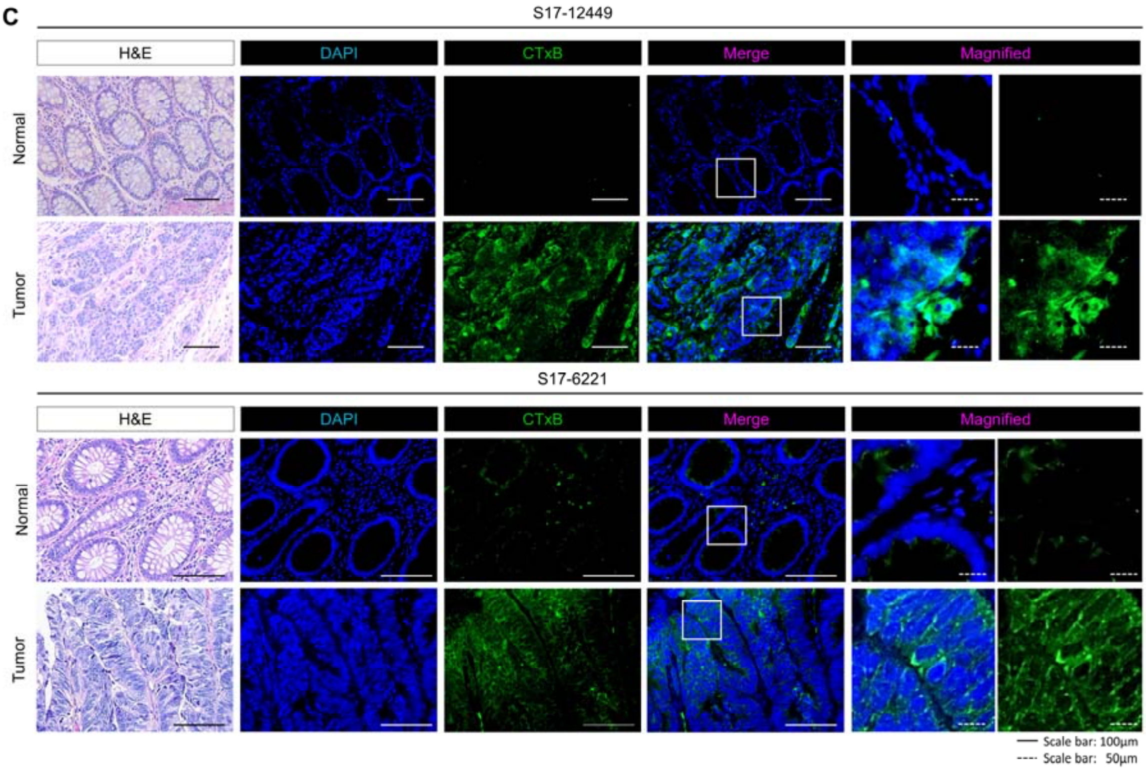

Figure S1

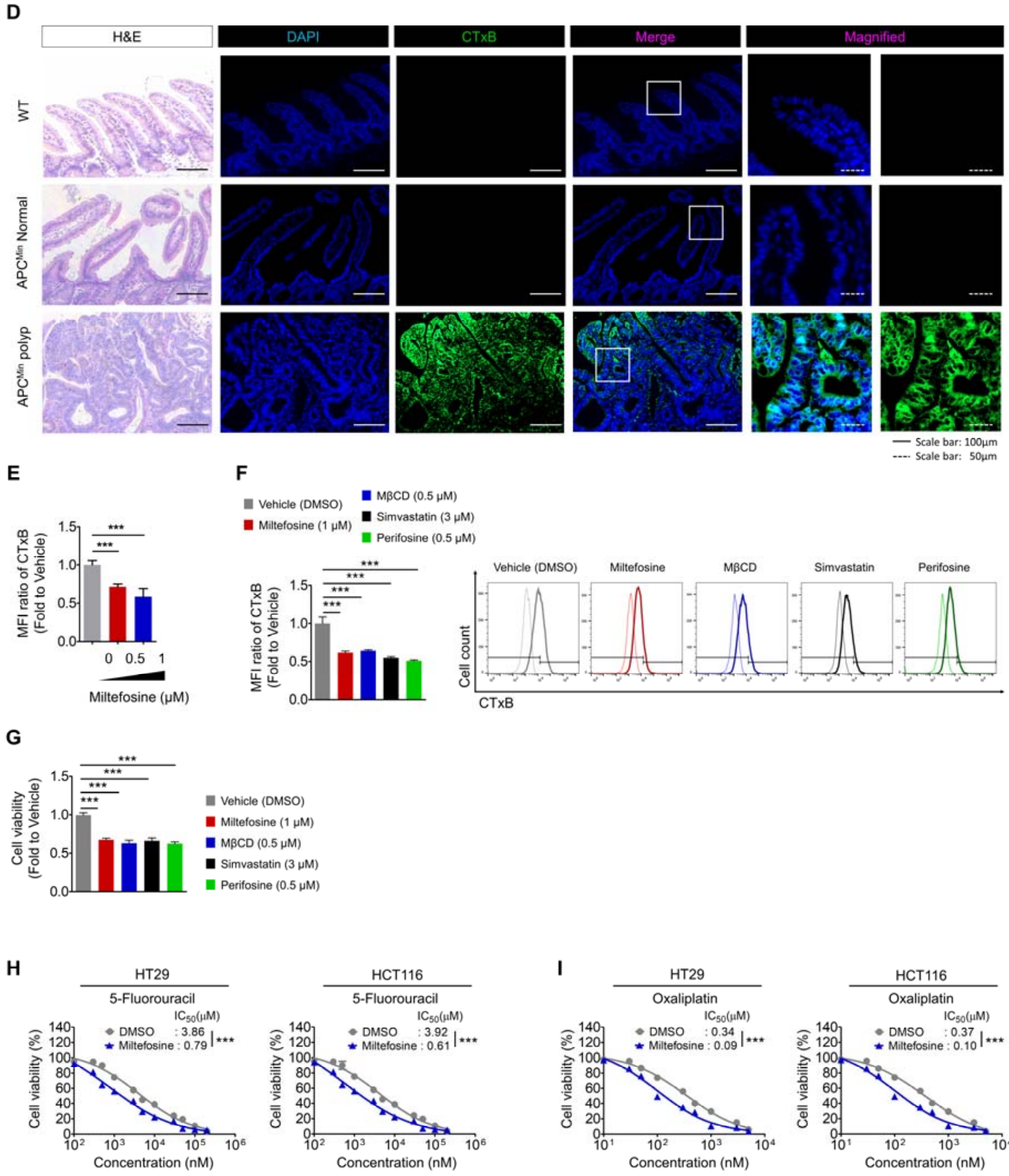
D
H&E
DAPI
CTxB
Merge
Magnified
WT
APCMin Normal
APCMin polyp
Scale bar: 100μm
Scale bar: 50μm
E
MFI ratio of CTxB (Fold to Vehicle)
Miltefosine (μM)
F
Vehicle (DMSO)
Miltefosine (1 μM)
MβCD (0.5 μM)
Simvastatin (3 μM)
Perifosine (0.5 μM)
Cell count
CTxB
G
Cell viability (Fold to Vehicle)
H
HT29
HCT116
5-Fluorouracil
IC50(μM)
DMSO : 3.86
Miltefosine : 0.79
DMSO : 3.92
Miltefosine : 0.61
Cell viability (%)
Concentration (nM)
I
Oxaliplatin
DMSO : 0.34
Miltefosine : 0.09
DMSO : 0.37
Miltefosine : 0.10

73

Figure S2.

A) A solid isolation of CD44^{high} and CD44^{low} was performed using BD FACSAria™ III cell sorter (BD Bioscience, New Jersey, USA) after being cultured in Sphere culture condition. 2D scatter plot of flow cytometry which confirming the enrichment of CD44^{high} population in sphere culture condition compared to monolayer culture condition was analyzed using BD Accuri™ C6 (BD Bioscience) and FlowJo software (TreeStar, Ashland, OR, USA). **B)** Flow cytometry 2D scatter plots showing cells doubly stained with CTxB and anti-CD44 antibody. The gates were established by staining with corresponding isotype antibody. **C)** Comparison of LR levels in CD133^{high} and CD133^{low} populations, or in ALDH1^{high} and ALDH1^{low} populations. Cells were doubly stained with CTxB and anti-CD44/or ALDH1 antibody, and analyzed by FACS. Bar graph shows the MFI ratio of CTxB which was normalized by Iso-Ab within the indicated population (mean ± SEM, n=3/group). Statistical calculations were analyzed by Student's t-test. **D)** Representative combined flow cytometry histograms (top) showing the effect of miltefosine treatment (0.5 μM and 1 μM, 48-hour treatment) on CD44^{high} populations in dose dependent manner. Table (bottom) shows the quantification of CD44^{high} populations upon miltefosine treatment and presented by mean ± SEM (n=3/group). **E)** 2D scatter plot (left) showing the differential effect of miltefosine treatment on CD44^{high} and CD44^{low} cell population. Sorted cells were treated with miltefosine (1 μM) for 48 hours and live/or dead cells were detected by staining with Calcein-AM and PI, and then analyzed by FACS. Observed dead cell populations from each group were quantified and presented in a bar graph (right, mean ± SEM, n=3). Statistical significance was analyzed by one-way ANOVA with Dunnett's multiple comparison. **F)** 2D scatter plot of flow cytometry confirming a solid isolation of CD44^{high} and CD44^{low} populations in hCRC2 cells after being cultured in sphere condition. **G**) 2D scatter plot (left) showing the differential effect of 48-hour miltefosine treatment on CD44^{high} and CD44^{low} cell population in hCRC2 cells. Live/or dead cells detected as described in Figure S2E. Bar graph shows the percentage of dead cells in the population (mean ± SEM (n=3)). Statistical significance was analyzed by one-way ANOVA, with Dunnett's multiple comparison.

H) Comparison of miltefosine sensitivity in multiple cell populations of HT29 cells; Bulk cancer cells, sorted $CD44^{high}$ cells and sorted $CD44^{low}$ cells. Cells were treated with various concentration of miltefosine and incubated for 48 hours. And then, cell viability was measured by MTT assay and the absorbance values were detected by a microplate spectrophotometer. Based on cell viability, IC_{50} values were determined via nonlinear regression model calculation. Statistical difference among three dose-response curves was analyzed based on extra sum-of-squares F test using GraphPad Prism. **I)** Level of LR in sphere upon LR-disrupting drug treatment (48hours) e.g., miltetosine, MβCD, simvastatin, perifosine. Representative combined flow cytometry histogram (left) showing the effect of LR-disrupting treatment in CSC while bar graph (right) shows the MFI ratio of CTxB (Fold to Vehicle) which was normalized by Iso-Ab (n=3/group). **J**) Sphere viability was determined by resazurin-based Cell Titer Blue assay on the 7th day of sphere seeding. LR-disrupting drugs were administered every other day throughout the sphere generation. Statistical significance was determined by one-way ANOVA with Dunnett's multiple comparison. **K)** Confirmation of CSC enrichment under sphere culture condition by RT-qPCR. Relative mRNA levels of stemness markers and differentiation markers were examined in monolayer cultured cells and sphere cultured cells (n=3/group). The heatmap represents the value of log_2FC in the range of -5 to +5 with $p < 0.05$ (n=3/group). Statistical calculation was analyzed by Student's t-test. **L)** 2D scatter plot of flow cytometry confirming a solid isolation of $CTxB^{high}$ and $CTxB^{low}$ populations in HT29 cells after being cultured in sphere condition. *, **, *** indicates $p < 0.05$, $p < 0.01$ and $p < 0.001$, respectively.

Figure S2

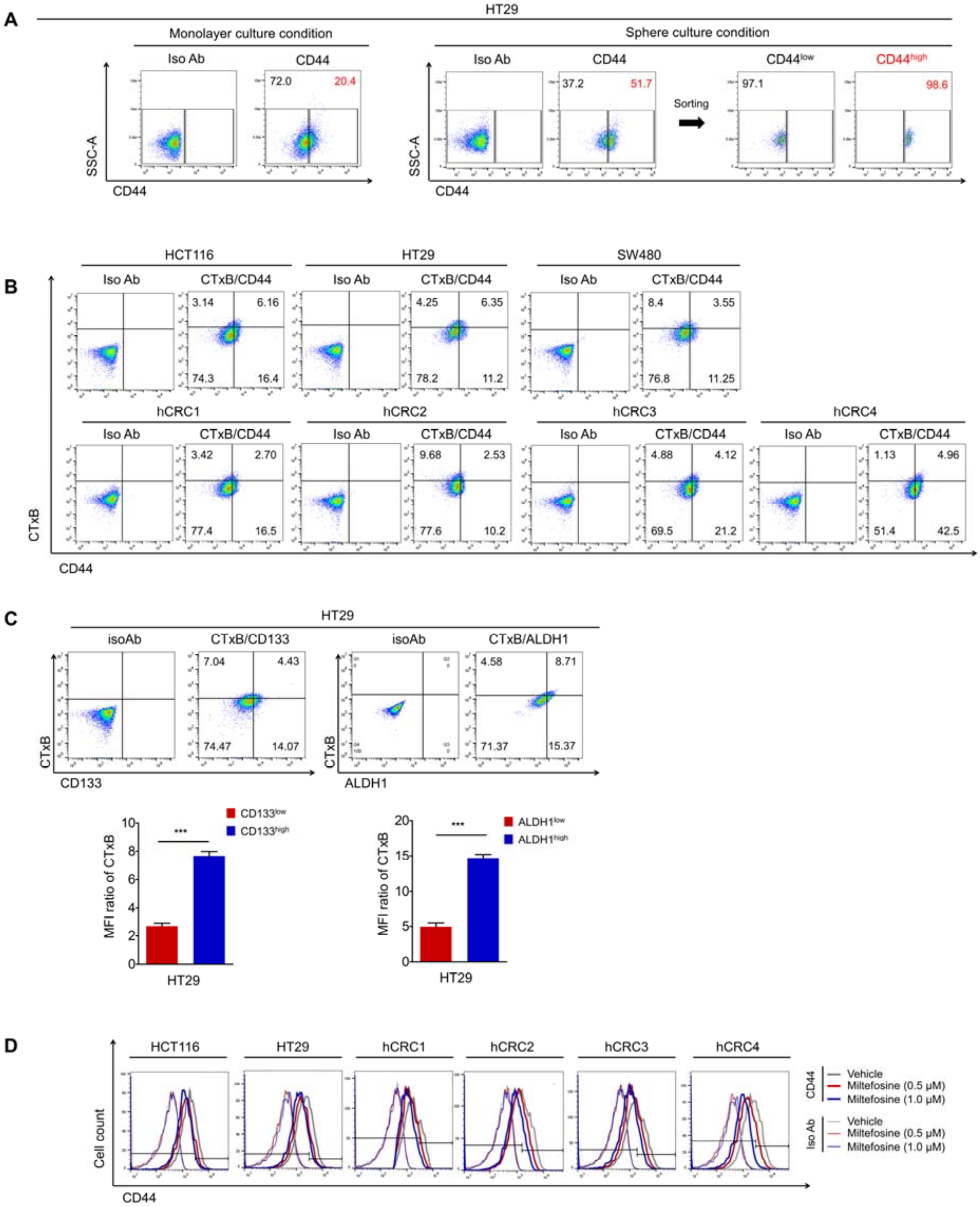

Group	CD44high population (%)					
	HCT116	HT29	hCRC1	hCRC2	hCRC3	hCRC4
Vehicle	19.93 ± 0.37	26.39 ± 2.94	22.79 ± 2.69	26.65 ± 1.17	21.65 ± 0.53	23.74 ± 1.72
Miltefosine (0.5μM)	9.30 ± 0.52	18.01 ± 0.79	19.07 ± 1.61	9.26 ± 1.10	8.81 ± 0.44	10.59 ± 1.11
Miltefosine (1.0μM)	5.37 ± 0.43	5.90 ± 0.84	9.89 ± 0.73	3.58 ± 0.57	1.12 ± 0.21	2.45 ± 0.09

Figure S2

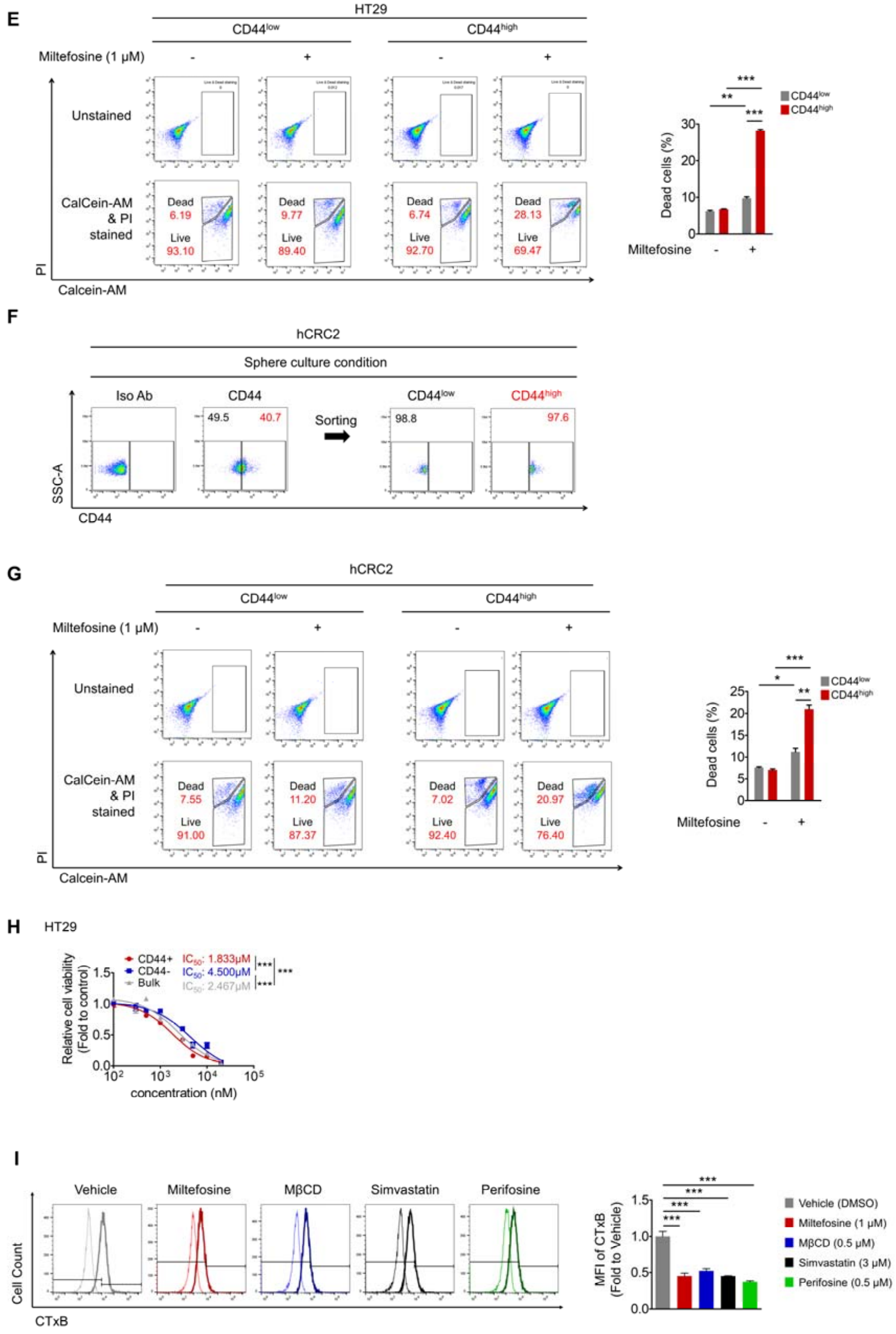
E
HT29
CD44low
CD44high
Miltefosine (1 μM)
Unstained
CalCein-AM & PI stained
Dead 6.19
Live 93.10
Dead 9.77
Live 89.40
Dead 6.74
Live 92.70
Dead 28.13
Live 69.47
PI
Calcein-AM
Dead cells (%)
Miltefosine
F
hCRC2
Sphere culture condition
Iso Ab
CD44
49.5
40.7
Sorting
CD44low
CD44high
98.8
97.6
SSC-A
CD44
G
hCRC2
CD44low
CD44high
Miltefosine (1 μM)
Unstained
CalCein-AM & PI stained
Dead 7.55
Live 91.00
Dead 11.20
Live 87.37
Dead 7.02
Live 92.40
Dead 20.97
Live 76.40
PI
Calcein-AM
Dead cells (%)
Miltefosine
H
HT29
CD44+ IC50: 1.833μM
CD44- IC50: 4.500μM
Bulk IC50: 2.467μM
Relative cell viability (Fold to control)
concentration (nM)
I
Vehicle
Miltefosine
MβCD
Simvastatin
Perifosine
Cell Count
CTxB
MFI of CTxB (Fold to Vehicle)
Vehicle (DMSO)
Miltefosine (1 μM)
MβCD (0.5 μM)
Simvastatin (3 μM)
Perifosine (0.5 μM)

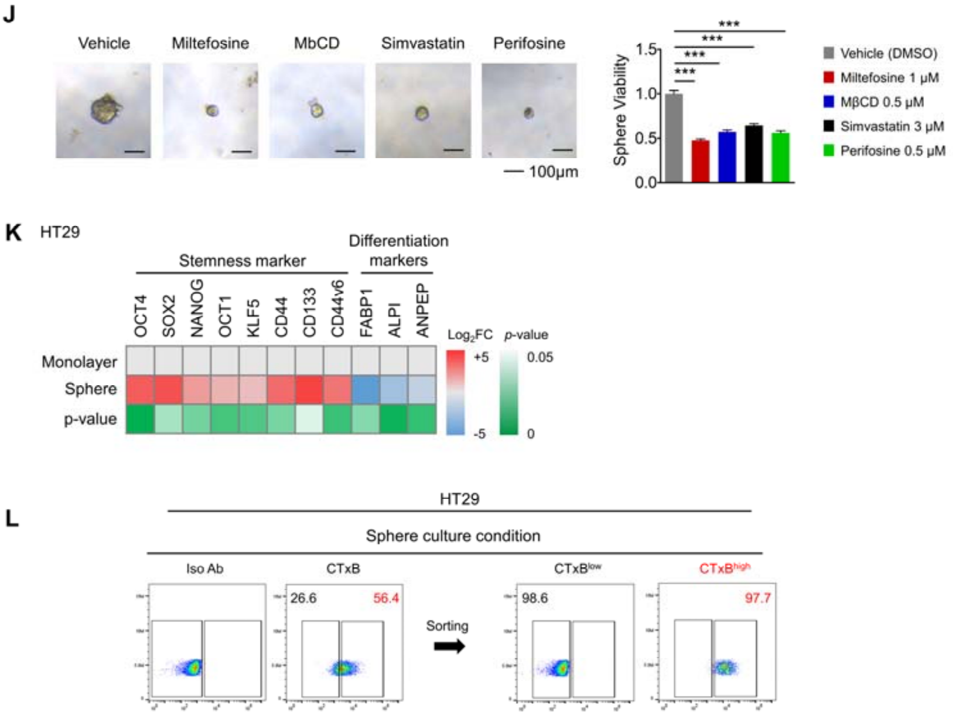
J
Vehicle
Miltefosine
MbCD
Simvastatin
Perifosine
100μm
Sphere Viability
1.5
1.0
0.5
0.0
***
Vehicle (DMSO)
Miltefosine 1 μM
MβCD 0.5 μM
Simvastatin 3 μM
Perifosine 0.5 μM
K
HT29
Stemness marker
Differentiation markers
OCT4
SOX2
NANOG
OCT1
KLF5
CD44
CD133
CD44v6
FABP1
ALPI
ANPEP
Monolayer
Sphere
p-value
Log2FC
+5
-5
p-value
0.05
0
L
HT29
Sphere culture condition
Iso Ab
CTxB
26.6
56.4
Sorting
CTxBlow
98.6
CTxBhigh
97.7

Figure S3.

A) Comparison of LR levels in HT29 cells upon miltefosine treatment under various culture conditions: monolayer, 1st sphere generation and 2nd sphere generation. CTxB levels were compared between DMSO- and miltefosine-treated cells. DMSO and 1 μM miltefosine were administered every other day throughout the 1st sphere generation and monolayer culture for 7 days. Then, the cells were collected, prepared as single-cell suspensions and replated for the 2nd sphere generation assay without further miltefosine treatment. 2D histograms (left) show cells stained with the CTxB antibody. Quantification of CTxB levels in each condition and treatment are shown as a bar graph (right panel). Bar graphs indicate the MFI ratio of CTxB (fold change compared to the untreated monolayer), ± SEM (n=3/group). **B)** Representative immunofluorescent images (left) and quantitative analysis (right) of xenografted tumors (1st generation) comparing the control and miltefosine-treated groups (Figure 3C). Relative fluorescent intensity of Ki67 (green) and CD44 (red) was quantified by using Image pro premier 9 (Media Cybermetics) with normalization to DAPI intensity. Quantification was performed in three randomly selected fields for each specimen (n=8/group). Statistical calculations were analyzed by Student's t-test**.** Bar graph shows mean ± SEM (n=8/group). **C)** Comparison of body weight changes of both control and miltefosine treated groups from Day 0 to Day 21 post treatment. The graph shows mean ± SEM (n=8/group). Statistical significance between body weight changes of both groups was determined by Two-way repeated-measures ANOVA followed by Bonferroni posttests. **D**) Three representative immunohistochemistry images of liver and kidney of control and miltefosine treated group. **E)** To examine the effect of miltefosine on tumor regrowth potential, tumor cells were isolated from the 1st generation tumors (Figure 3C) and reinjected into NSG mice (12,500, 25,000, 50,000 and 100,000 cells/mouse, n=6/group) for *in vivo* LDA. No miltefosine treatment was applied during this in vivo LDA. The regrowth of tumors (2nd generation) were monitored for 28 days. On 28th day after reinjection, mice were sacrificed and the definite incidence of tumor-bearing mice was determined. The frequency of stem cells per each group and statistical value were analyzed by a webtool (http://bioinf.wehi.edu.acu/software/elda). *** indicates $p < 0.001$.

Figure S3

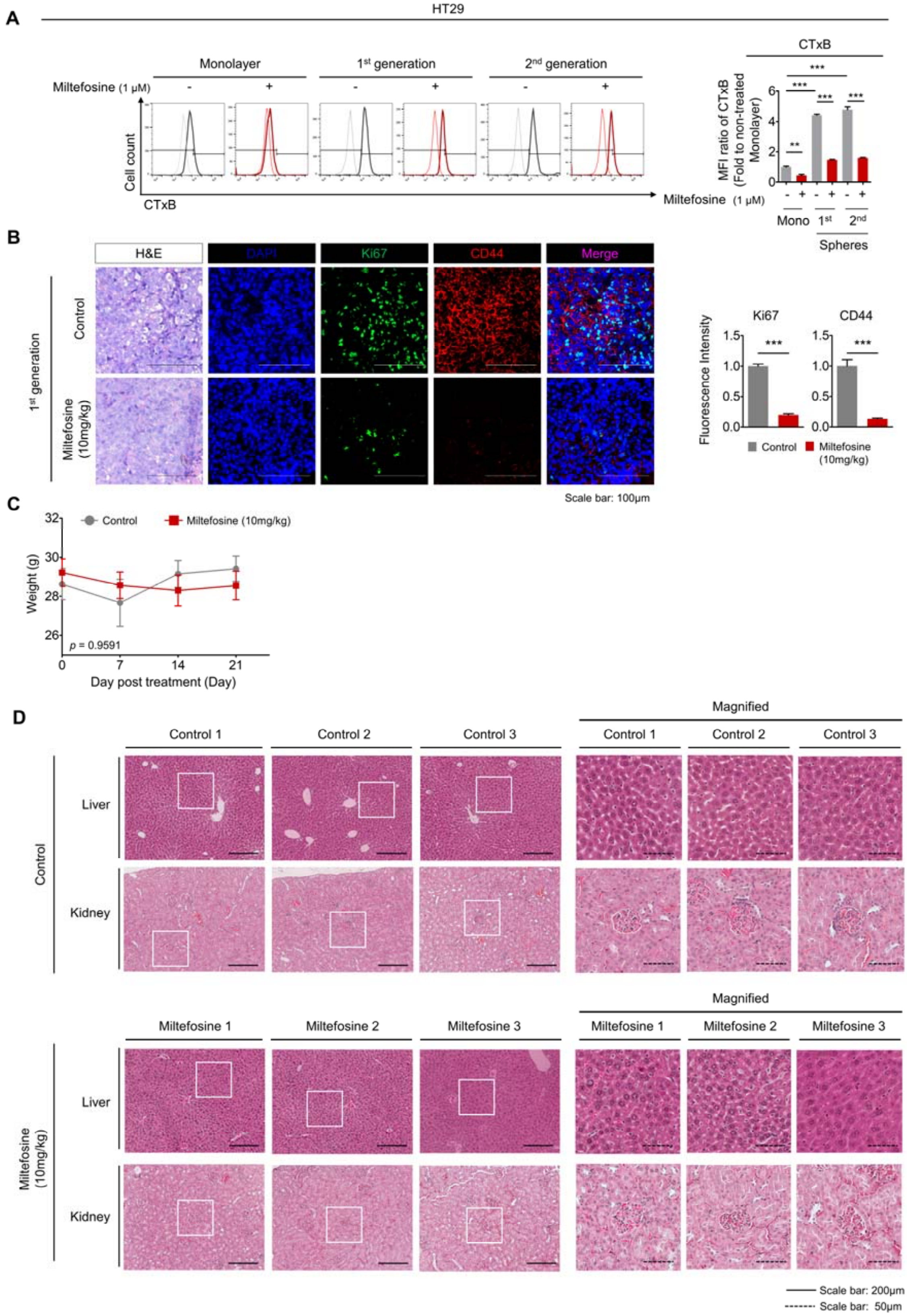

149

Figure S3

E

Frequency of tumor formation									
Cell no. of inoculation	$10x10^4$		$5x10^4$		$2.5x10^4$		$1.25x10^4$		*p*-values
	Control	Miltefosine (10mg/kg)	Control	Miltefosine (10mg/kg)	Control	Miltefosine (10mg/kg)	Control	Miltefosine (10mg/kg)	
Day4	0% (0 of 6)	0% (0 of 6)	0% (0 of 6)	0% (0 of 6)	0% (0 of 6)	0% (0 of 6)	0% (0 of 6)	0% (0 of 6)	-
Day7	0% (0 of 6)	0% (0 of 6)	0% (0 of 6)	0% (0 of 6)	0% (0 of 6)	0% (0 of 6)	0% (0 of 6)	0% (0 of 6)	-
Day11	0% (0 of 6)	0% (0 of 6)	0% (0 of 6)	0% (0 of 6)	0% (0 of 6)	0% (0 of 6)	0% (0 of 6)	0% (0 of 6)	-
Day14	0% (0 of 6)	0% (0 of 6)	0% (0 of 6)	0% (0 of 6)	0% (0 of 6)	0% (0 of 6)	0% (0 of 6)	0% (0 of 6)	-
Day18	67% (4 of 6)	0% (0 of 6)	0% (0 of 6)	0% (0 of 6)	0% (0 of 6)	0% (0 of 6)	0% (0 of 6)	0% (0 of 6)	$1.13x10^{-2}$
Day21	67% (4 of 6)	17% (1 of 6)	17% (1 of 6)	0% (0 of 6)	17% (1 of 6)	0% (0 of 6)	0% (0 of 6)	0% (0 of 6)	$2.40x10^{-2}$
Day25	83% (5 of 6)	50% (3 of 6)	67% (4 of 6)	17% (1 of 6)	50% (3 of 6)	17% (1 of 6)	0% (0 of 6)	0% (0 of 6)	$1.76x10^{-2}$
Day28	100% (6 of 6)	67% (4 of 6)	83% (5 of 6)	17% (1 of 6)	50% (3 of 6)	17% (1 of 6)	50% (3 of 6)	0% (0 of 6)	$3.03x10^{-4}$

Figure S4.

A) The significantly altered genes upon miltefosine treatment were analyzed by the web-based bioinformatics tool, Enrichr (http://amp.pharm.mssm.edu/Enrichr/), to predict significantly altered signaling pathways associated to miltefosine treatment. The results from this analysis showed the potential protein kinases associated with miltefosine together with their p-value and combined score. A combined score multiplies the log of the p-value computed with the Fisher exact test by the z-score computed by assessing the deviation from the expected rank. **B)** Gene set enrichment analysis (GSEA) was performed with microarray data of differential expressing gene comparing DMSO- and miltefosine treated HT29 cells. The entire gene lists were applied for GSEA analysis, which included gene sets from MSigDB pathways and C2: curated gene sets (c2.all.v6.2.symbols.gmt). FDR q-value < 0.05 was set as the significance threshold. **C)** Flow cytometric analysis of CHEK1 and CTxB in HT29 cells. 2D scatter plot (left) shows the cells doubly stained with CTxB and anti-CHEK1 antibody. Comparison of CHEK1 expression levels in $CTxB^{high}$ and $CTxB^{low}$ populations. Bar graph shows the MFI ratio of CHEK1 within $CTxB^{high}$ or $CTxB^{low}$ cells (mean ± SEM, n=3/group). Statistical values were analyzed by Student's t-test. **D)** Comparison of LR levels and CHEK1 expression levels in HT29 cells upon miltefosine treatment under various culture conditions: monolayer, 1st sphere generation and 2nd sphere generation. CTxB levels and CHEK1 expression levels were compared between DMSO- and miltefosine-treated cells. DMSO and 1 μM miltefosine were administered every other day throughout the 1st sphere generation and monolayer culture for 7 days. After 7 days of 1st sphere generation, cells were collected, prepared as single-cell suspensions and replated for the 2nd sphere generation assay without further miltefosine treatment. 2D scatter plots (left panel) show cells doubly stained with CTxB and the anti-CHEK1 antibody. Quantification of CTxB and CHEK1 expression in the whole cell population and CHEK1 expression in $CTxB^{high}$ cells are shown as bar graphs (right panel). Bar graphs indicate the MFI ratio of parameters (fold change compared to untreated monolayer), ± SEM (n=3/group). Data are shared with Figure S3A which is modified to solely represent the level of CTxB. **E)** Comparison of LR levels and CHEK1 expression levels in HT29 cells upon 48-hour miltefosine

treatment. 2D scatter plots (left) show cells doubly stained with CTxB and anti-CHEK1 antibody. Quantification of CTxB level and CHEK1 expression in whole cell population and CHEK1 expression in CTxB^{high} cells were shown as bar graphs. Bar graphs indicate MFI ratio mean ± SEM (n=3/group). **D-E)** Statistical analysis was performed by one-way ANOVA, with Dunnett's multiple comparison. **F)** Protein expression of CHEK1 in normal and tumor tissues obtained from colorectal cancer patients. β-actin was used as a loading control. Values below each lane indicate the relative band intensity of target protein normalized to β-actin as fold to control lane. **G and H)** Clinical data analyzed obtained from a web-based data mining platform, Oncomine. (**G**) Comparison of CHEK1 mRNA levels between in CRC tissues (n=36) and normal colorectal tissues (n=24) in Skrzypczak colorectal dataset (GSE20916). (**H**) Comparison of CHEK1 mRNA levels between in stage 1 CRC tissues (n=4) and stage 3 CRC tissues (n=27) in GSE35834 dataset. Statistical significance was determined by Student's t-test. **I)** The prognostic value of CHEK1 in patients with CRC using GSE24551 dataset. Patients were divided into two groups according to CHEK1 expression levels. Kaplan-Meier analysis and statistical calculation were conducted by R2: Genomics analysis and visualization platform. **J, K)** Comparison of CHEK1 expression levels in CD44^{high} and CD44^{low} populations or in CD133^{high} and CD133^{low} populations. Representative 2D scatter plots show (J) HT29 and (K) HCT116 cells doubly stained with anti-CHEK1 and anti-CD44/or CD133 antibody. **L)** Comparison of CHEK1 expression levels in ALDH1^{high} and ALDH1^{low} populations. HT29 and HCT116 cells were double stained with anti-CHEK1 and anti-ALDH1 antibody, and analyzed by FACS. Bar graph (right) shows the MFI ratio of CHEK1 expression within the indicated populations (mean ± SEM, n=3/group). Statistical significance was determined by Student's t-test. *, **, *** indicates $p < 0.05$, $p < 0.01$ and $p < 0.001$, respectively.

Figure S4

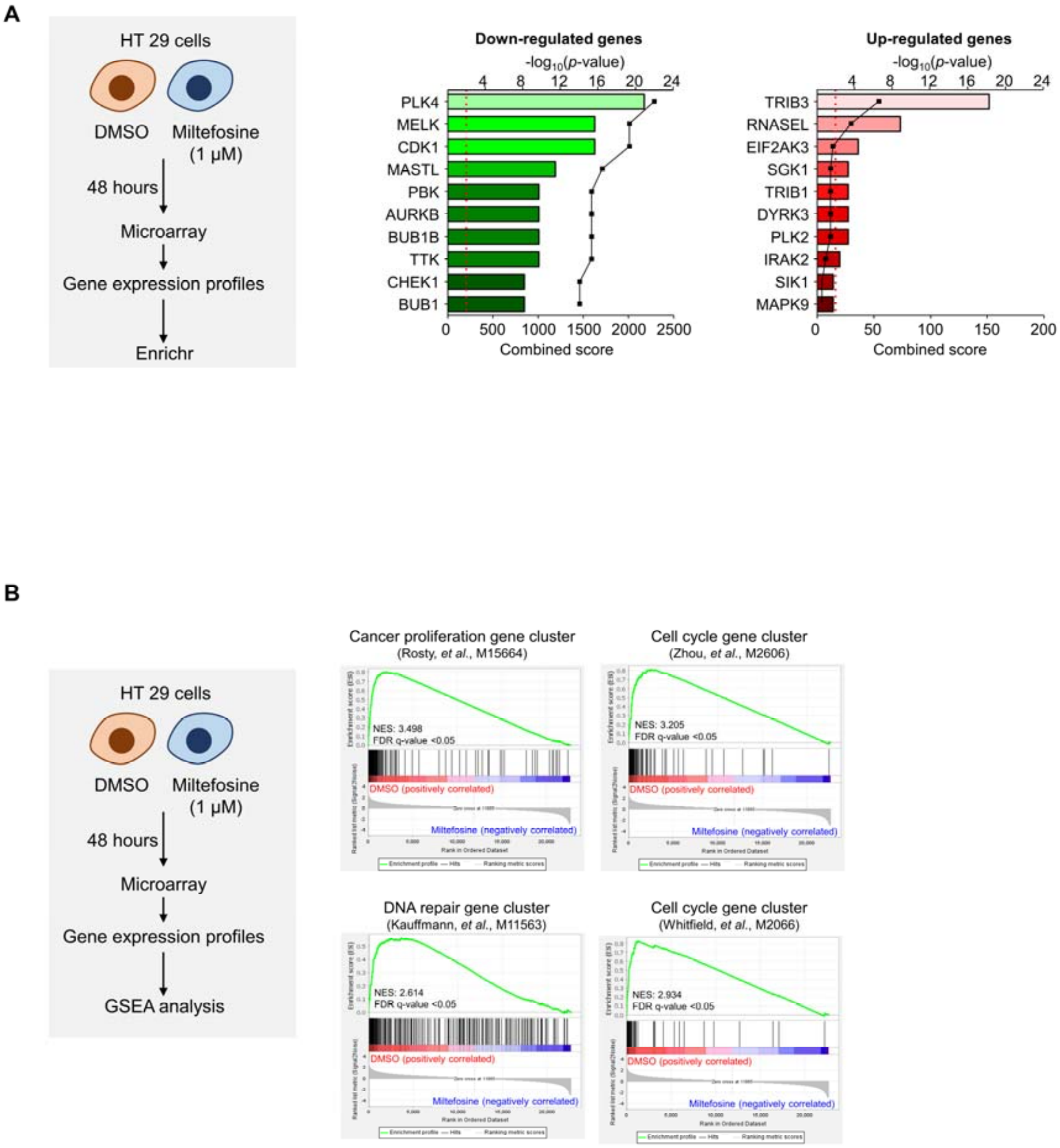

199

Figure S4

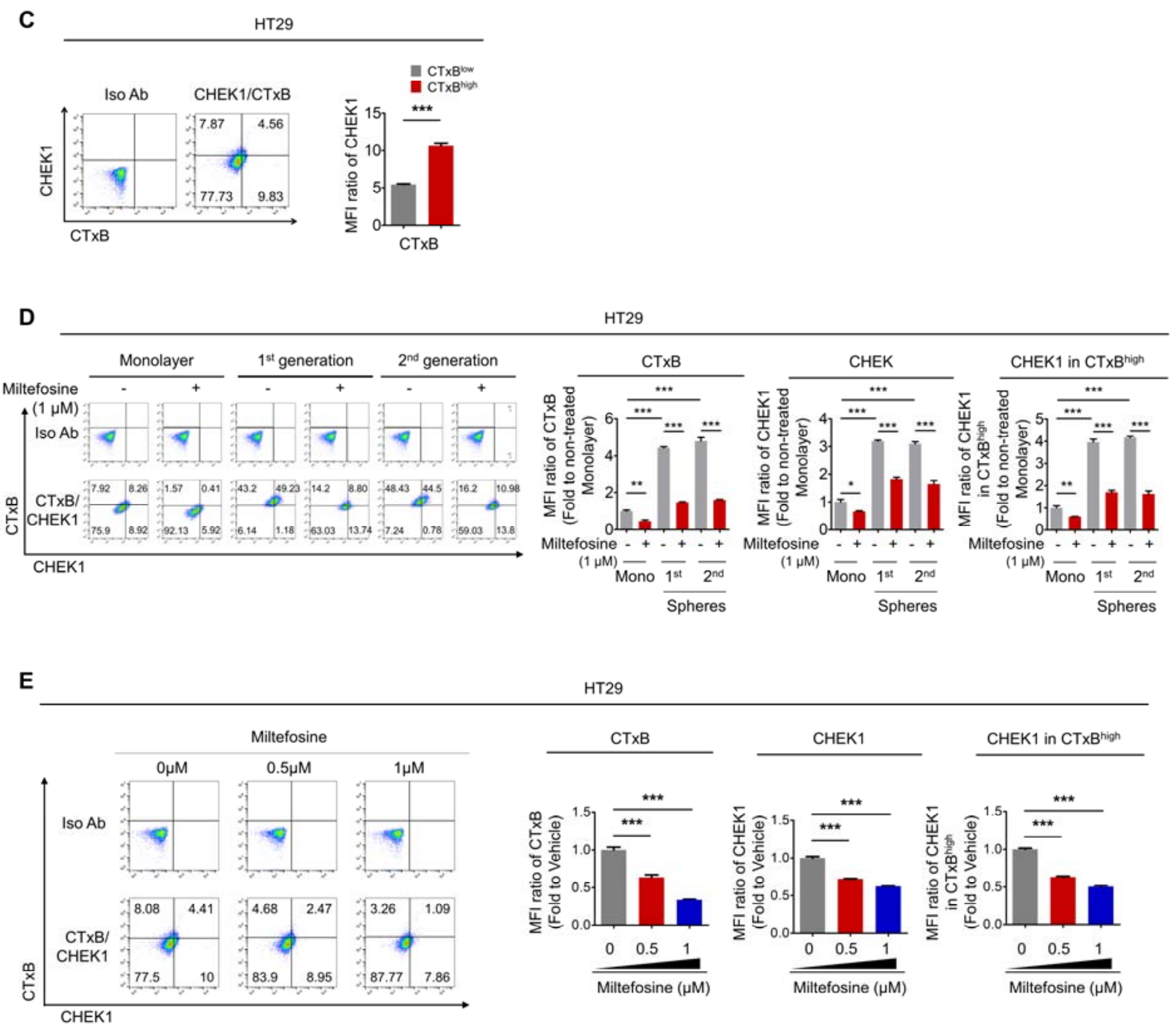
C
HT29
Iso Ab
CHEK1/CTxB
CHEK1
CTxB
7.87
4.56
77.73
9.83
CTxBlow
CTxBhigh
MFI ratio of CHEK1
***
CTxB
D
HT29
Monolayer
1st generation
2nd generation
Miltefosine
(1 μM)
Iso Ab
CTxB/
CHEK1
CTxB
CHEK1
7.92
8.26
75.9
8.92
1.57
0.41
92.13
5.92
43.2
49.23
6.14
1.18
14.2
8.80
63.03
13.74
48.43
44.5
7.24
0.78
16.2
10.98
59.03
13.8
CTxB
CHEK
CHEK1 in CTxBhigh
MFI ratio of CTxB (Fold to non-treated Monolayer)
MFI ratio of CHEK1 (Fold to non-treated Monolayer)
MFI ratio of CHEK1 in CTxBhigh (Fold to non-treated Monolayer)
Mono
1st
2nd
Spheres
E
HT29
Miltefosine
0μM
0.5μM
1μM
Iso Ab
CTxB/
CHEK1
CTxB
CHEK1
8.08
4.41
77.5
10
4.68
2.47
83.9
8.95
3.26
1.09
87.77
7.86
CTxB
CHEK1
CHEK1 in CTxBhigh
MFI ratio of CTxB (Fold to Vehicle)
MFI ratio of CHEK1 (Fold to Vehicle)
MFI ratio of CHEK1 in CTxBhigh (Fold to Vehicle)
Miltefosine (μM)

Figure S4

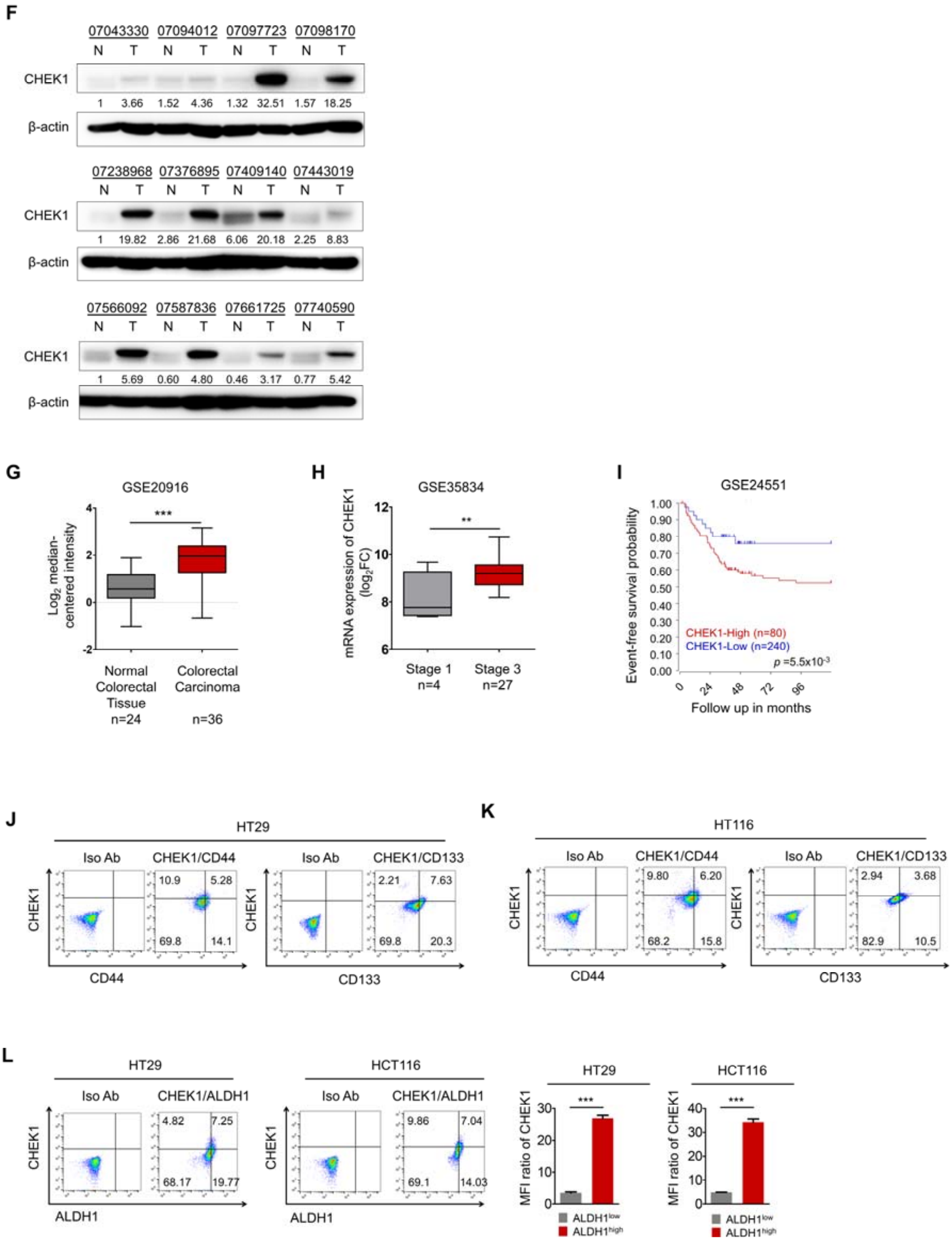
F
07043330 07094012 07097723 07098170
N T N T N T N T
CHEK1
1 3.66 1.52 4.36 1.32 32.51 1.57 18.25
β-actin
07238968 07376895 07409140 07443019
CHEK1
1 19.82 2.86 21.68 6.06 20.18 2.25 8.83
β-actin
07566092 07587836 07661725 07740590
CHEK1
1 5.69 0.60 4.80 0.46 3.17 0.77 5.42
β-actin
G
GSE20916
Log2 median-centered intensity
Normal Colorectal Tissue n=24
Colorectal Carcinoma n=36
H
GSE35834
mRNA expression of CHEK1 (log2FC)
Stage 1 n=4
Stage 3 n=27
I
GSE24551
Event-free survival probability
CHEK1-High (n=80)
CHEK1-Low (n=240)
p =5.5x10-3
Follow up in months
J
HT29
Iso Ab
CHEK1/CD44
CHEK1/CD133
CHEK1
CD44
CD133
K
HT116
Iso Ab
CHEK1/CD44
CHEK1/CD133
CHEK1
CD44
CD133
L
HT29
HCT116
Iso Ab
CHEK1/ALDH1
CHEK1
ALDH1
MFI ratio of CHEK1
ALDH1low
ALDH1high

Figure S5.

A-B) Confirmation of knockdown efficiency of three different siCHEK1 in HT29 cells. **A**) Relative mRNA expression of CHEK1 in siCHEK1-transfected cells were determined by RT-qPCR after 48 hours of siCHEK1 transfection. Bar graph shows the mean ± SEM (n=3/group). Statistical values were examined by one-way ANOVA with Dunnett's multiple comparison test. **B)** Protein expression of CHEK1 in siCHEK1-transfected cells (two most efficient CHEK1 knockdown cells confirmed by relative mRNA expressional comparison) was compared to siCTRL-transfected cells after 48 hours of siRNA transfection. Values below each lane indicate the relative band intensity of target protein normalized to β-actin as fold to control lane. **C)** Persistence of maximum efficacy of siCHEK1 was examined at different time points after transfection, from 24 hours to 96 hours. Relative mRNA expression (left) and protein expression (right) of CHEK1 upon siRNA transfection were measured and compared at different time points. Bar graphs indicates mean ± SEM (n=3/group) and statistical values were examined by Student's t-test. **D**) Flow cytometric histogram of cell cycle analysis at 48 hours after siCHEK1 transfection. **E**) Reduction of $CTxB^{high}$ populations at 48 hours after siCHEK1 transfection. Bar graph (right) indicates the relative amount of $CTxB^{high}$ population in each group by mean ± SEM (n=3/group). Statistical significance was determined by one-way ANOVA with Dunnett's multiple comparison test. **F**) Altered gene expressions of stem-related markers in HT29 cells at 48 hours after siCHEK1 transfection. Gene expression levels were determined by RT-qPCR and the heatmap shows the relative mRNA levels of indicate genes. The colors of the heatmap represent the value of log_2FC ranged from -5 to 0 with $p < 0.05$. Statistical analysis was performed by Student's t-test per each indicated gene. **G)** Confirmation of overexpression of CHEK1 in HT29 cell line. Protein expression of CHEK1 (left) and relative mRNA expression of CHEK1 (right) were compared to EV-transfected cells. Bar graphs shows mean ± SEM (n=3/group) and statistic value was determined by one-way ANOVA with Dunnett's multiple comparison test. Values below each lane indicate the relative band intensity of target protein normalized to β-actin as fold to control lane. **H)** Comparison of miltefosine sensitivity between EV-transfected cells and CHEK1-overexpressing cells in HT29. Cells were treated with

various concentration of miltefosine. After 48 hours of treatment, cell viability was measured by staining with MTT and the absorbance was measured using a microplate spectrophotometer. IC_{50} values based on reductions in cell viability were calculated by nonlinear regression model and statistical significance between two dose-response curves were determined based on extra sum-of-square F test using GraphPad Prism. **I**) Restoring effect of CHEK1 overexpression on the miltefosine-induced decrease in sphere-forming ability of CRC cells. EV-transfected cells and CHEK1-overexpresssing cells were treated with DMSO or 1μM miltefosine every other day for 7 days. And the sphere-forming efficiency was calculated as follow: number of formed sphere/number of plated cells. Statistical significance was determined by one-way ANOVA with Dunnett's multiple comparison test. **J**) Restoring effect of CHEK1 overexpression the miltefosine-induced decreases in mRNA expressions of stem-related markers (CD44, CD133, OCT4, SOX2). EV-transfected cells and CHEK1-overexpresssing cells were treated with DMSO or 1μM miltefosine for 48 hours. **K)** Confirmation of p53 status in CRC cell lines, HT29, P53 WT HCT116 and P53 KO HCT116 cell lines. Protein expression of P53 was confirmed in both low and short exposure condition. Values below each lane indicate the relative band intensity of target protein normalized to β-actin as fold to control lane. **L**) Effect of AZD7762 on cell viability was performed in HT29, P53 WT HCT116 and P53 KO HCT116 cells. Cells were treated with various concentration of AZD7762 for 48 hours. After the incubation, cell viability was measured by MTT assay to IC_{50} values using nonlinear regression models. **M)** Flow cytometry analysis of apoptotic cells after 48-hour of AZD7762 treatment. Apoptotic cells were indicated by Annexin V^+. Bar graph (right) indicated the quantification of Annexin V^+ populations upon AZD7762 treatment (mean ± SEM, n=3/group). Statistical significance was determined by one-way ANOVA with Dunnett's multiple comparison test. **N**) Reduction of $CD44^{high}$ population after AZD7762 treatment in various CRC cells. Cells were treated with DMSO, 0.1 or 0.3μM AZD7762 for 48 hours. Combined flow cytometric histograms (top) show the reduction of $CD44^{high}$ population upon AZD7762 treatment in various p53 status cell lines (HT29, P53 WT HCT116 and P53 KO HCT116). Table (bottom)

represents the quantification of $CD44^{high}$ populations (mean ± SEM, n=3/group). *, **, *** indicates $p < 0.05$, $p < 0.01$ and $p < 0.001$, respectively.

Figure S5

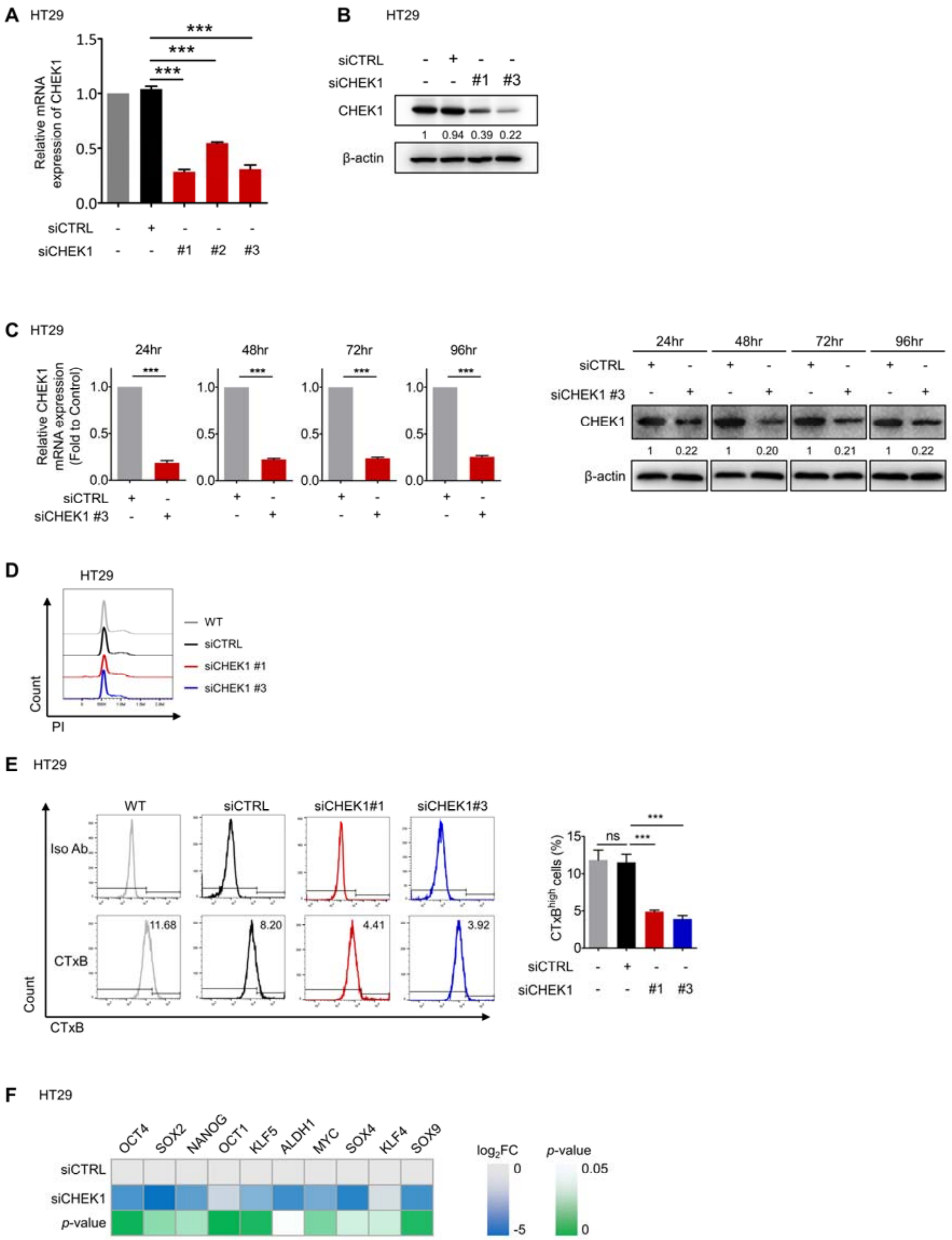
A HT29
Relative mRNA expression of CHEK1
1.5
1.0
0.5
0.0
***
***
***
siCTRL - + - - -
siCHEK1 - - #1 #2 #3
B HT29
siCTRL - + - -
siCHEK1 - - #1 #3
CHEK1
1 0.94 0.39 0.22
β-actin
C HT29
24hr
48hr
72hr
96hr
Relative CHEK1 mRNA expression (Fold to Control)
siCTRL + -
siCHEK1 #3 - +
CHEK1
1 0.22
1 0.20
1 0.21
1 0.22
β-actin
D HT29
WT
siCTRL
siCHEK1 #1
siCHEK1 #3
Count
PI
E HT29
WT
siCTRL
siCHEK1#1
siCHEK1#3
Iso Ab.
CTxB
11.68
8.20
4.41
3.92
Count
CTxB
CTxBhigh cells (%)
ns
***
***
siCTRL - + - -
siCHEK1 - - #1 #3
F HT29
OCT4
SOX2
NANOG
OCT1
KLF5
ALDH1
MYC
SOX4
KLF4
SOX9
siCTRL
siCHEK1
p-value
log2FC
0
-5
p-value
0.05
0

Figure S5

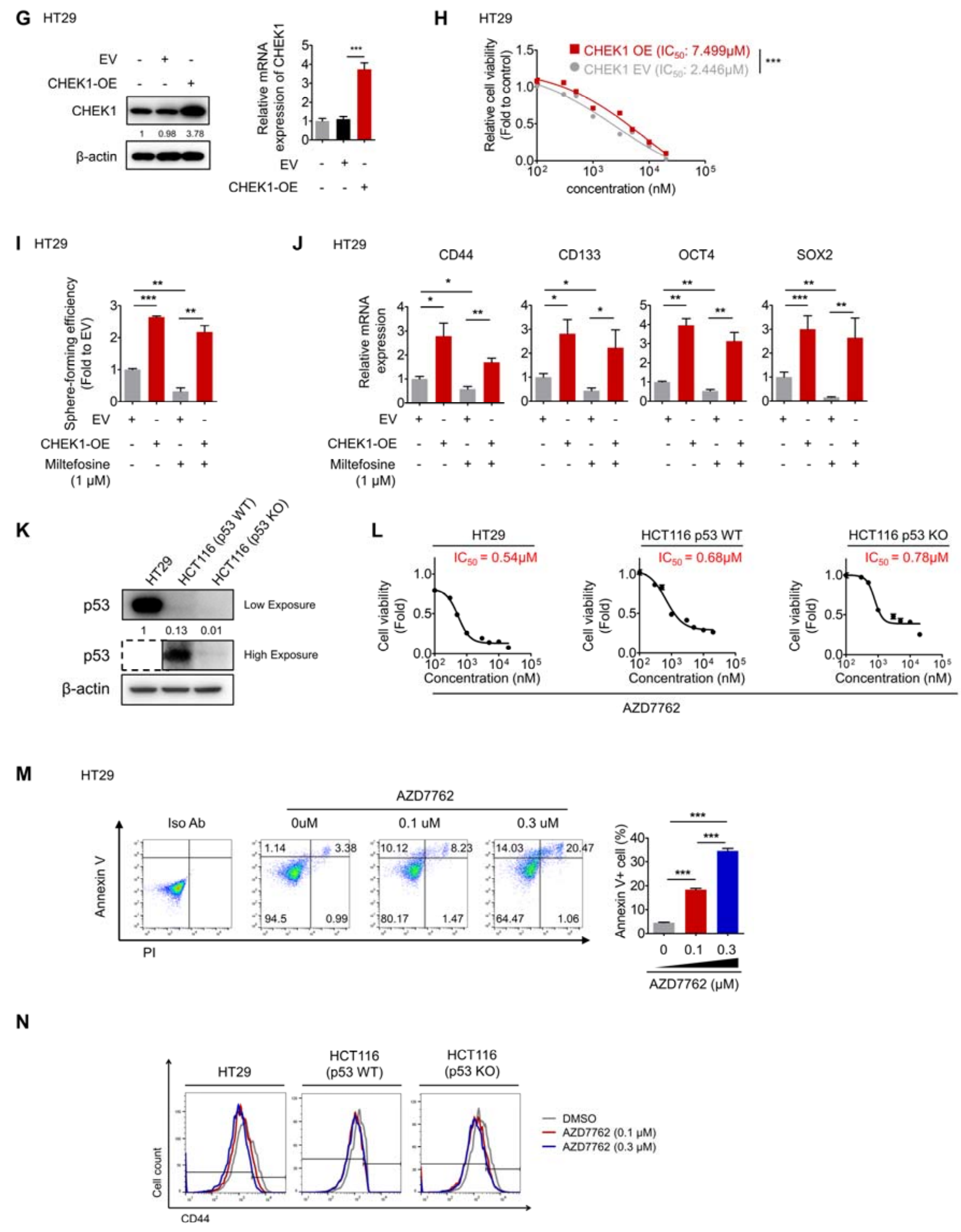

Group	CD44^{high} population (%)		
	HT29	HCT116 (p53 WT)	HCT116 (p53 KO)
DMSO	20.00 ± 0.95	20.84 ± 1.31	21.07 ± 1.07
AZD7762 (0.1μM)	12.65 ± 0.23	13.13 ± 0.48	9.82 ± 0.79
AZD7762 (0.3μM)	8.80 ± 0.75	8.16 ± 0.24	9.07 ± 0.42

Figure S6.

A) Combined flow cytometric histogram showing increase of γH2AX^{+} population upon miltefosine treatment (left). The percent of γH2AX^{+} population in bulk HT29 cells and isolated CD44^{high} populations were compared upon miltefosine treatment (1μM, 48 hours). Thin lines indicate Iso Ab-stained control samples while thick lines indicate γH2AX-stained samples. The percent of γH2AX^{+} population were presented as a bar graph (right, mean ± SEM, n=3). Statistical analysis was conducted by one-way ANOVA with Dunnett's multiple comparison test. **B**) Neutral comet assay confirming the accumulation of DNA damage upon RT exposure in both isolated CD44^{high} and CD44^{low} populations. Accumulation of DNA damage contents were compared by olive tail moments upon RT exposure between CD44^{high} and CD44^{low} populations. Bar graph (left) indicates mean values of olive tail moment score ± SEM (50 cells per treatment). Statistic values were determined by one-way ANOVA with Dunnett's multiple comparison test. **C**) Quantitative analysis of mitotic γH2AX^{+} cells and mitotic catastrophe among the p-HisH3^{+} mitotic cells upon a combination treatment of oxaliplatin, miltefosine, and AZD-7762 in the sorted CD44^{high} population of HT29 cells. Cells were treated for 48 hours. The quantifications were performed in three randomly selected fields for each specimen from a total of six independent experiments. Bar graph indicates Bar graph shows mean ± SEM (n=6/group). Statistical analysis was performed by one-way ANOVA with Dunnett's multiple comparison. **D)** Quantification of Western blot detecting protein level of CHEK1, T-CDC25c, p-CDC25c, T-CDK1, p-CDK1, γH2AX, Caspase 3 and C-Caspase3 upon combination treatment of RT, miltefosine and AZD7762. All quantified values indicate the relative band intensity of target protein normalized to β-actin as fold to control lane. Bar graphs indicates mean ± SEM (n=3/group). Statistical significances were determined by one-way ANOVA with Dunnett's multiple comparison test. **E)** Protein expression of p-Akt (Thr308 and Ser473) and Pan-Akt upon miltefosine treatment (0, 0.5 and 1 μM, 48 hours) in both HT29 and HCT116 cell lines. Values below each lane indicate the relative band intensity of target protein normalized to β-actin as fold to control lane. **F**) Comparison of p-Akt (Ser473) levels between in CD44^{high} and CD44^{low} populations. Representative 2D scatter plots (left) shows cells doubly stained with anti-p-Akt and anti-

CD44 antibodies. Bar graph (right) indicates the percent of p-Akt^{high} cells within CD44^{high} or CD44^{low} cells (mean ± SEM, n=3/group). Statistical significance was evaluated by Student's t-test. **G**) Protein expression of p-Akt (Thr308 and Ser473), Pan-Akt and CHEK1 upon various concentration of MK2206, Akt inhibitor (0, 0.5, 1, 3, 5 μM) in HT29 cells. Cells were treated for 48 hours. Values below each lane indicate the relative band intensity of target protein normalized to β-actin as fold to control lane. **H**) The promoter activity of CHEK1 upon 48-hour MK2206 treatment in sphere cultured condition for 3 days. Bar graph represents mean ± SEM (n=3/group). Statistical significance was evaluated by one-way ANOVA with Dunnett's multiple comparison test. **I)** Investigation on possible molecular mechanism between Akt and transcription of CHEK1 and mediators were performed using Ingenuity Pathway Analysis (IPA) software (Qiagen). Downstream molecules of Akt signaling and upstream molecules of CHEK1, especially transcription factors, were suggested. By leveraging the ingenuity knowledge base, 11 downstream molecule candidates of Akt signaling were suggested to activate 4 transcriptional factor candidates to regulate the expression of CHEK1. Directly linked transcriptional factor *, **, *** indicates $p < 0.05$, $p < 0.01$ and $p < 0.001$, respectively.

Figure S6

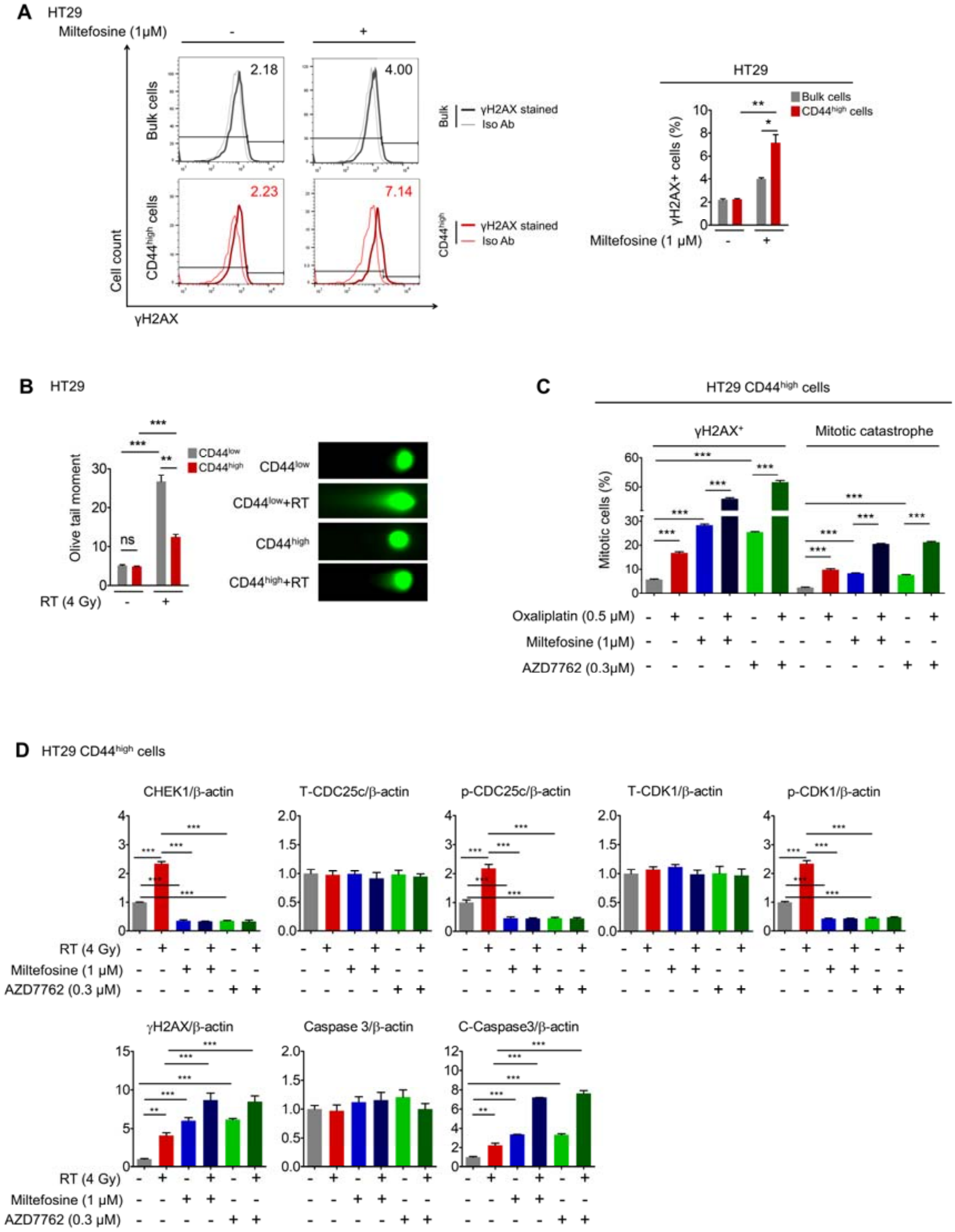
A HT29
Miltefosine (1μM)
Bulk cells
CD44high cells
Cell count
γH2AX
2.18
4.00
2.23
7.14
γH2AX stained
Iso Ab
HT29
Bulk cells
CD44high cells
γH2AX+ cells (%)
Miltefosine (1 μM)
B HT29
Olive tail moment
CD44low
CD44high
RT (4 Gy)
CD44low+RT
CD44high+RT
C HT29 CD44high cells
γH2AX+
Mitotic catastrophe
Mitotic cells (%)
Oxaliplatin (0.5 μM)
Miltefosine (1μM)
AZD7762 (0.3μM)
D HT29 CD44high cells
CHEK1/β-actin
T-CDC25c/β-actin
p-CDC25c/β-actin
T-CDK1/β-actin
p-CDK1/β-actin
γH2AX/β-actin
Caspase 3/β-actin
C-Caspase3/β-actin
RT (4 Gy)
Miltefosine (1 μM)
AZD7762 (0.3 μM)

Figure S6

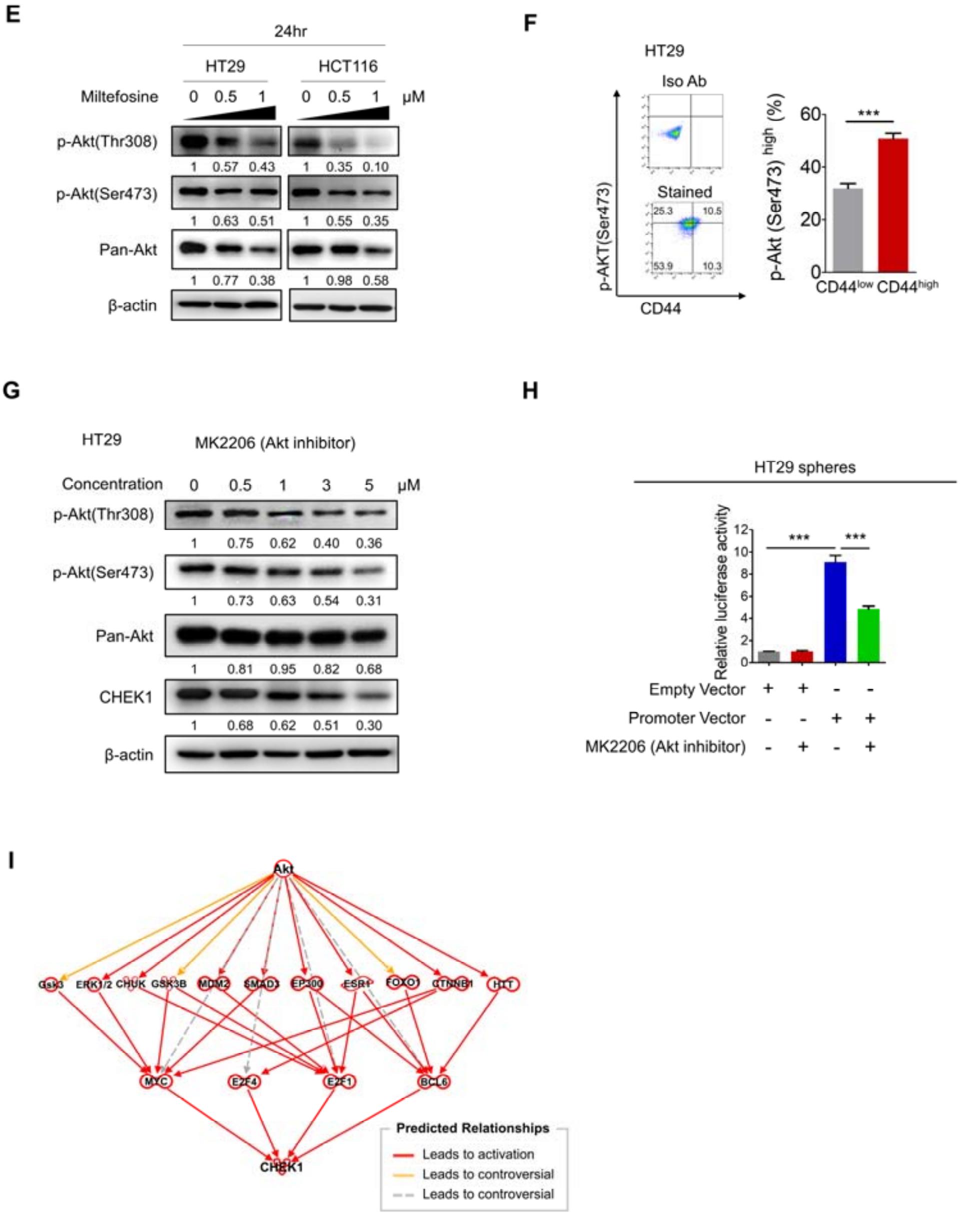
E
24hr
HT29
HCT116
Miltefosine 0 0.5 1 0 0.5 1 μM
p-Akt(Thr308)
1 0.57 0.43 1 0.35 0.10
p-Akt(Ser473)
1 0.63 0.51 1 0.55 0.35
Pan-Akt
1 0.77 0.38 1 0.98 0.58
β-actin
F
HT29
Iso Ab
Stained
25.3 10.5
53.9 10.3
p-AKT(Ser473)
CD44
p-Akt (Ser473) high (%)
60
40
20
0
***
CD44low CD44high
G
HT29
MK2206 (Akt inhibitor)
Concentration 0 0.5 1 3 5 μM
p-Akt(Thr308)
1 0.75 0.62 0.40 0.36
p-Akt(Ser473)
1 0.73 0.63 0.54 0.31
Pan-Akt
1 0.81 0.95 0.82 0.68
CHEK1
1 0.68 0.62 0.51 0.30
β-actin
H
HT29 spheres
Relative luciferase activity
12
10
8
6
4
2
0
*** ***
Empty Vector + + - -
Promoter Vector - - + +
MK2206 (Akt inhibitor) - + - +
I
Akt
Gsk3 ERK1/2 CHUK GSK3B MDM2 SMAD3 EP300 ESR1 FOXO1 CTNNB1 HTT
MYC E2F4 E2F1 BCL6
CHEK1
Predicted Relationships
Leads to activation
Leads to controversial
Leads to controversial

Figure S7. Schematic illustration of molecular mechanism of miltefosine.

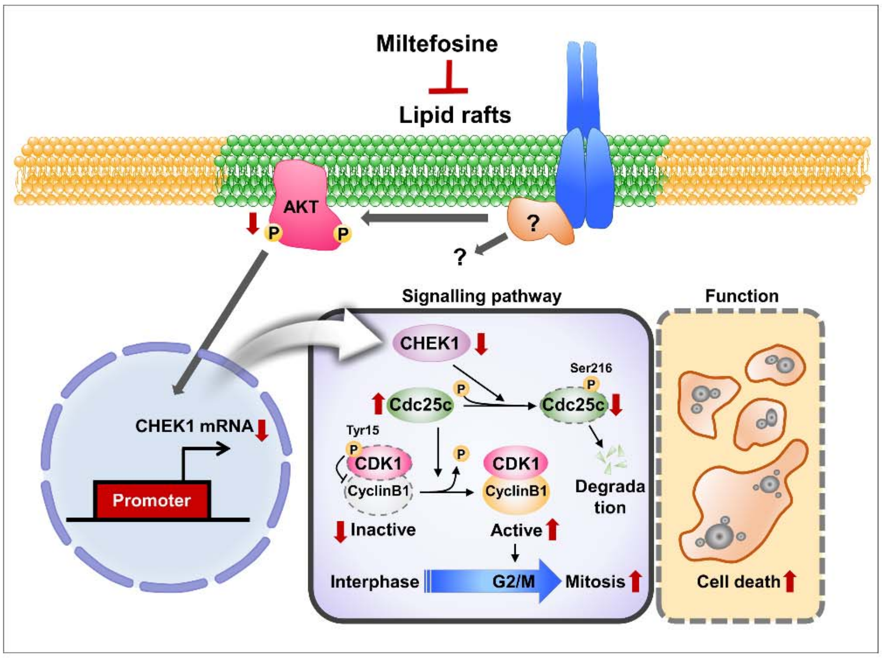

Miltefosine exhibits preferential cytotoxicity against colorectal CSCs. Mechanistically, inhibition of LR/CHEK1 axis by miltefosine releases cell cycle checkpoints and drives the inappropriate mitotic entry in the presence of unresolved DNA damage accumulation, thereby inducing catastrophic mitotic cell death in CSCs. Our findings underscore the therapeutic potential of LR-targeting APLs for CRC treatment that overcomes the therapy-resistant phenotype of CSCs.

Table S1. Clinical information for CRC patient samples used in this study

MS, Microsatellite; MSS, Microsatellite Stable; Low, Low Level of Microsatellite Instability; MSI, Microsatellite Instable; pT, pathologic T stage; pN, pathologic N stage; LN, Lymph node metastasis; diff., differentiated

Primary tumor cells from CRC patient tissues

Patient ID	Sex	Age	Diagnosis	Surgical staging (Stage)	Pathological diagnosis			
					K-ras	EGFR	p53	MS
hCRC1 (P#6441493)	M	58	Upper rectal cancer with lung metastasis	T4N2M1	Mutation	Mutation	Negative	MSS
hCRC2 (p#21257113)	M	84	Proximal a-colon cancer with liver metastasis	T3N1M1	Wild-type	Mutation	Positive	MSS
hCRC3 (P#31784993	M	67	Rectal cancer	T3N0M0	Wild-type	Mutation	Positive	MSS
hCRC4 (P#14005083)	M	45	Perforated S colon cancer with liver and lung metastasis	T4aN2bM1	Wild-type	Mutation	Positive	MSS

For Immunohistological analysis

Patient ID	Sex	Age	Tumor site	Histologic diagnosis	Histologic grade	Lymphovascular invasion	Perineural invasion	Tumor budding	Resection margin	pT stage	pN stage	Positive LNs	Total LNs
S17-12449	M	66	Sigmoid colon	Adenocarcinoma	Moderately diff.	Yes	Yes	Yes	No	pT3	pN1b	3	22
S17-7318	F	72	Sigmoid colon	Adenocarcinoma	Moderately diff.	No	Yes	No	No	pT3	pN2a	4	15

S17-6870	F	69	Sigmoid colon	Adenocarcinoma	Moderately diff.	No	Yes	Yes	No	pT3	pN1b	2	17

Table S4. Antibodies for FACs, immunofluorescence imaging and Western blotting

**Antibody List**				
**Name**	**Origin**	**Conjugation**	**Corporation**	**Cat#**
CTxB	Polyclonal Rabbit	FITC	ThermoFisher	PA1-73188
PARP	Monoclonal Rabbit	Unconjugated	Cell Signaling Technology	9532S
Caspase3	Polyclonal Rabbit	Unconjugated	Cell Signaling Technology	9662S
β-actin	Monoclonal Mouse	Unconjugated	Sigma	A5316
CD44	Monoclonal Mouse	APC-conjugated	BD Pharmingen™	559942
CD133	Monoclonal Mouse	PE-conjugated	MACS	130-080-801
CHEK1	Monoclonal Rabbit	Unconjugated	Abcam	ab40866
Flotillin-1	Polyclonal Mouse	Unconjugated	Cell Signaling Technology	3253S
Caveolin-1	Polyclonal Mouse	Unconjugated	Cell Signaling Technology	3238S
CTxB-	Polyclonal Rabbit	Peroxidase-conjugated	Invitrogen	C34780
Alexa Fluor™ 488 goat anti-mouse IgG (H+L)			Invitrogen	A11001
Alexa Fluor™ 488 goat anti-rabbit IgG (H+L)			Invitrogen	A11008
Alexa Fluor™ 555 donkey anti-mouse IgG (H+L)			Invitrogen	A31570
Alexa Fluor™ 555 donkey anti-rabbit IgG (H+L)			Invitrogen	A31572
HRP Goat anti-Mouse Ig	Polyclonal Goat	Peroxidase-conjugated	BD Pharmingen™	554002
HRP Goat Anti-Rabbit Ig	Polyclonal Goat	Peroxidase-conjugated	BD Pharmingen™	554021
rH2AX	Polyclonal Rabbit	Unconjugated	Abcam	ab11174
p-HisH3+	Polyclonal Rabbit	Unconjugated	AbwizBio	2064
T-CDC25c	Monoclonal Rabbit	Unconjugated	Cell Signaling Technology	4688S
p-CDC25c(Ser216)	Polyclonal Rabbit	Unconjugated	Cell Signaling Technology	9528S
T-CDK1	Monoclonal Mouse	Unconjugated	Abcam	ab18
p-CDK1(Y15)	Polyclonal Rabbit	Unconjugated	Abcam	ab47594
Ki67	Monoclonal Mouse	APC-conjugated	Invtirogen	17-5699-42
Ki67	Monoclonal Rabbit	Unconjugated	Invitrogen	MA5-14520

GM1	Polyclonal Rabbit	Alexa Fluor™ 488-conjugated	Invitrogen	53-6507-80
p-Akt (Thr308)	Monoclonal Rabbit	Unconjugated	Cell Signaling Technology	13038S
p-Akt(Ser473)	Monoclonal Rabbit	Unconjugated	Cell Signaling Technology	4060S
Pan-Akt	Monoclonal Rabbit	Unconjugated	Cell Signaling Technology	4685S
ALDH1A1	Monoclonal Rabbit	PE-conjugated	Cell Signaling Technology	65583S

Table S5. Small interfering RNA (siRNA) sequences

	Sense	Antisense
**Human CHEK1**		
#1	GUGGAUUUUCUAAGCACAU(dTdT)	AUGUGCUUAGAAAAUCCAC(dTdT)
#2	GUCAAAAGAAUGACACGAU(dTdT)	AUCGUGUCAUUCUUUUGAC(dTdT)
#3	GACACGAUUCUUUACCAAA(dTdT)	UUUGGUAAAGAAUCGUGUC(dTdT)

Table S6. List of primers used for RT-qPCR

Primer List		
	Forward	Reverse
PPIA	TGCCATCGCCAAGGAGTAG	TGCACAGACGGTCACTCAAA
CHEK1	CAATGTTGGCTGGAGAATTGCCGT	ATGTCTGGGATGGTGATCCTTGCT
CDC7	GCTTCATAAAGCTTCTCAATATCTTTT	TTTTTCTCCCCAGCGTGAC
EIF2AK	GCGCGGAAAGTTTGCTCAAT	GAGCTCCCAAGAAGGCAAGG
DDIT3	TGATCCAACTGCAGAGATGGC	CAGGGTCAAGAGTGGTGAAGA
CXCL8	TGATGATATAAAAAGCCACCGGA	AATCAGGAAGGCTGCCAAGAG
HES1	CTGGTGCTGATAACAGCGGA	TTTTGGAGTTCTTCACGAAAAAGA
OCT4	GGGCTCTCCCATGCATTCAAAC	CACCTTCCCTCCAACCAGTTGC
SOX2	TCGGCAGACTGATTCAAATA	CCATGCAGGTTGACACCGTT
NANOG	TGGGATTTACAGGCGTGAGCCAC	AAGCAAAGCCTCCCAATCCCAAAC
OCT1	CCCTGTCTCAGCCCATACAGA	GCTGCAAATTGGTGGTTGGAT
KLF5	CCCTTGCACATACACAATGC	GGATGGAGGTGGGGTTAAAT
CD44	GGAGCAGCACTTCAGGAGGTT	GGAATGTGTCTTGGTCTCTGGTAGC
CD133	CAGAGTACAACGCCAAACCA	AAATCACGATGAGGGTCAGC
CD44v6	GGCAACTCCTAGTAGTACAACG	GTCTTCTTTGGGTGTTTGGC
FABP1	GGAAGGACATCAAGGGGG	TCACCTTCCAGCTTGACGAC
ALPI	CCAGGACATCGCCACTCAG	TCAGTGCGGTTCCACACATA
ANPEP	CCACCTTGGACCAAAGTAAAGC	TCTCAGCGTCACCCGGTAGGA

Supplementary Table S2. Ganglioside analysis of normal vs tumor cell lines.

Attached as a separate Excel file

Supplementary Table S3. Differentially expressed gene (DEG) list for miltefosine treated HT29 cell lines

Attached as a separate Excel file.

Supplementary Table S7. Top10 signaling kinases associated with DEG upon miltefosine treatment

Attached as a separate Excel file.
